# Biophotonic probes for bio-detection and imaging

**DOI:** 10.1038/s41377-021-00561-2

**Published:** 2021-06-09

**Authors:** Ting Pan, Dengyun Lu, Hongbao Xin, Baojun Li

**Affiliations:** Institute of Nanophotonics, Jinan University, Guangzhou, 511443 China

**Keywords:** Biomaterials, Nanocavities, Biophotonics

## Abstract

The rapid development of biophotonics and biomedical sciences makes a high demand on photonic structures to be interfaced with biological systems that are capable of manipulating light at small scales for sensitive detection of biological signals and precise imaging of cellular structures. However, conventional photonic structures based on artificial materials (either inorganic or toxic organic) inevitably show incompatibility and invasiveness when interfacing with biological systems. The design of biophotonic probes from the abundant natural materials, particularly biological entities such as virus, cells and tissues, with the capability of multifunctional light manipulation at target sites greatly increases the biocompatibility and minimizes the invasiveness to biological microenvironment. In this review, advances in biophotonic probes for bio-detection and imaging are reviewed. We emphatically and systematically describe biological entities-based photonic probes that offer appropriate optical properties, biocompatibility, and biodegradability with different optical functions from light generation, to light transportation and light modulation. Three representative biophotonic probes, i.e., biological lasers, cell-based biophotonic waveguides and bio-microlenses, are reviewed with applications for bio-detection and imaging. Finally, perspectives on future opportunities and potential improvements of biophotonic probes are also provided.

## Abstract (web summary)

The emerging biological entities-based biophotonic probes, such as biological lasers, biophotonic waveguides, and bio-microlenses, have opened up an entirely new window for bio-detection and imaging with high biocompatibility.

## Graphical abstract

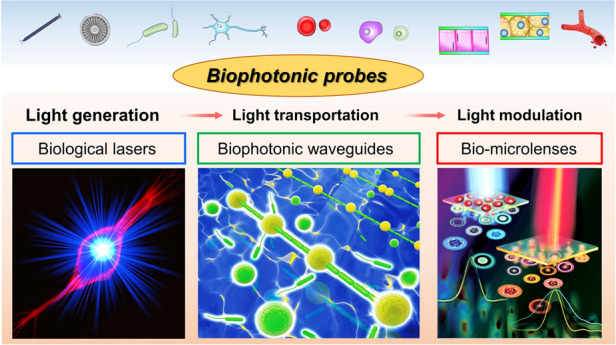

## Introduction

Sensitive detection of biological signals and precise observation of pathological changes are of great importance for the early diagnosis and treatment of infectious diseases, cancer, and other health disorders. However, owing to the low quantity of biochemical signals and complex microenvironment in biological systems, the detection of the targets of interest is challenging. Fortunately, the prosperous development of optical and photonic technologies in recent years provides many choices for optical detection and imaging, holding great promises for real-time visualization of biological signals in complex biological structures and processes^1,2^. Optical detection exploits optical responses, such as light absorption, scattering, fluorescence, and reflectance, induced by biophysical/biochemical changes for bio-identification and disease diagnosis^3^. Due to the inherently label-free nature, optical detection is a powerful alternative to conventional detection techniques (e.g., mass or electrochemical)^4–7^. With optical detection techniques, real-time signals of a wide range of biological test samples (from molecular biomarkers to pathogens and cells, and even to tissues and organs) can be obtained in a non-invasive manner with high-sensitivity and high-resolution^8–12^. To date, optical detection and imaging have been demonstrated to be one of the most powerful technologies for detection of biological signals and for diagnosis of various diseases, including infectious diseases, cancer as well as other disorders^13–18^.

For a precise and flexible optical detection in a biological microenvironment, photonic probes with micro/nanostructures are always desirable. For this purpose, the selection of appropriate optical materials is certainly crucial, since the probing performance largely depends on their chemical and mechanical properties, optical functionalities as well as biological performances^19^. To date, the most commonly used materials for the assembly of versatile photonic components and photonic probes are mainly based on inorganic materials such as silica glass^20–23^, or organic polymers such as polymer nanowires^24,25^. Because of their excellent optical properties, such as high transparency and suitable mechanical strength, these materials have been applied for nanophotonic integrated devices in diversified fields of application^26^. For example, optical waveguides based on silica optical fibers have been widely studied and were even used for implantations in animal bodies, particularly fiber-optic implants in the brain for optogenetic studies^27,28^. However, the main disadvantage of these photonic components based on traditional materials is low biocompatibility and biodegradability, which greatly limit their potential in biomedical applications. High biocompatibility of a material is a fundamental requirement for in vivo applications, which demands the absence of toxicity and low health threat to the living systems^29^. Moreover, high biocompatibility also refers to the biofunctionalities that the implants can perform their expected functions in vivo. Additionally, biodegradability is another essential requirement, since the materials can be degraded and metabolized by the body without the need for additional operations to remove the implants.

With abundant natural biomaterials and biological entities, Mother Nature always inspire us to design photonic structures and probes to manipulate light^30^. Indeed, living cells and microscopic organisms as well as their derivates such as DNA, proteins, silk and cellulose et al., show different capabilities to interact with light, and can further serve as different photonics devices such as waveguides, microlenses, gratings, and even lasers^31–37^. These natural biomaterials and biological entities hold huge promise for creation of new photonic probes for bio-detection, imaging, and therapeutic applications^19^. They inherently possess excellent biological performances, including noninvasiveness, high biocompatibility, biodegradability, and resorbability. Moreover, another interesting feature of biological entities such as virus, cells and tissues is their ability to serve simultaneously as optical devices and diagnostic specimen, which facilitate further real-time detection and imaging in biocompatible microenvironments. Therefore, instead of bio-derived materials and biomolecules (such as proteins and nucleic acids), biophotonic probes introduced in this review are mainly focused on large biological entities, such as virus, bacteria, fungi, algae, mammalian cells and tissues. By translating biological principles into man-made designs, these biophotonic probes offer a seamless interface between optical and biological worlds^38,39^.

**Fig. 1:**
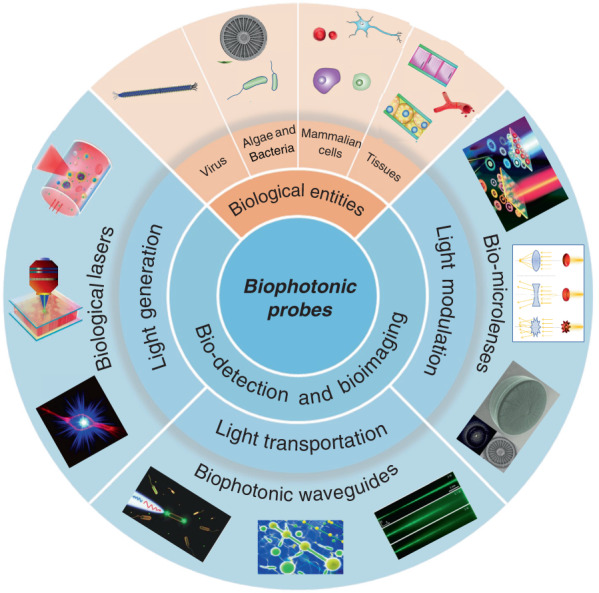
Overview of biophotonic probes for bio-detection and imaging. Based on biological entities, such as virus, algae & bacteria, mammalian cells, and tissues. Such photonic probes include biological lasers, biophotonic waveguides, and bio-microlenses, with optical functions from light generation, to light transportation and light modulation. Biological entities: virus^88^, copyright 2017, Springer Nature. Diatom^154^, Copyright 2014, Optical Society of America. Tissues^69^, copyright 2017, The Royal Society of Chemistry. Blood vessel^167^, Copyright 2019, American Chemical Society. Biological lasers: top^50^, Copyright 2016, Optical Society of America. Middle^69^, Copyright 2017, The Royal Society of Chemistry. Bottom^81^, Copyright 2018, The Royal Society of Chemistry. Biophotonic waveguides: left^143^, Copyright 2017, WILEY‐VCH. Middle^134^, Copyright 2015, WILEY‐VCH. Right^138^, Copyright 2017, American Physical Society. Bio-microlenses: top^175^, Copyright 2019, American Chemical Society. Bottom^154^, Copyright 2014, Optical Society of America

While the progresses in functional biophotonic structures based on bio-inspired and naturally-derived biomaterials as well as synthetic materials can be found in several reviews^19,30,40,41^, there still lack of a review on biophotonic probes based on large biological entities, such as viruses, cells and tissues. However, there is a growing number of interests on biological entities-based photonic probes that have significantly enriched the society of biophotonics, with huge potential in biomedical and healthcare applications. In this review, we focus on recent progress on biophotonic probes based on biological entities, with much focus on viruses, cells and tissues, and their biomedical applications, in particular, for bio-detection and imaging. We emphatically and systematically introduce three representative biophotonic probes, i.e., biological lasers, cell-based biophotonic waveguides and microlenses, possessing different optical functions from light generation to light transportation and light modulation (Fig. 1). Their fabrication techniques, optical functionalities, and biomedical applications are provided (Table 1). We also discuss the remaining challenges and future perspectives of these biophotonic probes for biomedical applications.

**Table 1:** Summary of biophotonic probes for bio-detection and imaging introduced in this review

Type	Function	Biological entities	Example	Applications	Advantages
Biolasers	Light generation	Virus	M13 phage	Bio-detection^45^	Much higher sensitivity than traditional antibody-based techniques, such as ELISA
		Bacteria	GFP expressing *E. coli*	Bio-imaging^61,66^	Good photostability, self-replenishing by stable expressing GFP
		Mammalian cells	Human cells stained with endogenous fluorescent proteins or biocompatible dyes	Bio-imaging^64,65,67–73^	Much narrower linewidth, stronger light intensity, superior spectral and spatial resolution than fluorescence imaging
			Human cells internalized with WGM cavities or nano-sized cavities	Cell tagging and tracking^71,73,80,101,103,104^, intracellular sensing^81,82,109^	Real time tracking of thousands of individual cells, label-free, non-invasive, all-optical recording of transient intracellular bioactivities
		Tissues	Tissues/blood injected with fluorescent dyes (random lasing)	Cancer diagnosis^52,92,93,95–98^, blood diagnosis^50^, bio- sensing^52,94,99^	High biocompatibility, easy manipulation, without external cavities
			Tissues inserted with hard/soft microbeads	Bio-imaging^100^	Without external cavities, narrowband emission
			Fluorophores-stained tissues sandwiched in a high-Q FP microcavity (LEM)	Cancer diagnosis^49,56,105–107^, bio-imaging^69^, intracellular sensing^108^	High specificity by labeling specific biomarkers, > 100-fold detection sensitivity over traditional fluorescence-based methods
Biophotonic waveguides	Light transportation	Bacteria	*E. coli* chains assembled by optical trapping	Light guiding^38,133^, self-sensing and detection^143^	Good light propagation performance, non-invasive, flexible in manipulation
			Other bacteria cell chains, such as *L. acidophilus* and E. *faecalis*	Bio-imaging and fluorescence signal detection^39,144^	Bio-friendliness, real time detection at subwavelength spatial resolution,
		Microalgae	Cyanobacteria suspensions	Light transmission^138^	Nonlinear self-trapping, deep light propagation
			Diatom probes formed by optical trapping	Bio-imaging^141^, cellular force sensing^142^	Non-invasive, able to image and detect arbitrary surface topography
		Mammalian cells	RBCs suspensions	Light transmission^139,140^	Non-invasive, harmless to the viability of cells
			RBCs chains assembled by optical trapping	pH sensing^145^	Real time detection of blood pH with an accuracy of 0.05, in vivo assembly
Bio-microlenses	Light modulation	Microalgae	Cyanobacteria, volvox	Light focusing^149–151^	Sensing light
			Diatoms	Light focusing^153–157^	Living photonic crystals
		Bacteria	Yeast cell	Bio-imaging^168^, fluorescence enhancement^170^	Subdiffraction-limit imaging, operating in a contact manner, real-time detection
		Mammalian cells	RBCs	Bio-imaging^147,162–164,167^, blood diagnostics^171^, cellular phenotyping^172–175^	Tunable lensing behavior by varying the refractive index and shape of the cells, high biocompatibility

## Biological lasers

To realize their potential biomedical applications of photonic probes, effective control and modulation of light generation are particularly important in various biochemical environments, especially in cells and deep tissues in vivo. In this regard, the unique properties of light emitted by the lasers, including high intensity, directionality and monochromatic emission, have rendered lasers one of most useful tools in biomedical applications^42^. The first clinical application of lasers was conducted by Maiman in 1960, which demonstrated the damage to eyes and skins by the ruby laser with high-intensity^43^. Since then, the exploration and development of lasers has opened up new directions for biomedical applications, and numerous laser-based biomedical devices have been routinely used in the clinic.

Biological lasers (biolasers), utilizing naturally-derived biomaterials as part of the cavity and/or gain medium in a biological system, represent an emerging class of laser^44^. These biolasers avoid the biohazards of conventional laser devices. Biolasers implanted or injected in cells or tissues can serve as photonic probes for detection and imaging of various biological signals at the molecular^45–47^, cellular^48,49^, and tissue levels^50–52^. Particularly, biolasers based on cells and tissues can serve as highly sensitive tools for bio-detection and imaging, since their optical output is tightly related to the biological structures and activities of the biological systems. In this section, we firstly describe the principle of biolasers, then emphatically introduce biolasers based on biological entities, particularly, virus, cells and tissues, and finally discussed the recent progress on biomedical applications for bio-detection and imaging.

### Principle of biolasers

In general, a laser system is mainly composed of three parts: an optical cavity for light trapping, a gain medium for amplifying light in the cavity, and a pump energy to powering the system^32,42,53^. The photons will be amplified after repeated interactions with the activated gain medium. Laser emission only occurs when the amount of available excited gain molecules becomes higher than the total loss in the cavity, i.e., above the lasing threshold^44^, which can be defined as^54^,

1 $$n_1\sigma _e\left( \lambda \right) = n_0\sigma _a\left( \lambda \right) + \gamma _c$$

Where *n*_*1*_ and *n*_*0*_ represents the amount of the gain molecules in the excited and ground state, respectively. *σ*_*e*_ and *σ*_*a*_ are the emission and absorption cross-section of the gain molecules at the lasing wavelength (*λ*), respectively. The lasing wavelength of operation is specifically determined by the gain molecules used in the cavity. And *γ*_*c*_ represents the loss coefficient of the cavity.

The quality factor (*Q*-factor) is a measure of the damping effects of the resonator modes, and is defined as the ratio of the stored energy to the energy dissipated per radian of the oscillation^55^. The *Q*-factor is inversely proportional to the loss coefficient of the cavity (*γ*_*c*_). A high *Q*-factor means a lower concentration of gain molecules and lower pump energy are required to reach the threshold, which leads to a narrow linewidth in the spectrum. The intrinsic spectral linewidth of the cavity can be defined as *∆λ*_*c*_ = *λ*/*Q*.

**Fig. 2:**
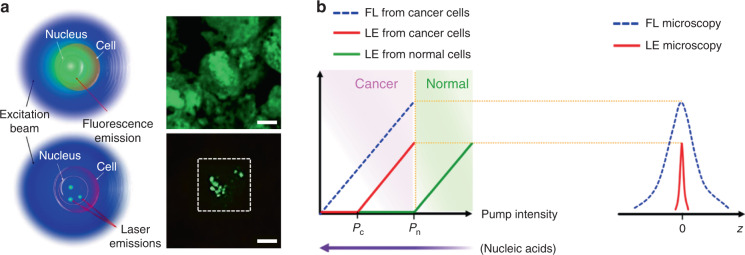
Comparison between fluorescent probe and lasing probe. **a** Schematic and confocal microscope image of cells stained with fluorescence probe (top) and lasing probe (bottom). **b** Laser emission (LE) has a much narrower emission profile than fluorescence (FL)^56^. Copyright 2017, Springer Nature

As compared to traditional fluorescence emission, lasing probes have been demonstrated in biosystems with much narrower linewidth, stronger light intensity, higher sensitivity, superior spectral and spatial resolution, owing to the unique optical feedback mechanism and threshold of laser (Fig. 2)^56^.

The typical feature of a biolaser is that the gain medium is composed entirely/in part of biological entities, such as cells, tissues, and virus. Since the lasing output performances are closely coupled to the gain medium, the biological changes in the gain medium can be readily detected by biolasers with high sensitivity. The choice of gain materials should be cautiously considered when designing biolasers, to fulfill the requirements of biocompatibility and biodegradability. Representative examples for biocompatible gain materials are fluorescent proteins and biological dyes. Fluorescent proteins, obtained from a variety of living organisms, possess excellent biocompatibility, good photostability and high quantum yields^57^. Laser emission by fluorescent proteins, such as green fluorescent protein (GFP), monomeric Cherry (mCherry), Venus and indocyanine green (ICG), have been achieved by using various types of cavities^44,50,58–61^. As an alternative to fluorescent proteins, biological dyes found naturally in human tissues and other species, such as luciferin and riboflavin (vitamin B2), have been exploited as a gain material for biolasers^62,63^. By incorporating these biocompatible fluorophores into virus, cells, and tissues, biolasers can be generated with these biological entities serving as biological gain materials.

### Cell-based biolasers

**Fig. 3:**
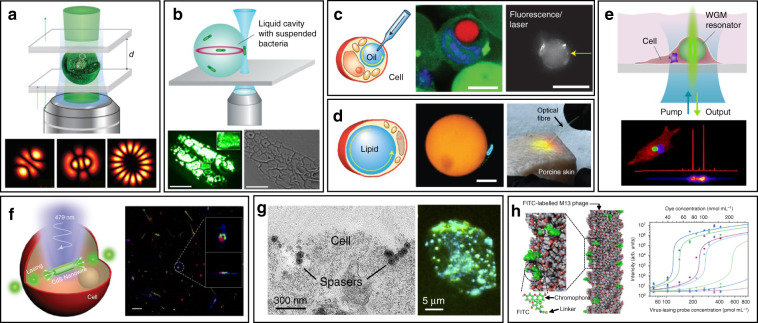
Cell/virus-based biolasers. **a**, **b** Extracellular cell lasers. **a** Extracellular cell laser based on GFP-expressing mammalian cell^65^. Copyright 2013, Springer Nature. **b** Extracellular cell laser based on GFP-expressing bacterial cells. Top^61^, Copyright 2014, The Royal Society of Chemistry. Bottom^66^, Copyright 2011, Optical Society of America. **c**–**e** Intracellular lasers based on WGM microcavities. **c** Intracellular oil droplets as a soft WGM cavity^80^. Copyright 2015, Springer Nature. **d** Lasing in adipocyte achieved with endogenous lipids^80^. **e** Intracellular polystyrene microsphere as WGM resonator^73^. Copyright 2015, American Chemical Society. **f**, **g** Intracellular lasers based on nano-sized cavities. **f** Intracellular semiconductor nanowire laser. Left^81^, Copyright 2018, The Royal Society of Chemistry. Right^104^, Copyright 2020, The Optical Society. **g** Intracellular spasers^82^. Copyright 2017, Springer Nature. **h** Virus laser based on M13 bacteriophage^45^. Copyright 2019, Springer Nature

Cell-based lasers can be grouped into two categories according to the location of the resonator: extracellular lasers (resonator structure located outside the cell) and intracellular lasers (resonator structure internalized by the cell). Extracellular lasers are commonly constructed with a Fabry–Pérot (FP) cavity by sandwiching fluorophores-stained cells between two highly reflective mirrors^56,64,65^. The first demonstrations of extracellular has been demonstrated with GFP-expressing live mammalian cells placed inside a FP resonator in 2011 (Fig. 3a)^65^. Optical signals were emitted by the cell laser with a pulse energy down to nJ-range, which was orders of magnitude lower than the onset of light-induced cell damage. Moreover, the lasing emission spectra was consisted of multiple narrow-band peaks. This allowed cell phenotyping by identification different spectral components. Similarly, *E. coli* bacteria stably expressing GFP have been integrated in both microdroplet cavities^61^ and biofilms^66^ to serve as living gain medium (Fig. 3b). When providing sufficient nutrients for the steady-state growth of *E. coli*, GFP are stably expressed and functional, thus the gain medium can be self-replenished. Although the use of endogenous fluorescent proteins is found to be an attractive option for providing optical gain in cells, in practice, however, the long transfection procedure for fluorescent protein expression in cells is inconvenient and time-consuming.

Compared to endogenously expressed fluorescent proteins, a much greater variety of synthetic fluorescent molecules such as 5-chloromethylfluorescein diacetate (CMFDA)^67,68^, fluorescein sodium salt^64^, Rhodamine 6G^69,70^, Calcein-AM^71^ and fluorescein isothiocyanate (FITC)^72^, have been demonstrated as convenient gain medium to generate cell lasers from various cell types. Particularly, the fast dye staining procedures can be completed within an hour, which dramatically reduced the laser preparation time as compared to the time-consuming fluorescent protein transfection methods. Furthermore, lasing in a wide variety of wavelengths from green to red can be achieved depending on the dyes and mirrors used^73^.

When the whole resonator structure, along with dye gain medium, is internalized by the cell, a stand-alone cell laser (intracellular laser) can be achieved. Whispering gallery modes (WGMs), existing within spherical optical resonators, are well known for their applications in precise physical measurements and label-free bio-chemical sensing down to single molecule level^74–76^. WGM cavity is usually more compact than other cavity structures, with relatively low threshold due to the high Q-factor (>10^7^)^77^. Such spherical optical resonators with micro- and nano-sized structures can be easily internalized by cells and serve as ideal candidate for intracellular lasers. Commonly, most of WGM lasers possess multi-mode lasing because of the lack of mode-selection strategies. One possible strategy to acquire single-mode lasing is to retain only one resonant mode from the multi-mode lasing through reducing the size of the cavity and extending the free-space range^78^. But this strategy will lead to reduced round-trip gain and higher threshold. Recently, Gu *et. al* demonstrated a simple and general approach to realizing single-mode WGM lasing in polymer bottle microresonators by engineering the pump intensity to modify the spatial gain profiles of the bottle microresonator surface^79^. When their mode intensity profiles are spatially overlapped with the pump stripes, single-bottle WGMs can be efficiently selected to lase. Since single-mode WGM lasing is challenging to achieve, most intracellular WGM lasers are based on multi-modal lasing.

WGM intracellular lasing was firstly demonstrated by Humar et al., where dye-doped oil droplets were used as soft WGM cavity within a cell (Fig. 3c)^80^. Additionally, a lipid droplet with a large diameter of ~40 μm, naturally existing within adipocytes (fat cells), was also applied as an inherent WGM cavity to generate intracellular laser (Fig. 3d)^80^. Without any foreign synthetic resonator, prominent lasing was achieved within adipocytes by simply staining the lipid droplet with lipophilic fluorophores. Moreover, the authors realized lasing within tissues by exciting adipocytes in the porcine subcutaneous adipose tissues using an optical fiber. The feasibility of WGM intracellular lasing was further demonstrated by introducing dye-doped lasing microparticles, both soft polymer microspheres and hard microbeads, into virous types of cells^48,73^. For instance, polystyrene microparticles with diameter from 5 and 10 μm were internalized by different kind of cells via endocytosis (Fig. 3e)^73^. The output spectra enabled the determination of the particle diameter with 50-pm precision, which can serve as a unique identification code for cell tagging and intracellular sensing.

The relatively large size of micrometer-sized WGM cavities may potentially affect cellular bio-functions when designing intracellular lasers. Therefore, it becomes attractive to develop smaller cavities with sub-micrometer or even nanometer scale for intracellular internalization and lasing. For example, Wu et. al. incorporated cadmium sulfide (CdS) semiconductor nanowire (NW) into cytoplasm, where the NW simultaneously served as FP cavity owing to the large difference of refractive index between the NW and the intracellular microenvironment (Fig. 3f)^81^. Intracellular sensing was achieved by detecting the wavelength shift of lasing peaks, showing a relatively high sensitivity of 55 nm per RIU (refractive index units). In another study, a relatively small nanosphere laser (spaser, or plasmonic nanolaser), with particle size of only about 22 nm, was used to generate laser emission directly in living cells and tissues (Fig. 3g)^82^. The spaser was generated by a plasmonic gold nanoparticle core coated with a silica shell containing organic dye molecules. The lasing spectra emitted by the spaser had extremely narrow linewidth, only about 1 nm in width. Under single nanosecond pulsed laser pumping, tracking of cancer cells both in vitro and in vivo was achieved with high sensitivity due to the narrow linewidth and high spectral intensity of intracellular spaser lasing.

### Virus-based biolasers

Viruses are the most ubiquitous living organisms on earth. In general, the fundamental structure of virus is their genetic material (RNA or DNA) encapsulated by a protein shell, called capsid^83^. Among all viruses, bacteriophages (phages) have been gaining widespread attention, which is a diverse group of viruses infecting bacteria^84^. Using phage display techniques, a wide variety of peptides/proteins with diverse biological functions can be precisely and orderly displayed on the surface of phage capsids^85^. Owing to their availability, stability and biosafety, phages have recently emerged as versatile tools for biomedical applications^86–90^. Recently, an innovative phage-based biolaser for bio-detection has been reported using M13 phage, a kind of filamentous phage displaying a rod-like architecture with a diameter of 7 nm and a length of 900 nm (Fig. 3h)^45^. The host cell of M13 phage is F pilus expressing strains of *E. coli* bacteria, which means they are safe to human body. By chemical conjugating fluorescent dyes on the surface of M13, virus laser was generated with adjustable lasing output owing to the repetitive arrangement of M13. Taking advantage of phage display technology, M13 could be modified with different target binding specificity to detect a wide range of biomolecules of interest without the need to previously immobilize the antibody on a surface. Laser emission from M13 allowed high detection sensitivity up to 90 fmol mL^−1^. These virus lasing probes hold great potential to replace traditional antibody-based techniques in bio-detection with higher precision and detection speed.

### Biolasers in tissues

In addition to cell/virus lasers, lasing in tissues is of more implications for clinical practice, since tissues composed of cells and surrounding extracellular matrix can better mimic the complex physiological environment in vivo. For this purpose, many attempts have been made to generate tissue lasers during the last few years. Taking advantage of the scattering properties of tissues, lasing in tissues can be conveniently generated through random lasing by direct injection of fluorescent dyes^91,92^. Random lasing has been demonstrated in different kinds of tissues stained with various fluorescent dyes, including bovine heart and bone^52,93^, rat muscle and brain^94,95^, human colon, kidney and cancerous tissue^92,96,97^. Since the output characteristics of random lasing are closely coupled with the structure and microenvironment of the tissues, lasing spectra can be used as a useful tool to distinguish malignant from healthy tissues^98^, to probe subtle structural alterations^52^, and to sense biological changes^99^. Random lasing provides relatively convenient methods to generate tissue lasers without need to introduce any external cavities. However, the uncertain lasing threshold and spectral peaks may also seriously affect the detection accuracy and sensitivity^32^.

**Fig. 4:**
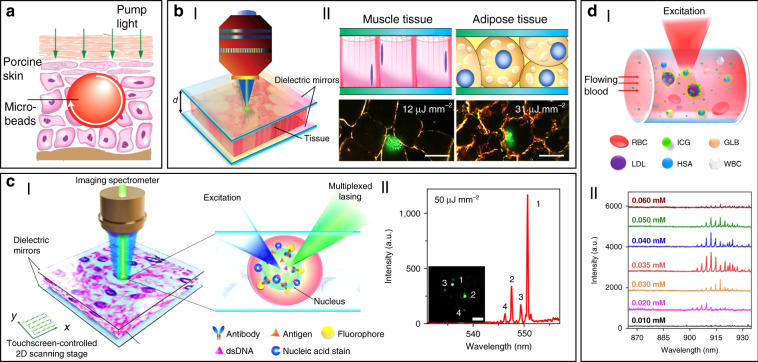
Biolasers in tissue. **a** Schematic illustration of a stand-alone WGM laser in skin^100^. Copyright 2017, Optical Society of America. **b** Tissue laser sandwiched within a high FP cavity^69^. Copyright 2017, The Royal Society of Chemistry. **c** Laser-emission-based microscope (LEM)^56^. Copyright 2017, Springer Nature. **d** “Blood laser”. ^50^ I: Schematic view of blood laser realized in human blood. II: Spectrum of the “blood” lasing with various ICG concentrations (0.01–0.06 mM) in human whole blood. Copyright 2016, Optical Society of America

In addition to random lasers, implantable and stand-alone WGM lasers have also been demonstrated in tissues by inserting solid microbeads or liquid microdroplets in the cornea and skin^100^. As shown in Fig. 4a, porcine skin tissue was implanted with a dye-doped polymer microbead at a depth of about 100 μm using a tattoo machine. When optically pumped by an external 532 nm laser, a clear lasing emission was observed. As compared to random lasing in tissues, WGM lasers have much more narrower linewidths (<0.2 nm) and significant lower lasing thresholds on the order of tens of nJ per mm^2^. However, such tissue lasing still fails to identify and detect specific biomarkers within tissues, which is of great significance to truly provide bioanalytical and imaging capabilities for popularizing the clinical applications of biolasers.

To solve these problems, Fan’s group has developed a versatile tissue lasing platform named as laser-emission-based microscope (LEM), which uses a high-*Q* FP cavity composed of two highly reflective mirrors together with fluorophores-stained tissues sandwiched in between (Fig. 4b)^69^. Prominent lasing emissions from human tissues stained with biocompatible fluorescent dyes, such as muscle or adipose, were observed with quite low threshold intensity down to 10 μJ mm^−2^. To further generate lasing in specific cells within a tissue, the biomarkers (specifical proteins or nucleic acids) within cells were labeled by fluorophore-labeled antibodies or nucleic acid dyes (Fig. 4c)^56^. By integrating with a 2D raster-scanning stage, laser emitted by the labeled biomarkers enabled acquisition of mapping or imaging of tumor tissues obtained from patients with lung and colon cancers. This technique was used for discriminating cancer tissues from normal tissues with a high resolution of <700 nm, and even diagnosis of lung cancer at early-stage was achieved. This LEM holds great potential in precision medicine by taking advantages of their high intensity, high background suppression and high spectral/spatial resolution.

Beside solid tumors and organs, lasing can also be achieved in blood. For instance, by using a human-safe near-infrared dye indocyanine green (ICG), lasing could be achieved in whole human blood flowing in an optofluidic ring resonator (OFRR) capillary^50^. In this scenario, ICG with biocompatible concentration lower than 0.04 mM was found to bind to low-density albumins (GLB) and lipoproteins (LDL) in blood to generate ICG lasing (Fig. 4d). Lasing in human blood was also achieved by directly dispersing dye-doped polymer microbeads into blood^100^. The lasing efficiency in blood showed no obvious difference with that operated in water, indicating that blood components such as red blood cells did not weaken the lasing effect. Such blood lasing provides many possibilities for blood diagnosis with clinical and biomedical applications.

### Applications for bio-detection and imaging

As described above, biolasers based on cells/tissues enable delivery of optical energy in situ to biological systems with simultaneously spectral and temporal optical localization. These biolasers can serve as photonic probes in a range of biomedical applications, including cellular tagging and tracking, diagnostics, intracellular sensing, and novel imaging.

**Fig. 5:**
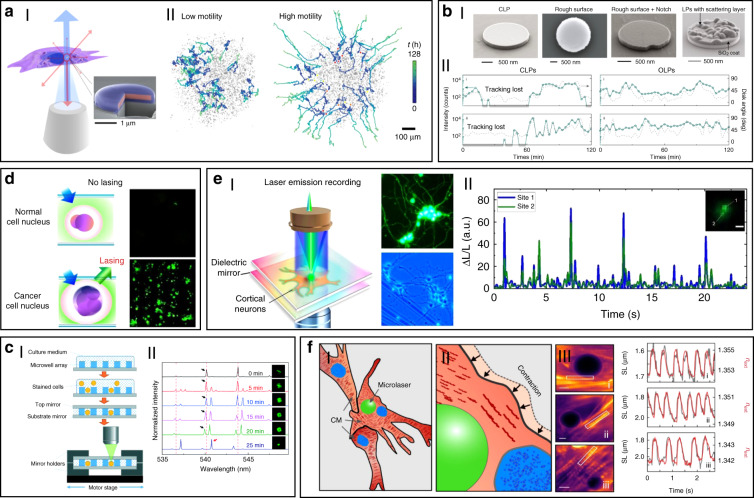
Biolasers for bio-detection and imaging. **a** Biolasers for cell tagging and tracking. I: illustration of a semiconductor microdisk-based laser internalized into a cell^71^. Copyright 2018, Springer Nature. II: cell tracking in a tumor spheroid^101^. Copyright 2019, Springer Nature. **b** Microdisk lasers with omnidirectional emission for long-time cell tracking^103^. I: SEM images of conventional microdisk lasers (CLPs) and modified microdisk lasers. II: comparison of cell tracking by CLPs and Omnidirectional-emitting laser particles (OLPs). Copyright 2021, Springer Nature. (**c**) Cell laser array for identification of abnormal cells^49^. Copyright 2017, The Royal Society of Chemistry. **d** LEM for cancer diagnosis^56^. Copyright 2017, Springer Nature. **e** Laser recording of subcellular neuron activities by LEM^108^. Copyright 2020, American Chemical Society. **f** Schematic illustration and monitoring of contractility in neonatal mouse cardiomyocytes by intracellular laser-based contraction sensor^109^. Copyright 2020, Springer Nature

Using the unique spectra signals of intracellular lasers, individual cells can be distinguished and tracked among large groups of cells. For example, Fikouras et al.^71^ and Martino et al.^101^. incorporated WGM microdisk lasers into a range of different type of cells (Fig. 5a). Microdisks with slightly different diameters resulted in obviously different lasing output spectra, thus enabling tagging and tracking of individual cells. Since each cell could uptake multiple microdisks owing to their small size, wavelength-multiplexed spectrum signals from a single cell were achieved, so that large cell populations could be uniquely tagged at the same time. In an in vitro 3D tumor tissue model, thousands of tumor cells with different motility were respectively tagged and clearly tracked. It also showed their excellent stability and high biocompatibility in cells and tissues. However, the output emission of these disc-shaped laser particles is inherently directional, mainly in the plane of the cavity resonance^102^. For cell tracking, this intrinsic feature can cause random intensity fluctuations and hinder the optical reading of lasers, since the orientation of laser particles in cells is arbitrary and randomly changed as the cell moves. To solve this problem, Tang et al. modified microdisk lasers with rough surface, boundary defects or scattering layers to generate light scattering for omnidirectional emission (Fig. 5bI)^103^. These modified omnidirectional microlasers achieved continuous tracking of moving cells for 2 h, which was difficult to achieve for traditional direction-dependent microlasers owing to the frequent signal loss (Fig. 5bII). These intracellular lasers based on microdisks are useful and flexible biophotonic probes for cell tagging and tracking, especially in the field of noninvasive analysis of cell migration in caner invasions and immune response.

Cell-based biolasers can also be combined with other imaging techniques to fulfill the precise tracking of the trajectory of individual single cells. For example, Li et al.^104^ constructed a dual-modality imaging system by combining nanowire lasing with optical coherence tomography (OCT), a clinically available imaging technology with ultrahigh-resolution and excellent imaging depth. The CdS nanowire internalized by cells provide both OCT and lasing signals. With this dual-modality imaging system, they tracked single cell migration trajectories in the subretinal layer of living New Zealand rabbits. This study demonstrates the feasibility of multi-modal imaging by combining laser emission-based imaging with other imaging techniques.

The laser spectral information is also closely coupled with physiological changes in cells or tissues at single cell or tissue level, which can be used for biomedical diagnosis, especially cancer diagnosis. For example, cell lasers formed by a cell array was generated by integrating microwell array with high-quality FP cavities (Fig. 5c)^49^. Real-time detection of spectral peak wavelength shift and the lasing threshold enabled the identification of abnormal cells deviating from a large number of healthy cells. At tissue levels, by directly injecting fluorescent dyes into tissues, random lasing enabled distinguishing the tumor tissues from normal cells by the lasing mapping^96,98^. However, the sensitivity of random lasing in tissue is undesirable due to the unpredictable lasing peak. Recently, LEM has been employed in screening lung cancer tissues obtained from human patients with high sensitivity and specificity^56^. By labeling cells with fluorochrome-conjugated tumor markers, the significant differences in lasing thresholds between tumor cells and normal cells allowed tumor detection with high sensitivity up to 97.5% (Fig. 5d). To date, this versatile diagnosis platform has been applied to detect multiple different types of cancers, including breast cancer, colon cancer, kidney cancer and gastric cancer, and the tested samples can be prepared by various methods, such as direct frozen or formalin fixed paraffin-embedded samples^69,105–107^.

Cell-based biolasers can also perform as a useful probe for real-time detection of subtle physiological activities in living cells, which is difficult to realize by other conventional techniques. For example, LEM technique was applied to detect the subtle changes in intracellular ion concentration at nM level in single neurons as well as neural networks (Fig. 5e)^108^. This “neuron laser” showed over 100-fold detection sensitivity improvement when compared with conventional fluorescence detection methods. In another study, cell lasers were demonstrated to be a potential sensing probe to detect the changes in intracellular osmotic pressure^64^. Very recently, Schubert et al. reported an intracellular laser-based contraction sensor by integrating microscopic WGM lasers into cardiac cells (Fig. 5f)^109^. This laser-based sensor enabled all-optical detection of the transient contractile activities of cardiac cells with subcellular resolution and high sensitivity. This technique provides huge potentials for different biomedical applications, such as long-term tracking of individual cardiac cells, recording contraction profiles in vivo and at organ-scale. The use of novel intracellular lasers as noninvasive, bio-integrated sensing probes emerges as powerful platforms for monitoring various physiological activities in living cells with subcellular resolution.

### Limitations and potential improvements

Although biolaser-based photonic probes are still in their infancy, it is clear that these biophotonic probes hold huge potential for wide scope of biomedical applications. However, as an emerging technology, it still faces a few challenges. More effort and new approaches are still in demand to design and develop different forms of biolasers. Some of the limitations and potential improvements are given in below.

First, efficient lasing emission only occurs above the lasing threshold. Therefore, the intensity of external pump or the concentration of gain molecules has to be high enough if the *Q*-factor of the cavity is relatively low. However, the external pump and concentrated fluorophore in cytoplasm or tissues may cause non-negligible damage and toxicity to cells and tissues. Undoubtedly, development of better cavity with higher *Q*-factor and lower threshold are highly desirable, which can reduce the biological damages of pumping light and fluorophore to living cells and tissues.

Second, currently, most of biolasers are excited by UV–vis light (190–700 nm) and NIR‐I light (700–950 nm). Although NIR‐I light exhibits less absorption and scattering by the biological tissue and can penetrate deeper in tissues than UV–vis light, the penetration capability of NIR‐I light is limited to a depth of about 10 mm into the subcutaneous tissues^110^. Consequently, the bio-detection and imaging using biolasers can hardly achieved in deep tissues of the body. Considering that the excitation light wavelength is basically determined by the gain molecules, design and development of gain materials which can be excited in the NIR‐II region (1000–1700 nm) may contribute to deep tissue applications. Another possible solution is to combine biolasers with biocompatible optical waveguides, which can break the tissue penetration limit of light by transporting light into deep tissues. Besides, the combination of biolasers with other novel techniques deserves further exploration to improve detection and imaging in deep tissues. For example, two-photon imaging technique offers less optical damage to cells and tissues, the NIR light absorption is also confined to the focal plane, thus improving optical sectioning in tissues^42^.

## Cell-based biophotonic waveguides

Controlled guiding and transportation of light to target region in biomedical systems plays an important role in biological and biomedical applications, such as bio-sensing and biomedical diagnosis. But the depth of light penetration at visible and near-infrared wavelengths in biological media and tissues is quite limited, because of the light scattering in the tissues^110^. As fundamental components for photonic integration, optical waveguides play irreplaceable role for light propagation within well-defined structures. For biomedical applications, optical waveguides can break the tissue penetration limit of light by transporting light into deep tissues. Generally, solid-state materials such as silica glass (SiO_2_) and hard plastics, are most commonly used to generate optical waveguides, however, lack of biocompatibility and biodegradability^111^. Moreover, the mechanical fragility of silica glass makes it invasive to living organism. Therefore, biophotonic waveguides that are elastic, biocompatible, and biodegradable are highly desirable to interface with biological systems for further biomedical applications.

Among many natural biomaterials, living cells hold huge potential for in situ formation of biophotonic waveguide that meet the above requirements within biological systems with the ability for light propagation. In this section, we review biophotonic waveguides formed with different biological cells by diverse fabrication techniques. Living cell-based biophotonic waveguides provide desirable optical properties for light guiding and more importantly, they are totally biocompatible and flexible as compared with traditional synthetic materials. The potential of living cell-based biophotonic waveguides for bio-detection and imaging was also discussed. Some representative cell-based biophotonic waveguides and the critical parameters are summarized in Table 2.

**Table 2:** Representative cell-based biophotonic waveguides and the critical parameters

Cell type	Refractive index	Assembly method	Propagation loss [dB μm^−1^]	Maximum size (length × diameter)	Maximum propagation distance	Ref
*E. coli* bacteria	1.39	Optical trapping	0.23–0.27	41.4 μm × 0.5 μm (assembled by 980 nm laser with a power of 50 mW)	41.4 μm (675/532 nm laser with a power of 40 μW)	^38,133^
*L. acidophilus* bacteria	1.40	Optical trapping	Not mentioned	23.5 μm × 0.3 μm (assembled by 808 nm laser with a power of 15 mW)	23.5 μm (532/644 nm laser)	^39^
*E. faecalis* bacteria	1.39	Optical trapping combined with optofluidic	0.025~0.035	360 μm × 0.8 μm (assembled by 980 nm laser with a power of 60 mW)	360 μm (532 nm laser with a power of 3 mW)	^144^
Human RBCs	1.38-1.44 (determined by osmotic condition)	Optical trapping combined with optofluidic	0.07~0.11^a^	64 μm × 1 μm (assembled by 980 nm laser with a power of 25 mW)	64 μm (532 nm laser with a power of 100 μW)	^145^
		Optical force mediated nonlinear optical effect	1.8 × 10^−^^4^ ~2.3 × 10^−^^4a^	3 cm × 60 μm (assembled by 532 nm laser with a power of 350 mW)	3 cm (532 nm laser with a power of 350 mW)	^139^
Sheep RBCs	1.40	Optical force mediated nonlinear optical effect	4.6 × 10^−4^ ~5.4 × 10^−^^4a^	3 cm × 100 μm (assembled by 532 nm laser with a power of 550 mW)	3 cm (532 nm laser with a power of 550 mW)	^140^
Cyanobacteria	1.38	Optical force mediated nonlinear optical effect	1.4 × 10^−4^ ~2.3 × 10^−4a^	4 cm × 270 μm (assembled by 532 nm laser with a power of 3 W)	4 cm (532 nm laser with a power of 3 W)	^138^

^a^Specific values of propagation loss are not directly given in these studies, these values are estimated from the experimental data reported in the literatures according to the following formula^38^: $\alpha = - 10\lg \left( {\frac{{I\left( x \right)}}{{I_0}}} \right)/x$, where α is the attenuation, *x* is the propagation distance, *I(x)* is the optical power at the distance *x*, and *I*_*o*_ is the initial optical intensity

### Bio-inspired light-guiding

**Fig. 6:**
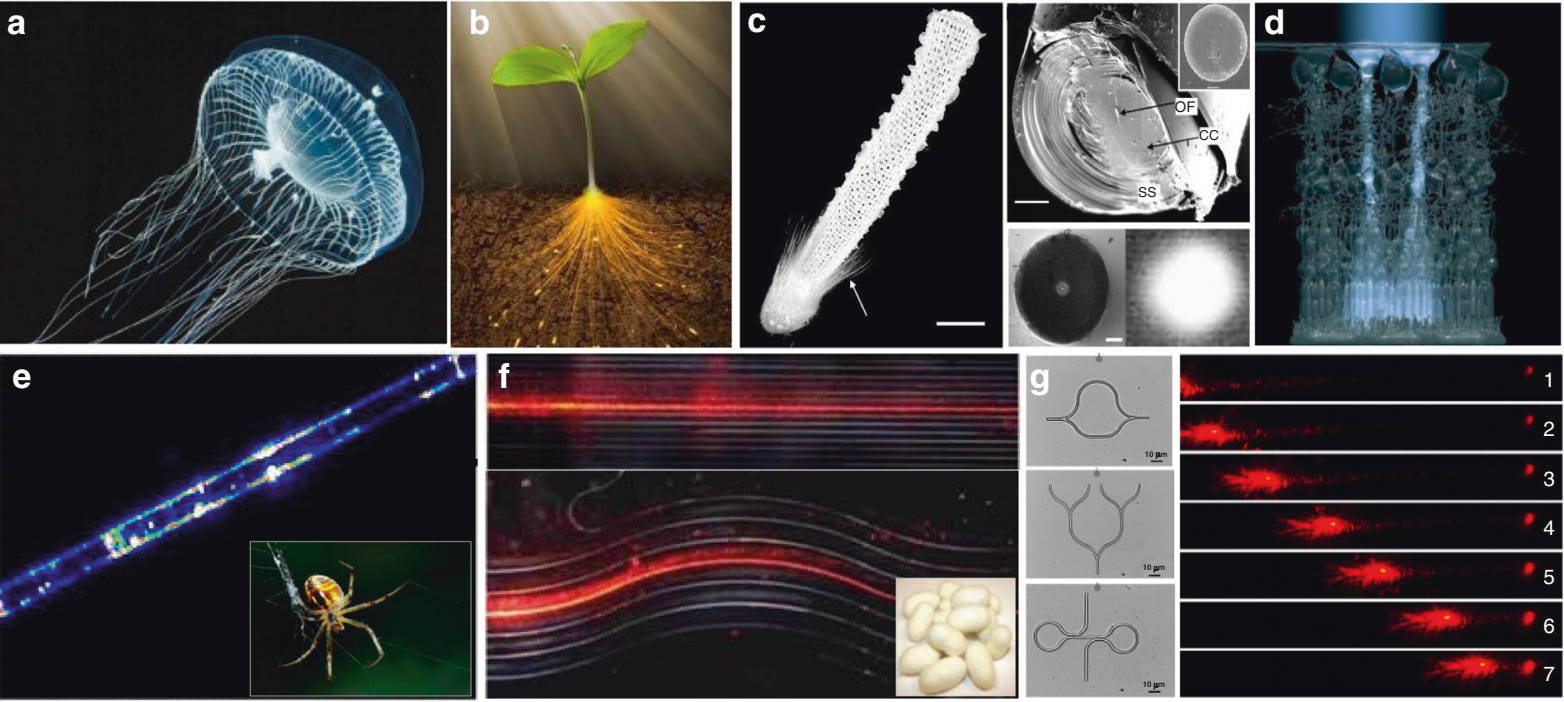
Light-guiding in living organisms and naturally-derived biomaterials. **a** Bioluminescence light of a jellyfish guided through its optical-fiber-like tentacles^113^. Copyright 2010, Annual Reviews. **b** Illustration of light guiding through the stem of a plant to the roots^114^. Copyright 2016, American Association for the Advancement of Science. **c** Light guiding in spicules of sponge *Euplectella*^115^. Copyright 2003, Springer Nature. **d** Müller cells act as ‘optical fibers’ guiding light from the retinal surface to the photoreceptor cells^116^. Copyright 2007, National Academy of Sciences. **e** Light guiding along a spider silk fiber^126^. Copyright 2013, AIP Publishing LLC. **f** Optical waveguides produced by silkworm silks^123^. Copyright 2009, WILEY‐VCH. **g** Protein-based optical waveguides^127^. Copyright 2015, WILEY‐VCH

In nature, specific types of optical waveguide structures for light-gathering and light-guiding can be widely observed in both plants and animals. For instance, jellyfish uses its tentacles with fiber-like structures to guide bioluminescence light, serving as a bait to attract prey (Fig. 6a)^112,113^. The vascular system of plants, including leaf and stem, can serve as natural optical waveguides to receive and transmit sunlight to their roots (Fig. 6b)^114^. The spicules of the deep-sea sponge *E. aspergillum* show remarkable fiber-optical properties similar to commercial glass fibers (Fig. 6c)^115^. In the retina of vertebrate eyes, light must penetrate multiple cell layers before reaching photoreceptor cells. Interestingly, Müller glial cells, whose major function is to facilitate connections between nerve cells in the retina, are demonstrated to serve as optical waveguides to transport light from the surface of the retina to the midsection of photoreceptor cells through their tubular cell body (Fig. 6d)^116,117^.

In addition to the waveguiding capability of natural structures in living systems, naturally derived biomaterials, such as cellulose, silks and endogenous proteins, are also excellent candidates to serve as the materials to form optical waveguides^118–120^. These biomaterials show excellent optical properties, including high transparency and low transmission loss^121–125^. For example, native spider silk filaments showed the ability of light guiding, although the light propagation loss was relatively high (Fig. 6e)^126^. Silkworm silks produced by *Bombyx mori*, which have been extensively used for 5000 years, were used to produce microscale optical waveguides with controllable structure and composition (Fig. 6f)^123^. Beside silks, endogenous proteins were fabricated into all-protein nanowire-based optical waveguides with various complex micro/nanoarchitectures, showing good light propagation capability along the waveguide structures (Fig. 6g)^127^.

### Cell-based biophotonic waveguide

The discovery of light-guiding capability in living organisms enlightens researchers to design biophotonic waveguides by living cells. Principally, for single-material waveguides, the refractive index (RI) of the optical material must be higher than that of the surrounding media for total internal reflection to occur^128^. Larger RI contrasts between the material and the surrounding media help to minimize waveguide losses. Although the RI of an optical material vary in principle at the atomic and molecular level, it is practically defined as a spatially averaged quantity over the dimension of the optical wavelength. The RI (*n*) of a living cell can be expressed as^129^

2 $$n = n_{\rm{dry}}\left( {1 - W} \right) + n_{\rm{water}}W$$

where *n*_dry_ (≈1.51) is the RI of cell’s dry mass, *n*_water_ (≈1.33) is the RI of water, and *W* is the water content of the cell. For biological cells with a typical water content *W* of about 0.7, the RI of the cell is about 1.38. Actual measured RI of various biological cells at visible wavelengths are in the 1.38–1.41 range (Table 2), which is slightly larger than that of water. Thus, light guiding through a chain of cells is allowed by total internal reflection at the interface of the cell membrane and the water.

**Fig. 7:**
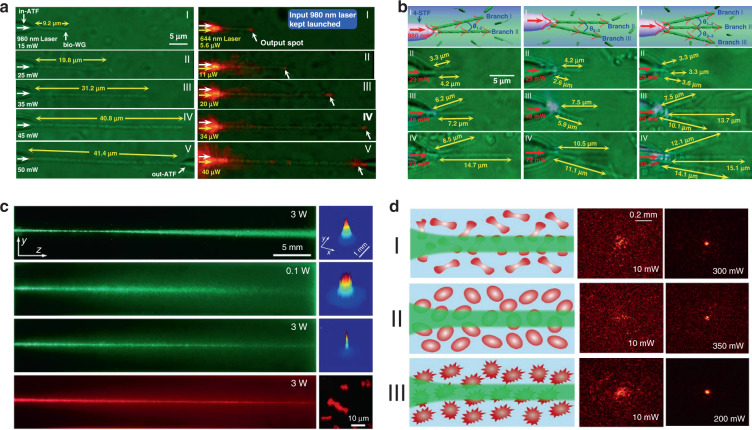
Biophotonic waveguides formed by living cells. **a** Bacteria-based biophotonic waveguide formed by optical trapping^38^. Copyright 2013, American Chemical Society. **b** Forming of *E. coli*-based branched optical structures^133^. Copyright 2015, WILEY‐VCH. **c** Nonlinear self-trapping and guiding of light through optical assembled cyanobacteria-based biophotonic waveguide in seawater^138^. Copyright 2017, American Physical Society. **d** Self-trapping and guiding of light through assembled *human* RBC waveguides under different osmotic conditions (I: isotonic, II: hypotonic, and III: hypertonic suspensions)^139^. Copyright 2019, Springer Nature

The capability of individual cells for light guiding enables the assembly of biophotonic waveguide based on multiple living cells. Optical trapping offers a noninvasive approach for the manipulation of different targets in an on-demand manner^36,130^, making it possible to assemble multiple cells. For example, Xin et al. demonstrated the assembly of multiple cells into cell chains via optical trapping, either by extended optical gradient force^131^ or the cooperation of optical scattering and gradient force^132^. Using *E. coli* bacteria as an example, they reported the optical formation of bacteria-based biophotonic waveguides in aqueous solution using an abrupt tapered optical fiber (ATF) (Fig. 7a)^38^. Since the refractive index of *E. coli* cells (around 1.39) is slightly higher than that of water (around 1.33), total internal reflection of light occurs at the interface of cell membrane and water. Therefore, light propagation is allowed through cell chains over tens of microns. In their scenario, using laser light launched by ATF at a wavelength of 980 nm, biophotonic waveguides were formed by assembling a chain of *E. coli* cells. The length of biophotonic waveguides was adjustable according to the number of cells assembled under the control of the laser intensity^38^. Beside constructing single biophotonic waveguides by single-branched ATF, multiple *E. coli*-based biophotonic waveguides has also been fabricated by using a four-segment tapered optical fiber (4-STF) through optical trapping (Fig. 7b)^133^. During the experiments, the formed multiple branched cell-based waveguides allowed flexible movement without destroying the structures of cell chains. Further, even in a dynamic environment with perturbations, these multiple cell-based biophotonic waveguides were able to maintain good stability and reversible assembly. The robust structures provide potential for transportation of a single light beam to multiple target sites in biological systems. In addition to *E. coli* bacterial cells, this optical trapping method is also applicable for assembly of many different biophotonic waveguides using different cell types^134^. The optical trapping technique offers a highly biocompatible and flexible strategy to assemble optical devices from living cells. More importantly, the living cells can directly sense and detect biological signals, thereby eliminating the need to introduce invasive and harmful synthetic optical materials. One major drawback is that the length of these biophotonic waveguides is limited to below 100 μm, which hampers the biomedical applications that need long-range light propagation and delivery.

It is generally believed that the strong scattering/absorption loss of cells makes it difficult for light to propagate long distances in a biological environment. However, nonlinear optical effects have been demonstrated to overcome this limitation and enhance the propagation of light in scattering media. For instance, nonlinear optical techniques have been applied to achieve stable long-distance propagation of light with low loss in the scattering media such as colloids and nanoparticle suspensions^135–137^. Interestingly, several recent studies have demonstrated nonlinear optical response as well as enhanced transmission of light can also be achieved in otherwise lossy biological suspensions of living cells, including algae^138^ and red blood cells^139,140^. Bezryadina et al. investigated light propagation through assembled cyanobacteria, where a chain of cyanobacteria serves as a waveguide. In their scenario, due to the optical gradient force by the high-intensity laser beam, cyanobacteria were trapped to the center of laser beam instead of spreading separately in seawater. Consequently, the nonlinear effect enables light transportation along the assembled cyanobacteria over a much longer distance in the centimeter scale. Such long-distance light transmission is due to the higher refractive index of cyanobacteria (around 1.38) than seawater (around 1.33). The authors also found that the efficiency of light transportation by cyanobacteria depended not only on the laser power, but also on the surrounding medium environment. As shown in Fig. 7c, with a low optical power, after cell assembly, light was linearly scattered with a wide divergence area by the cyanobacteria^138^. As the optical power was increased, however, light propagation was transited from linear diffraction to nonlinear self-trapping. Unlike in seawater, in a glycerol-rich medium, cyanobacteria aggregated into clusters to diffuse light rather than conduct light. Comparable experiments with human and sheep red blood cells (RBCs) suspensions were performed by the same group^139,140^. They observed waveguide formation as well as deep penetration of light in the human RBC suspensions under different osmotic conditions (Fig. 7d). It is worth noting that the RBC cells maintained good viability throughout the experiments. These studies demonstrate that the nonlinear effect of light transportation adjusted by the combined optical forces exerted on the cells is indeed helpful towards creating of waveguides with much longer propagation distance in biosuspensions. Such long-distance light transmission holds great potential for biomedical applications that need deep light propagation such as imaging and diagnosis of deep tissues.

### Biomedical applications

**Fig. 8:**
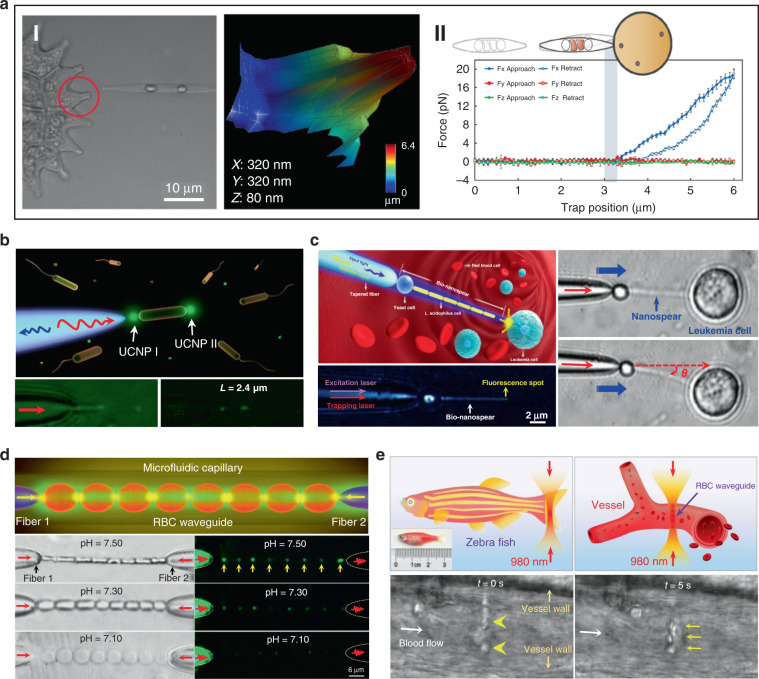
Cell-based biophotonic waveguides for bio-detection. **a** Optically trapped living diatom probe for bio-detection and imaging. I: biophotonic probe to image arbitrary surface topography of living cells^141^. Copyright 2011, IOP Publishing Ltd. II: living diatom probe for cellular force sensing^142^. Copyright 2012, American Chemical Society. **b** Single-bacterium labeling and self-detection via optical trapping of a single bacterium^143^. Copyright 2017, WILEY‐VCH. **c** Biophotonic waveguide-based probe for single cell probing^39^. Copyright 2018, American Chemical Society. **d** RBC waveguide for pH sensing^145^. Copyright 2019, WILEY‐VCH. **e** Optical assembly of the RBC waveguide inside a living zebrafish^145^

Biophotonic waveguides made from biological cells hold irreplaceable advantages for light transportation in biological systems, since they are naturally biocompatible and flexible, which greatly reduce the biohazards to living organism. Like the natural structures in living organisms, individual cells also show the capability for light guiding. This light guiding capability by single cells prove a significant step towards building biophotonic probes for different biomedical applications such as enhanced non-invasive imaging and biosensing in biological environments. For example, living cells of the diatom *Nitzschia Acicularis* were used as imaging and force probes formed by optical trapping (Fig. 8a)^141,142^. This biophotonic probe was applied to image arbitrary surface topography. As an example, a side view of the soft algal colony resting on the substrate was obtained by the diatom probe, which was unobtainable through other conventional imaging techniques, such as atomic force microscope (AFM) (Fig. 8aI)^141^. This technique provides a simple but effective tool to image highly curved or soft surfaces of living samples in their native environments. Further, this diatom active probe can serve as sensing probes to detect cellular force at the nanoscale^142^. As shown in Fig. 8aII, upon touching the surface of a tumor cell, the diatom probe was able to translate and rotate. Consequently, the force and torque responses of the diatom’s center-of-mass was recorded to measure the sub-picoNewton scale forces exerted on the cell membrane. The single cell-based probe can also be used for self-sensing. For example, *E. coli* cell was optically trapped between two upconversion nanoparticles (UCNPs) for single cell detection (Fig. 8b)^143^. Due to its light-guiding property, the trapped bacterium was labeled by the single UCNPs emitting green light. The guiding light along the cell body enables the detection and sizing of the trapped bacterium itself via the detected back-scattering optical signal in real time.

Beside single cell, biophotonic waveguide-based probe formed by cell chains have also been applied for bio-detection and imaging in biological systems. Li et al. fabricated a cell-based biophotonic waveguide with a length of ~13 μm and a radius of 200 nm, which was composed of a yeast cell and a chain of *L. acidophilus* cells assembled by optical trapping using a tapered fiber (Fig. 8c)^39^. This biophotonic waveguide was performed as a biophotonic probe for cell imaging and fluorescence signal detection. Single leukemia cells stained with fluorescent dye was scanned in human blood. Remarkably, the biophotonic probe was highly flexible and deformable, since it would rather bend itself than destroy the cell membrane when touching cells. In another study, Wu et al. assembled a cell-based biophotonic waveguide formed by a chain of optically trapped *E. faecalis* cells in a microfluidic chamber^144^. With a maximum length up to 360 μm and propagation loss down to 0.03 dB/μm in biological medium, the biophotnic waveguide allowed long-distance light-guiding and submicron focusing. The authors also explored its potential as a biophotonic probe by detecting the backscattered signals from RBCs, which was expected to facilitate biomedical sensing and single cell analysis.

Biological cells can be applied not only as building blocks of the biophotonic waveguide, but also as the testing samples simultaneously. Since the shape and refractive index of living RBCs are closely coupled with the physiochemical properties of the membrane and surrounding environments, biophotonic waveguides formed by RBCs provide a potential detection technique for diagnosis of blood related disorders. Li et al. designed a biophotonic probe based on RBC waveguide for blood pH sensing (Fig. 8d)^145^. RBC waveguide was assembled in an optofluidic channel, where two tapered optical fiber were inserted from opposite directions to launch laser beam at a wavelength of 980 nm for cell trapping. They proved that the modes of light transmission through the biophotonic waveguide were tightly associated with the morphology of RBCs, which depended on blood pH. Therefore, by detecting the light propagation mode in the cell-based biophotonic waveguide, real-time monitoring of the pH values of blood was achieved with an accuracy of 0.05. In addition, they further validated its potential for in vivo applications, in situ constructing an RBC waveguide inside the blood vessels of living zebrafish (Fig. 8e). The RBC-based biophotonic waveguide brings new opportunities for accurate biosensing, particularly diagnosis of pH-related blood disorders.

### Limitations and potential improvements

There is no doubt that the introduction of living cell-based biophotonic waveguides represent a new direction for biomedical sensing and detection in biological systems with high biocompatibility. However, they also have significant drawbacks that hamper further clinical applications. The major limitations and potential improvements are listed below.

First, the fabrication of living cell-based biophotonic waveguides with long-length remains elusive, due to the small sizes of biological cells. Furthermore, the viability and bio-functionality of biological cells should remain intact during the fabrication process, so that the methods are strictly limited to generate living cell-based biophotonic waveguides. Therefore, more efforts are needed to explore new approaches to fabricate long living cell-based biophotonic waveguides under biocompatible conditions.

Second, the penetration depth of the light delivery along the formed living cell-based waveguides is still limited, which is a challenge for in vivo applications. New techniques for overcoming scattering loss will help improving the transmission efficiency to reach deeper tissues in the body.

Third, although living cell-based biophotonic waveguides assembled by optical force mediated nonlinear optical effect have achieved deep light propagation up to 4 cm, the formed waveguides suffer from instability due to the weak connecting force between cells. Therefore, it is almost impossible to flexibly manipulate and move these waveguides, which dramatically limits their further biomedical applications. On the contrary, biophotonic waveguides assembled by optical trapping using the tapered fiber can be flexibly moved in aqueous solution along the fiber tip. However, the length of this kind of waveguides is limited to a few hundred micrometers, which hampers the biomedical applications that need long-range light propagation into deep tissues. The combination of these assembly techniques with other assistive techniques may help constructing flexible cell-based biophotonic waveguides with long-range light propagation for bio-detection and imaging within deep biological environments such as tissues.

## Cell-based bio-microlenses

Optical lenses are important optical devices designed for light modulation, i.e., focusing or dispersing light, and are widely used in different applications from microscopy to laser processing. In nature, photonic structures with refractive index less than two are building blocks of many living organisms, which hold promise to generate biological microlenses^30,122,146^. Interestingly, living biological cells, as naturally abundant biomaterials with inherently excellent biocompatibility, can also confine light in biological systems, acting as bio-microlenses^147^. In this section, we will introduce recent advances in living cell-based bio-microlenses.

### Lens effects of living cells

**Fig. 9:**
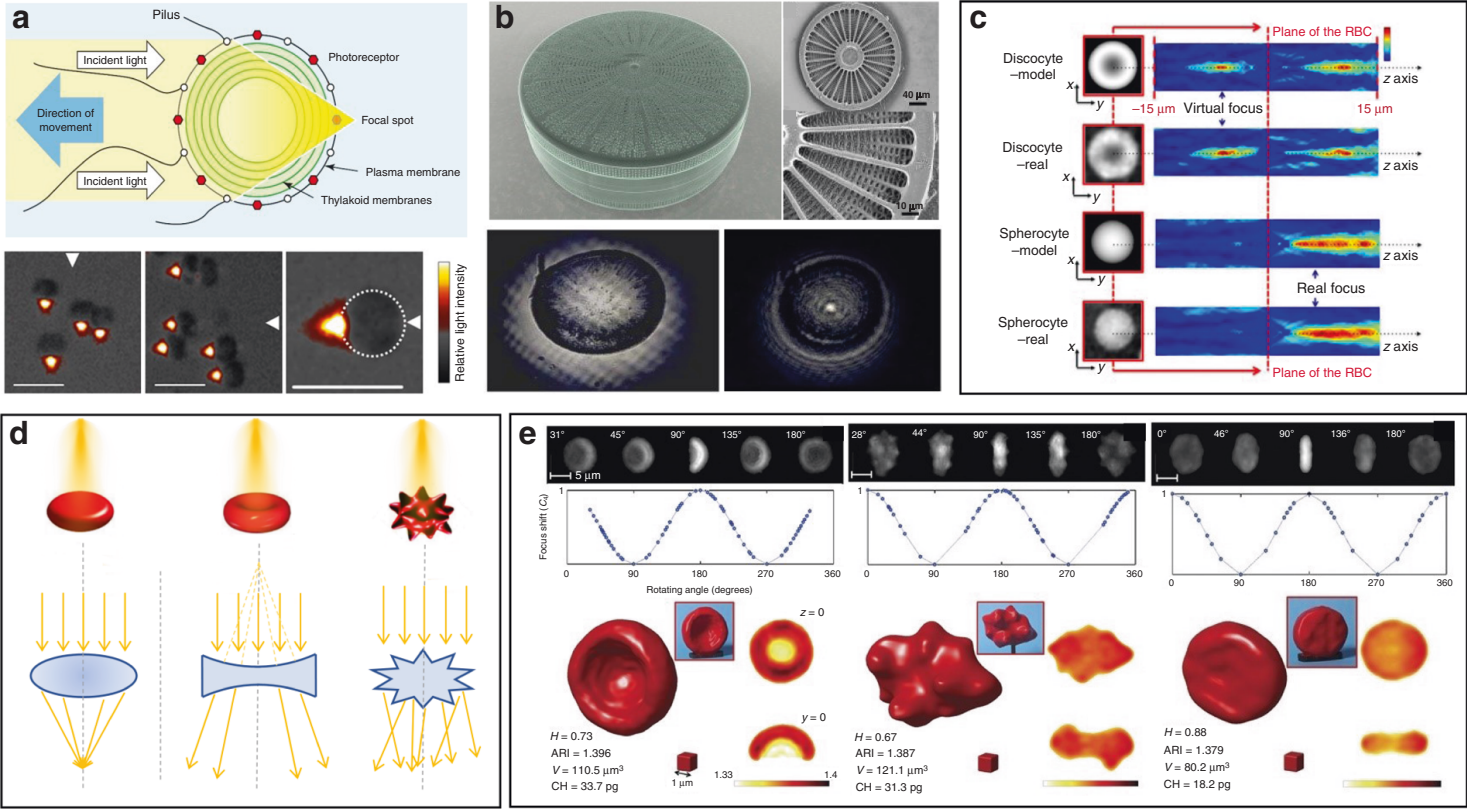
Microlens effects of living cells. **a** Illustration of microlens effect of Synechocystis (top)^151^. Copyright 2016, Elsevier. Bottom, images of synechocystis cells focusing light at the opposite side of the light source^151^. Copyright 2016, Elife. **b** Light focusing effect of diatom. Top^154^, Copyright 2014, Optical Society of America. Bottom^153^, Copyright 2007, Optical Society of America. **c–e** RBC behaves as a bio-lens. **c** The phase-shifts in plane (x–y) for a single RBC and correspondent light intensity distributions in the plane (x–z)^147^. Copyright 2015, Springer Nature. **d** Illustration of spherical-like RBC behaves as a convergent lens, discocyte as divergent lens, echinocyte as a scattering object^162^. Copyright 2015, SPIE. **e** Tomographic 3D reconstructions of RBCs. From left to right: stomatocyte, echinocyte and iron-deficiency anemia^164^. Copyright 2017, Springer Nature

Some photosynthetic bacteria and microalgae exhibit “phototaxis” behavior that involves moving in response to local light conditions. For example, cyanobacteria of the genus *Synechocystis*, exhibit positive phototaxis, driving themselves towards the light source with the mobility of pili on the side of cells facing the light source^148^. However, the method by which such small cells can focus light resulting in directional movement has remained puzzling. Researchers have recently revealed that their phototaxis was probably caused by the lens behavior of the spherical cells^149,150^. Schuergers et al. firstly demonstrated the lens effect in cyanobacteria (Fig. 9a)^151^. They experimentally demonstrated that cyanobacteria were able to act as spherical microlenses, confining light into a focal spot near the plasma membrane at the rear side of to the light source. Photoreceptors on the cell membrane sensed the light spot light and drove pili pulling at the side close to the light source, thus dragging cells towards the light source. This research reveals that the cyanobacteria are likely to be the smallest and oldest example of biological cells serving as bio-microlens on earth.

Other organisms have also been demonstrated to show interesting microlens properties. Diatoms are monocellular, photosynthetic and ubiquitous microalgae, which are contained in a silicified cell wall called frustule, consisting of superimposed layers with intricate and delicate structures (Fig. 9b)^152^. The glass-like structure of frustule endows diatoms as “living photonic crystals”, owing to the alternating refractive index induced by the hierarchically ordered pore structures of silicified frustule^152^. The lens effect of diatoms was firstly demonstrated by Stefano et al.^153^. They observed that the diameter of a light beam was squeezed from 100 to 10 μm at a distance of 104 μm after passing through the frustule of diatom. Tommasi et al. exploited diatom to realize a biological super-lens with sub-diffractive focusing in the far field^154^. The lens-focusing effect of diatom was also proved by others^155–157^. Some unicellular eukaryotes, such as warnowiid dinoflagellates, also contain lens-like organelles, such as hyalosome^158^. Hyalosome serves as a main component of a highly complicated eye-like structure called ocelloid, consisting of distinct constituent parts which are similar to key components of vertebrate ‘camera-type’ eyes. The lens-like components in ocelloids endow unicellular eukaryotes with the ability of light-sensing. This feature reminds us that light can be focused, reflected and refracted when interacting with living cells.

On a higher level of cellular complexity, many mammalian cells also exhibit lensing behavior. Erythrocytes, also known as RBCs, are the major components of blood to transport oxygen in the circulatory system. The morphology of RBCs depends on their pathological states^159^. The mature and healthy RBCs are disc-shaped cells with average diameter of 8 μm and thickness of 2 μm. Due to the absence of nucleus and most organelles, RBCs exhibit homogeneous distribution of refractive index^160,161^. RBCs possess distinctive malleability and deformability in order to pass through narrow blood vessel. From an optical point of view, the intrinsic deformability and the lack of nucleus and organelles make an RBC a sort of disk-shaped microstructured envelope that is exploitable as bio-microlens (Fig. 9c, d)^147,162^. By altering the osmolarity of the ambient buffer, different morphology of RBCs with distinct lens behavior can be obtained. Cells with biconcave disk morphology acted as a divergent lens, with a negative focal distance (virtual focus). Contrarily, spherical RBC behaved as a convergent lens, confining light into a focal spot in the image plane (real focus). When it exhibited as echinocyte, the thorny projection of the cell scattered the light without focusing. These features demonstrated its imaging capability with tunable focal length. Beyond osmotic environments, optically‐induced mechanical stress can also influence the morphology of RBCs, thus regulating their optical lens-like behavior^163^. Furthermore, by exploiting the microlens behavior of RBCs for rotation angles recovery, it is possible to use 3D imaging technology, such as tomographic phase microscopy (TPM), to acquire accurate 3D structure of RBCs in a label-free modality (Fig. 9e)^164^. The malleable and deformable RBCs are ideal candidates as tunable bio-microlenses by adjusting cell morphology in response to microenvironmental changes.

It is worthy to note that the ambient medium dramatically affects the lensing property, since the focusing capability of a living cell completely derives from the difference of refractive index inside and outside the cell. Nilsson *et. al*. demonstrated that the focusing capability of *Synechocystis* was dramatically weakened in water as compared to that exposed in air^165^. This is because the changing from air to water induced a dramatic drop in the difference of refractive index inside and outside cell regions. As to RBCs, the refractive index of RBCs, which is mainly determined by hemoglobin (the major component of dried RBCs mass), increases with the decreased cell volume by changing osmotic conditions^51,139,166^. This indicates the ambient medium should be carefully considered when designing cell-based bio-microlenses.

### Biomedical applications

**Fig. 10:**
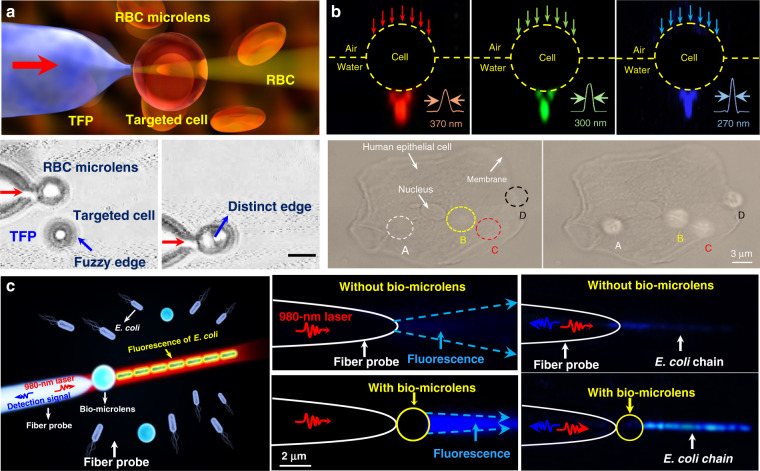
Bio-microlenses for bioimaging. **a** Schematic illustration and optical microscopic images of RBC-based microlens for single cell membrane imaging^167^. Copyright 2019, American Chemical Society. **b** Single-cell biomagnifier for optical nanoscopes^168^. Copyright 2019, Springer Nature. **c** Living yeast cell microlens for fluorescence enhancement^170^. Copyright 2017, American Chemical Society

Owing to the inherent advantages of excellent biocompatibility, cell-based bio-microlenses are attracting considerable attention in biological applications. For example, biological cells have been applied for label-free imaging of living cells or other nanostructures. Liu et al. presented an RBC microlens assembled on a tapered fiber probe for scanning imaging of cell membrane in three dimensions (Fig. 10a)^167^. Besides, the stretch of the cell membrane was achieved by RBC-based microlens in a noncontact and noninvasive manner. In a similar way, Li et al. generated a biomagnifier for subdiffraction-limit imaging by trapping a single yeast cell at the tip of an optical fiber^168^. This biomagnifier was capable to operate at different laser wavelengths in the visible region, and exhibited the capability for nanostructure imaging in a scanning manner. With the help of the biomagnifier, subcellular structures in a human epithelial cell were clearly resolved without the need to label cells with specific fluorescent molecules (Fig. 10b). The fibrous cytoskeleton and bilayer membrane of cells were clearly illustrated with the help of biomagnifiers, which were unobtainable by the traditional microscope. The most striking advantage of cell-based biomagnifier over conventional optical microscopes is that the imaging can be conducted in a contact mode, so the possible evanescent-propagation coupling may contribute to super-resolution imaging^169^. This cell-based bio-microlens were also exploited for fluorescence enhancement. In their scenario, living yeast cell or human cell were optically trapped by a tapered fiber, acting as a microlens to confine excitation light to a sub-wavelength region (Fig. 10c)^170^. The confined light facilitated fluorescently labeled single-cell imaging and real-time detection of bacteria.

**Fig. 11:**
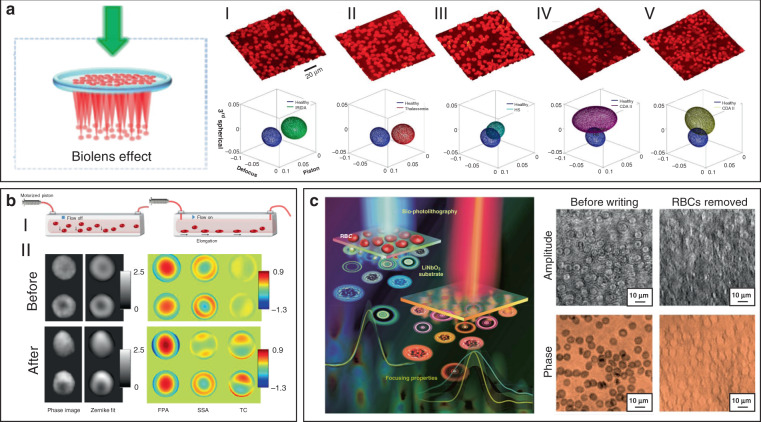
RBC microlenses for blood diagnosis and bio-printing. **a** Label-free optical marker for blood diagnosis with RBC as a cell-based bio-microlens. 3D visualization of QPM reconstructions and blood-disorder identification based on WZF: (I) heathy RBCs and (II) IRIDA, (III) thalassemia, (IV) CDA I, and (V) CDA II subjects^172^. Copyright 2018, American Chemical Society. **b** RBC lens behavior as a biomechanical marker to monitor the deformations of the RBCs^174^. Copyright 2019, Frontiers. **c** RBCs serve as biophotomasks for writing laser spots into ferroelectric crystals^175^. Copyright 2019, American Chemical Society

Since morphological abnormalities of RBC are closely coupled with blood-related diseases, precise morphologic analysis of RBCs can facilitate blood diagnostics. In general, healthy RBCs exhibit typical disk-like morphology, while pathological ones possess significantly different shapes. RBCs with biolens property can be exploited as a noninvasive, label-free, and fast screening tool to identify abnormal RBCs from healthy cases. Quantitative phase imaging (QPI) is an interferometric imaging technique, that uses reflective index as an inherent optical contrast to quantitatively monitor living cells and tissues with high accuracy^171^. For example, Lee et al. demonstrated a quantitative phase imaging (QPI) technique for the differential diagnosis of RBC membrane defects^172^. By using the adaptive microlensing behavior of RBC array, reconstruction of the 3D morphologies of individual living RBC samples can be achieved by QPI (Fig. 11a)^172^. Further, abnormal cells from patients with different types of anemia were able to be identified from normal healthy ones. Alternatively, label-free sensing and identification of pathological RBCs based on biolens property were achieved with the help of other imaging technologies, such as digital in-line holographic microscopy (DIHM)^173^. In another study, the lensing behavior of RBCs was demonstrated to serve as a new biomechanical marker to monitor the biomechanical deformations of the RBCs when hydrodynamic stress occurred. (Fig. 11b)^174^. Cellular deformations were induced mechanically by accurate manipulation of shear stress in microfluidic streams (Fig. 11bI). With the help of light-focusing effect of RBCs with different phenotypes, the biomechanics of the RBC was evaluated through a numerical analysis (Fig. 11bII). This proposed biomarker possessed the potential to identify the phenotyping of anemia samples.

More recently, Miccio et al. extended the cell-based bio-microlens application of RBCs to microstructure fabrication^175^. In their scenario, RBCs were served as microlenses for bio-photolithography to transfer phase modulation into a photoactivated solid surface (Fig. 11c). Taking advantage of the different lens behavior, the different morphologies of RBCs were clearly imprinted and distinguished on the substrate. The possibility to print the optical fingerprint of the RBCs into a solid material will find new applications on both disease diagnosis and cell/material interfacing.

### Limitations and potential improvements

Due to the high biocompatibility, high sensitivity, and miniaturization, cell-based bio-microlenses have provided promising potentials for different biomedical applications, such as single-cell analysis, endoscopic vision, disease diagnosis, bioimaging and biosensing. Despite the above advances, the exploration of cell-based bio-microlenses is still at infancy stage with many issues to be resolved. For example, the manipulation of bio-microlenses formed by RBCs are dramatically limited to the long response time (~10 s), because of the delayed response of cells to the variation of osmotic pressure of environmental medium^152^. Another specific problem is that the aforementioned tunable optical properties are limited to immersion environments as cells are typically operated in liquids. Additionally, precise control of cell-based bio-microlenses is challenging, since the optical properties of biological cells can be influenced by diverse fundamental factors.

## Conclusion and outlook

The goal of this review is to show the intriguing progresses of emerging biophotonic probes made from biological entities, including virus, bacteria, cells and tissues, for bio-detection and imaging. We reviewed three different biophotonic probes, i.e., biolasers, biophotonic waveguides and bio-microlenses, with the optical functions from light generation, to light transportation and light modulation. We focused on biophotonic probes that open up an entirely new window for biophotonic researches and also for biomedical applications, e.g., bio-lasers for bio-detection, cell tagging and tissue imaging, biophotonic waveguides based on living cells for optical detection and sensing, and bio-microlenses for single-cell imaging and blood diagnostics. Compared with conventional photonic components, these biophotonic probes exhibit some remarkable advantages. First, they offer inherent and favorable opportunities for biocompatibility and biodegradability in comparison to traditional synthetic materials. Additionally, the development of biophotonic probes using biological cells/tissues let these biological entities serve simultaneously as optical components and testing samples, which facilitate in vivo and real-time sensing, detection, and imaging.

Nevertheless, biophotonic probes made from these biological entities still suffer from variability and limited designability. Despite the significant progress already achieved, the overall development of biophotonic probes is still in the infancy and there is still much to be explored. Firstly, more efforts are still needed to fully understand and to discover the broad and diverse family of living organisms that are suitable to serve as photonic probes. Besides, so far, most concepts and techniques have been demonstrated by in vitro or animal studies as proof on concept. Much future work is necessary to prove the feasibility in preclinical and clinical practical applications. For biolasers, more attention should be paid on in vivo applications. In this case, in addition to the detection via the laser emission with high selectivity, the localized high energy of the laser emission will also be very helpful for in situ therapy. On the other hand, laser emission in biofluids from clinical samples will provide many possibilities for disease diagnosis in real time. For biophotonic waveguides, more efforts are needed for the formation of robust biophotonic waveguides that are capable for long-range light delivery for noninvasive medical diagnosis in deep-tissue region. The integration of a biophotonic probe that are capable to carry out biomedical tasks with an optical readout with high sensitivity and selectivity is very useful for real-time diagnosis. Recent developments in smartphone technology have enabled portable and highly sensitive molecular diagnosis on a smartphone-based platform. In this regard, bio-microlenses integrated into a smartphone-based platform has great potential in optical imaging and blood diagnosis with clinical samples in a portable way in real time, which is of great importance in resource-limited regions.

## Supplementary information

Copyright permission files
