# Supplementary material for: Biophotonic probes for bio-detection and imaging
Source: Light Sci Appl. 2021 Jun 9;10:124. doi: 10.1038/s41377-021-00561-2 (PMC8190087; doi:10.1038/s41377-021-00561-2)
Supplement: Supplementary file 1 — Copyright permission files [file 41377_2021_561_MOESM1_ESM.pptx]

## Slide 1
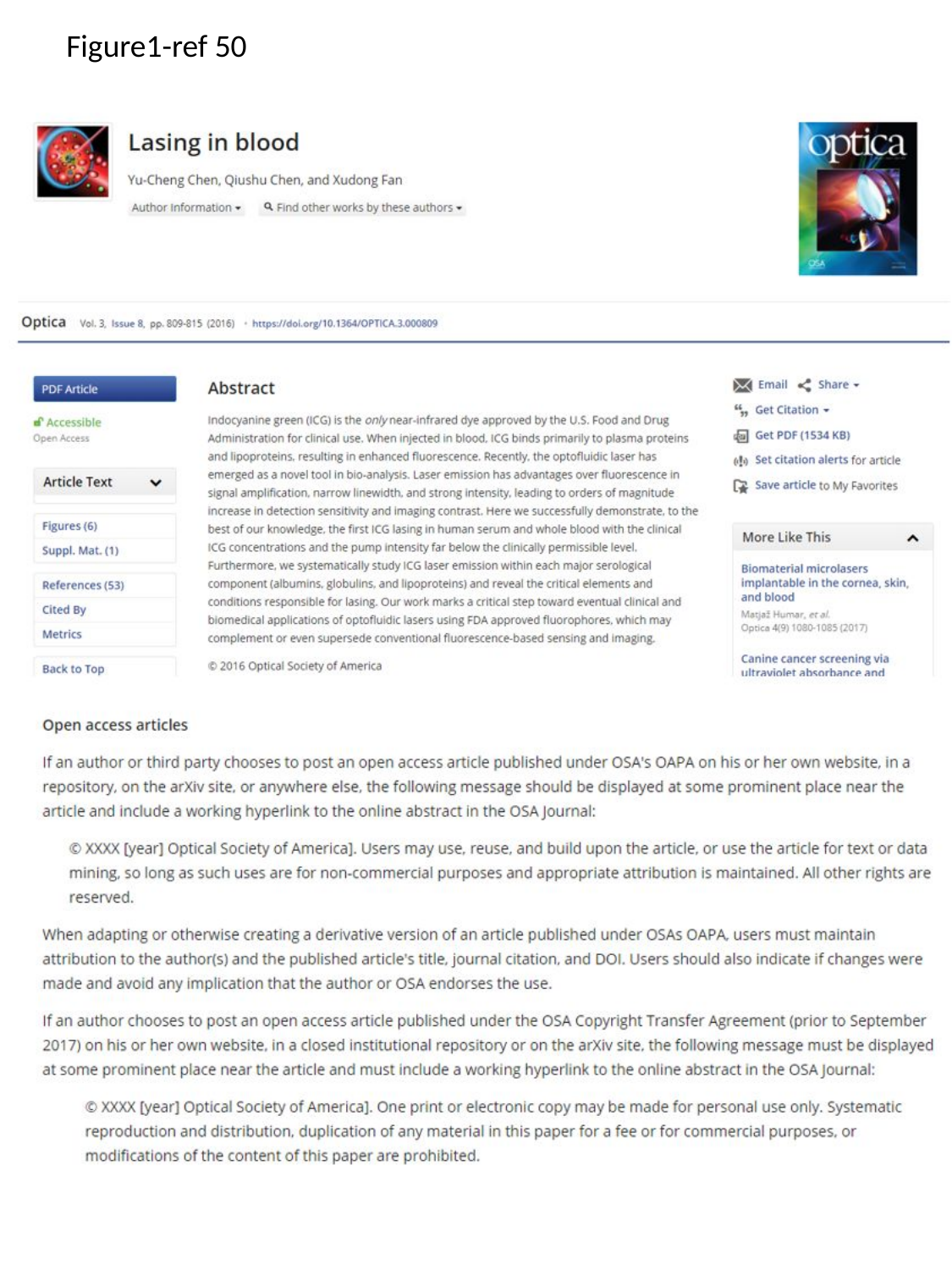

Figure1-ref 50

## Slide 2
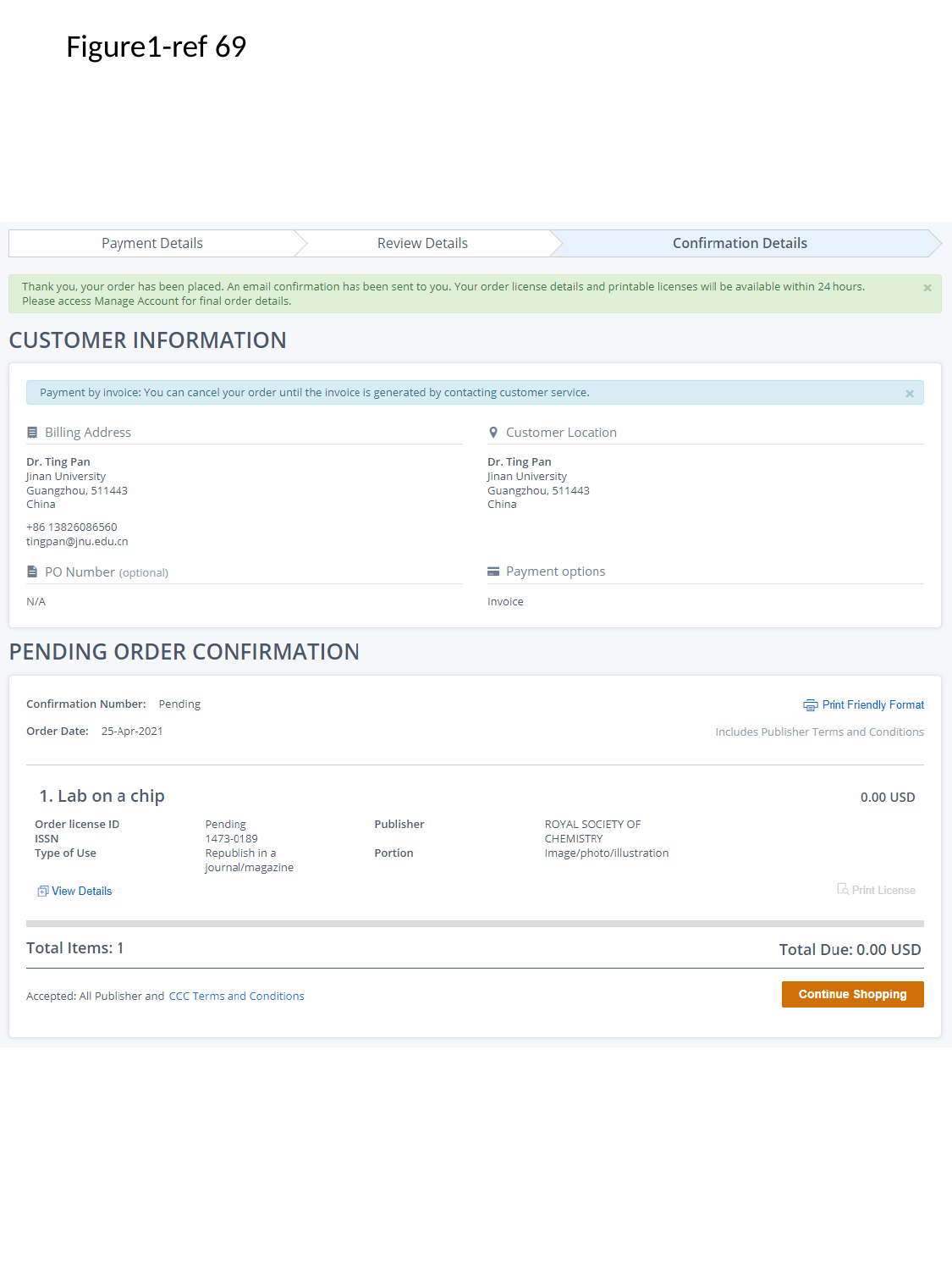

Figure1-ref 69

## Slide 3
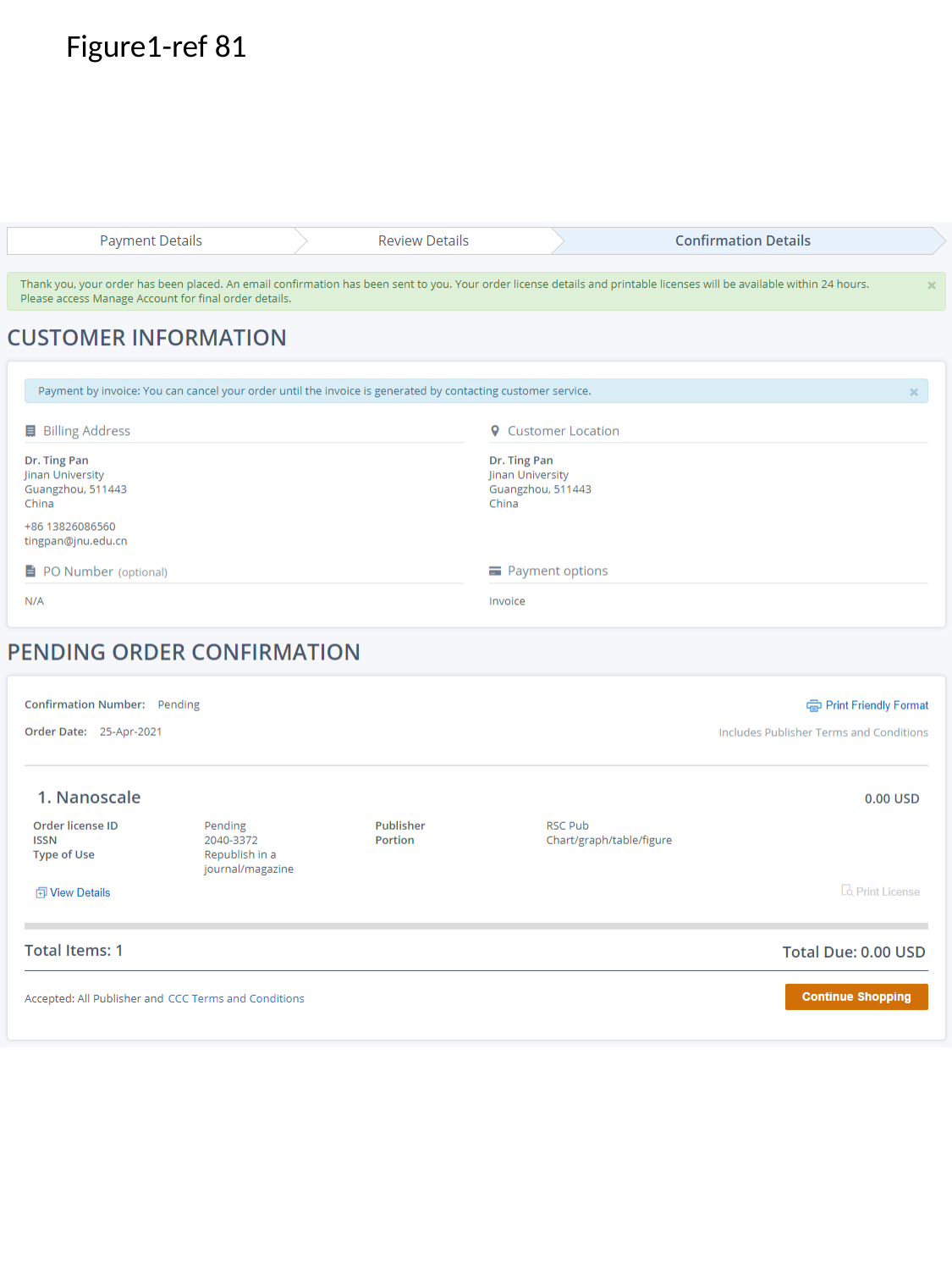

Figure1-ref 81

## Slide 4
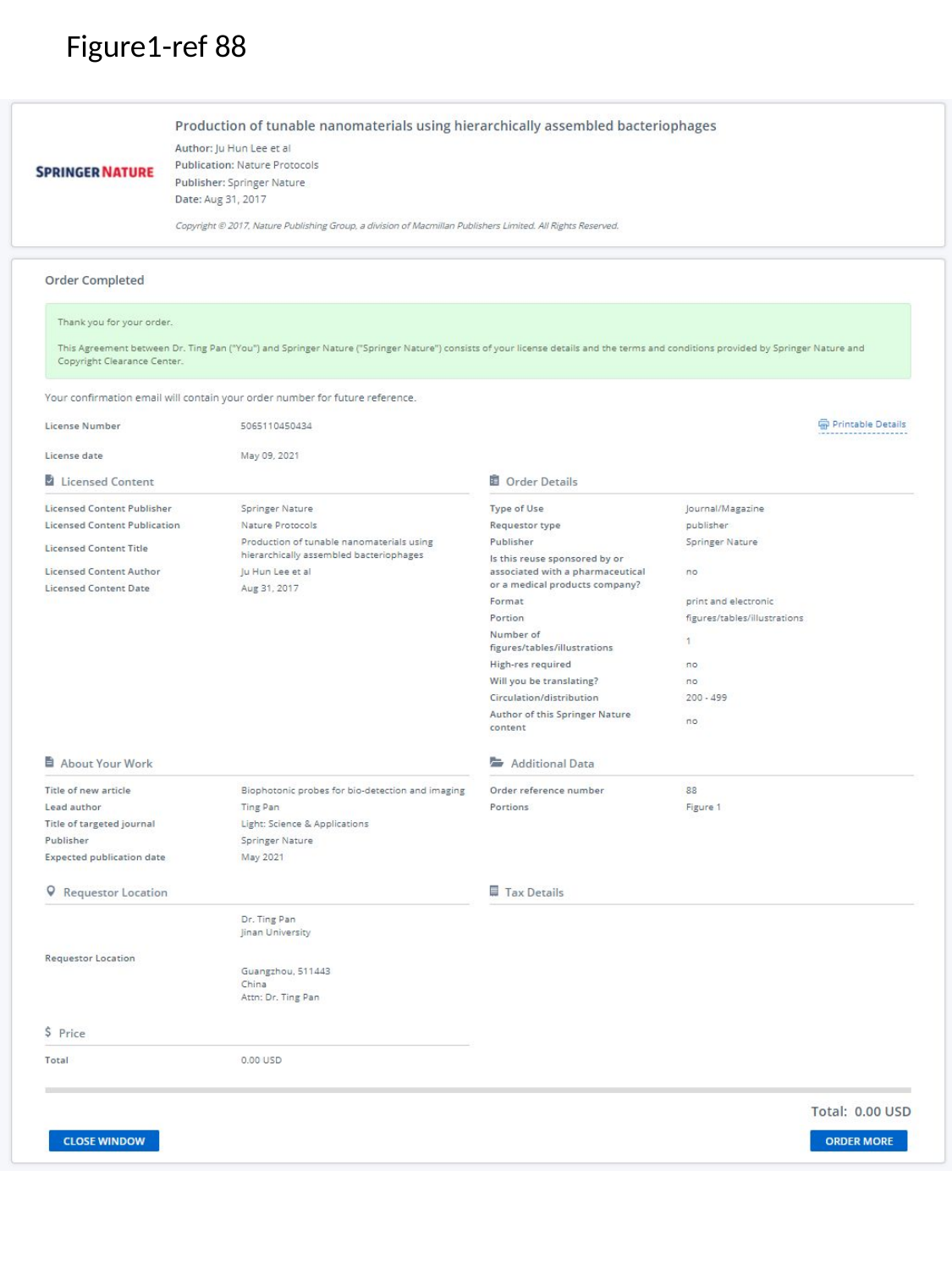

Figure1-ref 88

## Slide 5
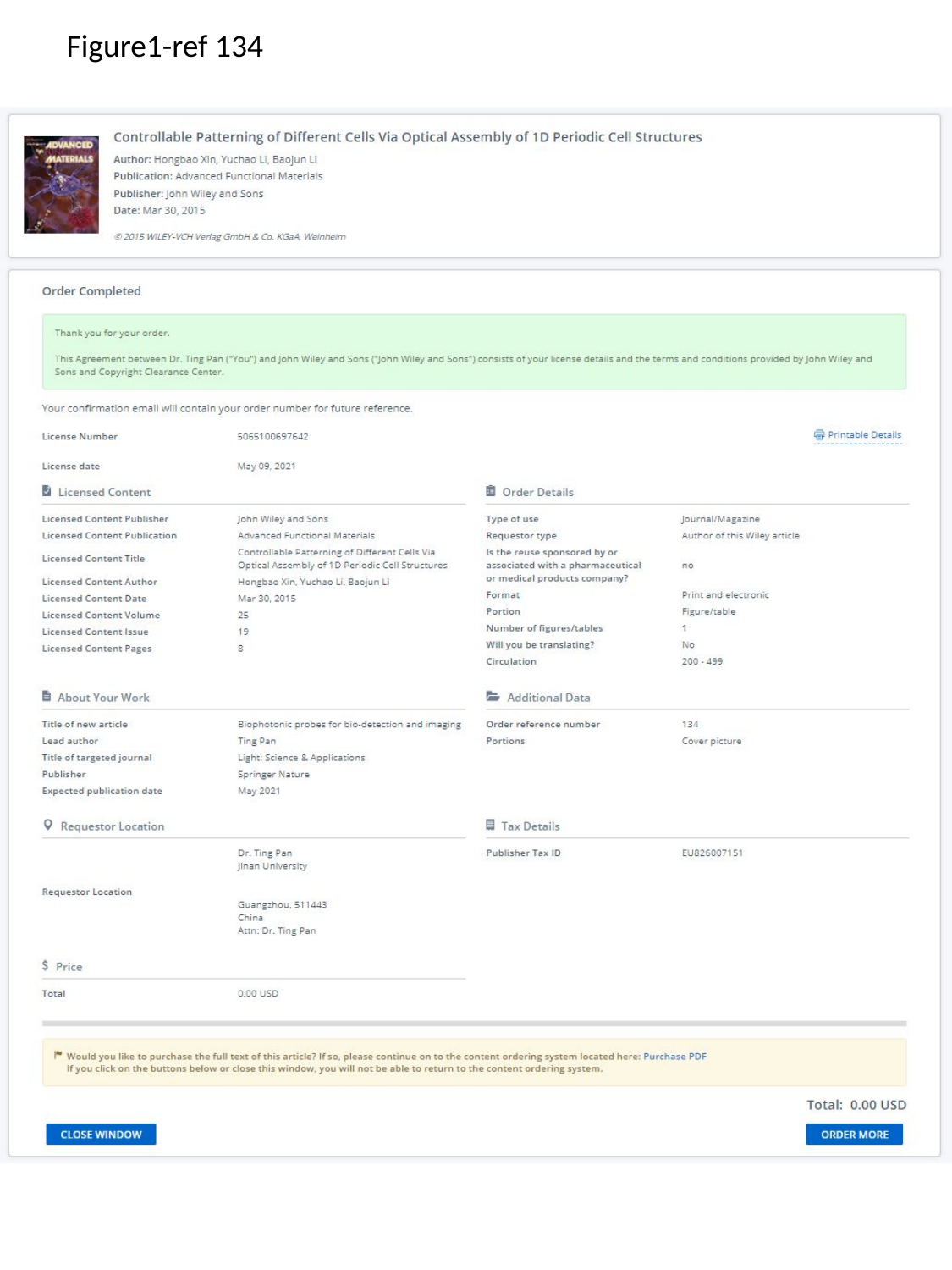

Figure1-ref 134

## Slide 6
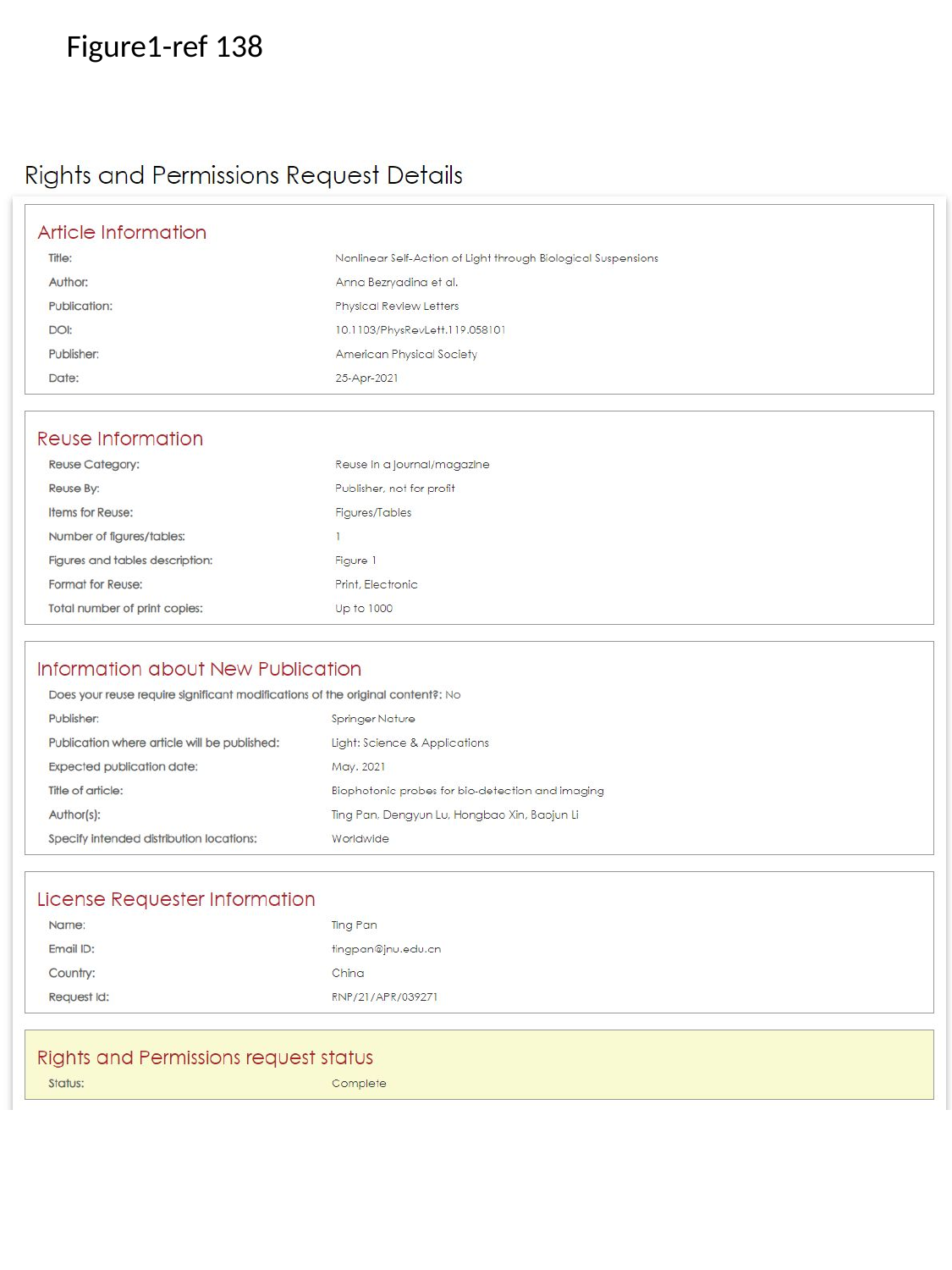

Figure1-ref 138

## Slide 7
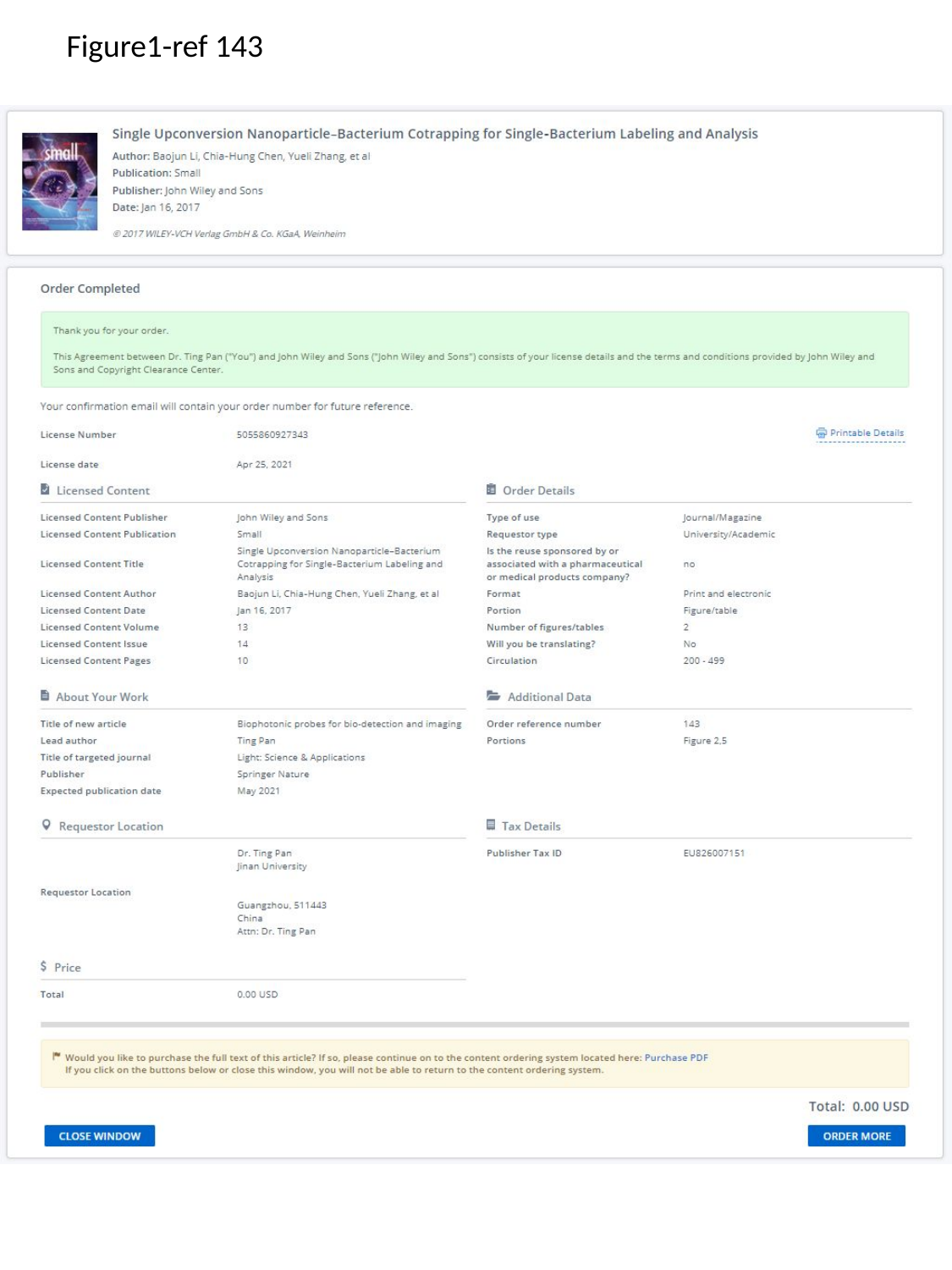

Figure1-ref 143

## Slide 8
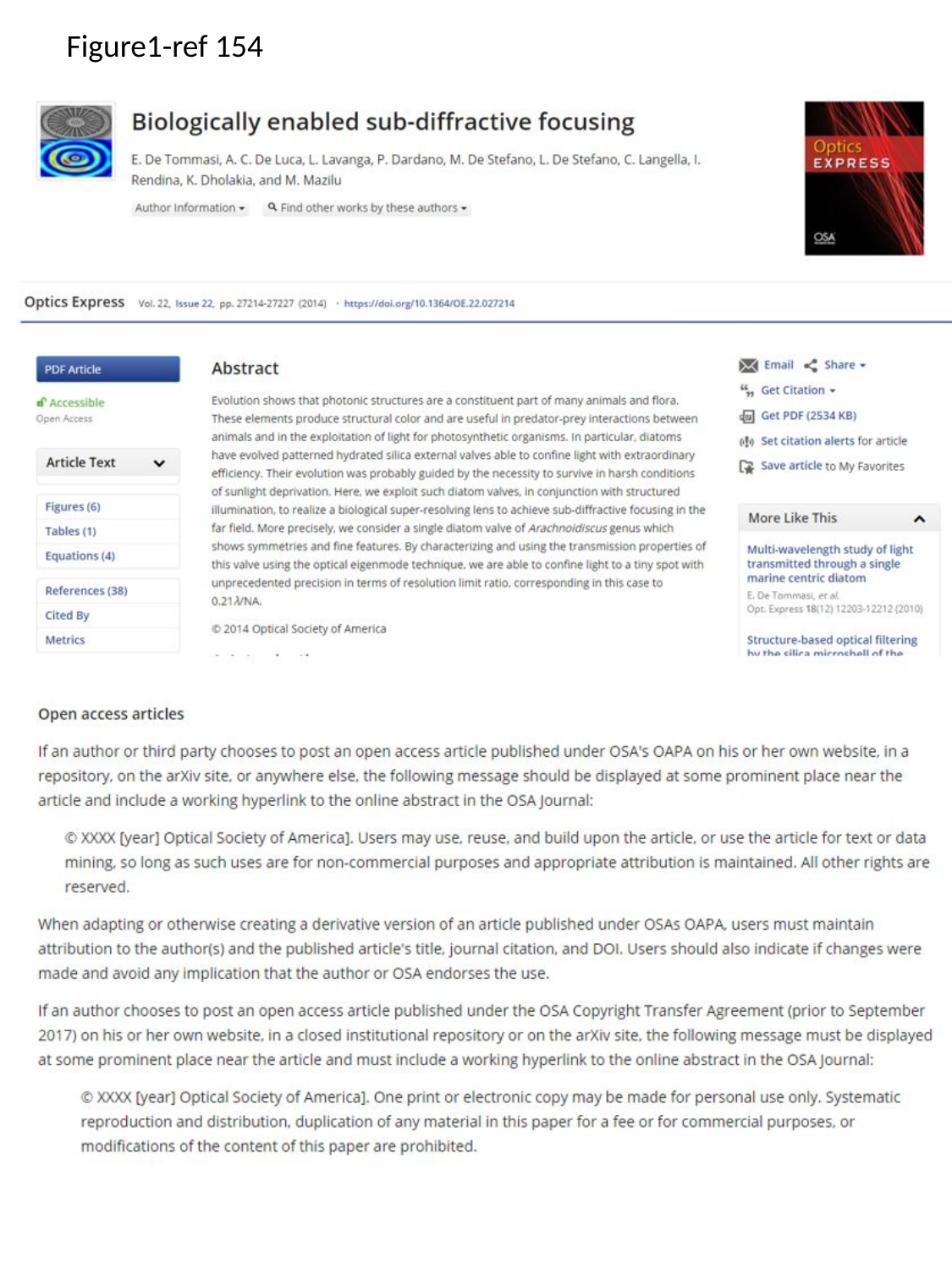

Figure1-ref 154

## Slide 9
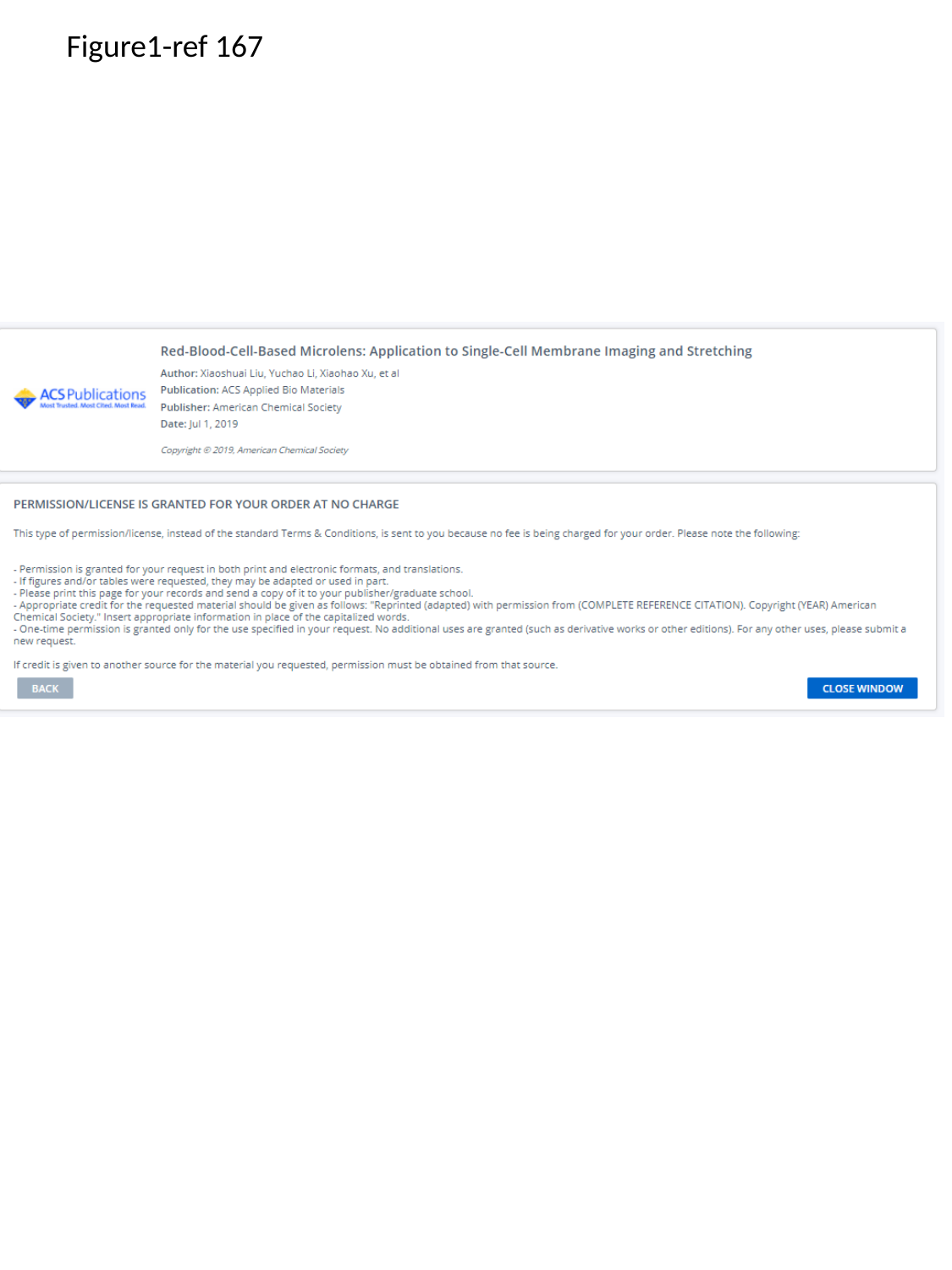

Figure1-ref 167

## Slide 10
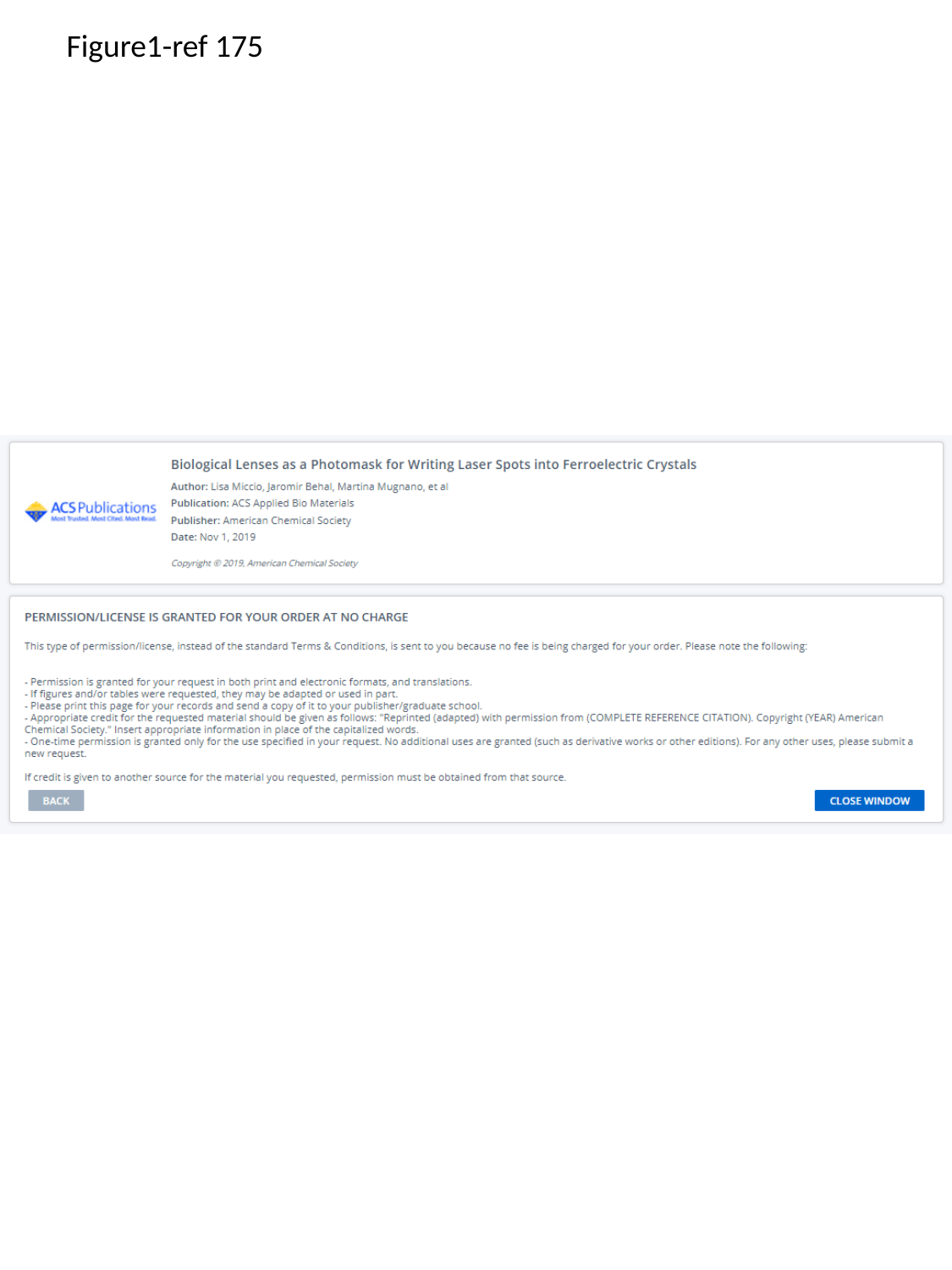

Figure1-ref 175

## Slide 11
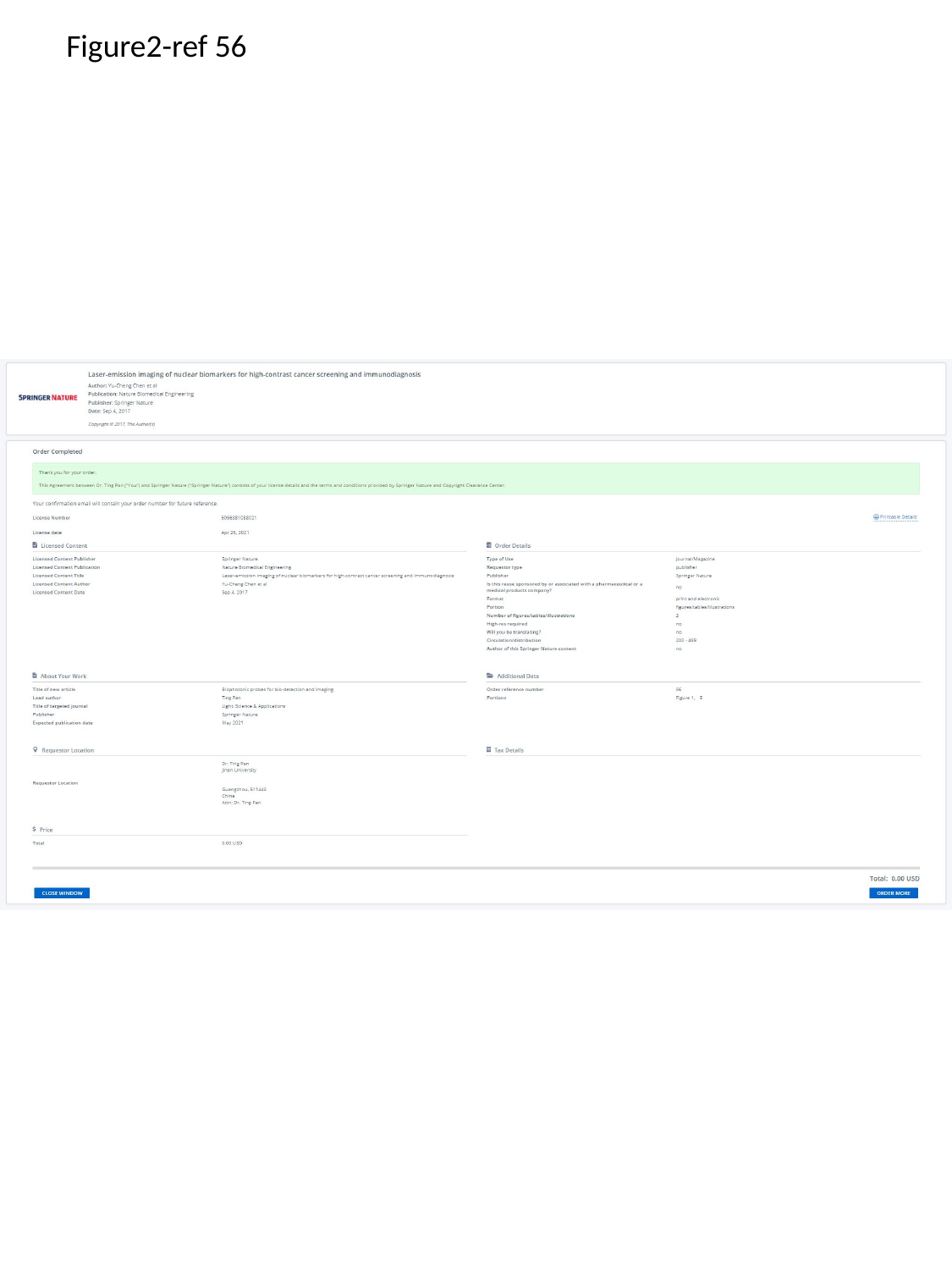

Figure2-ref 56

## Slide 12
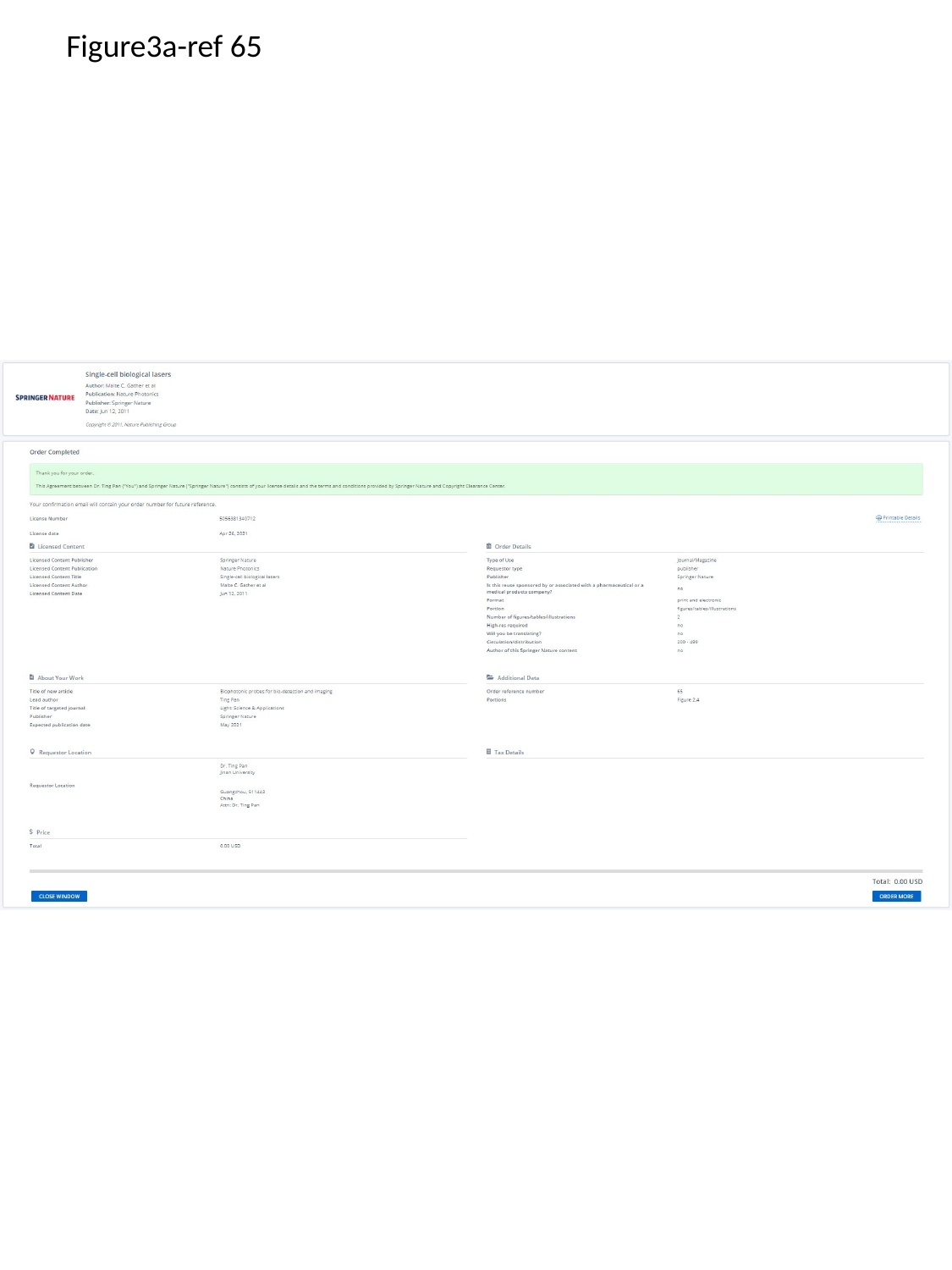

Figure3a-ref 65

## Slide 13
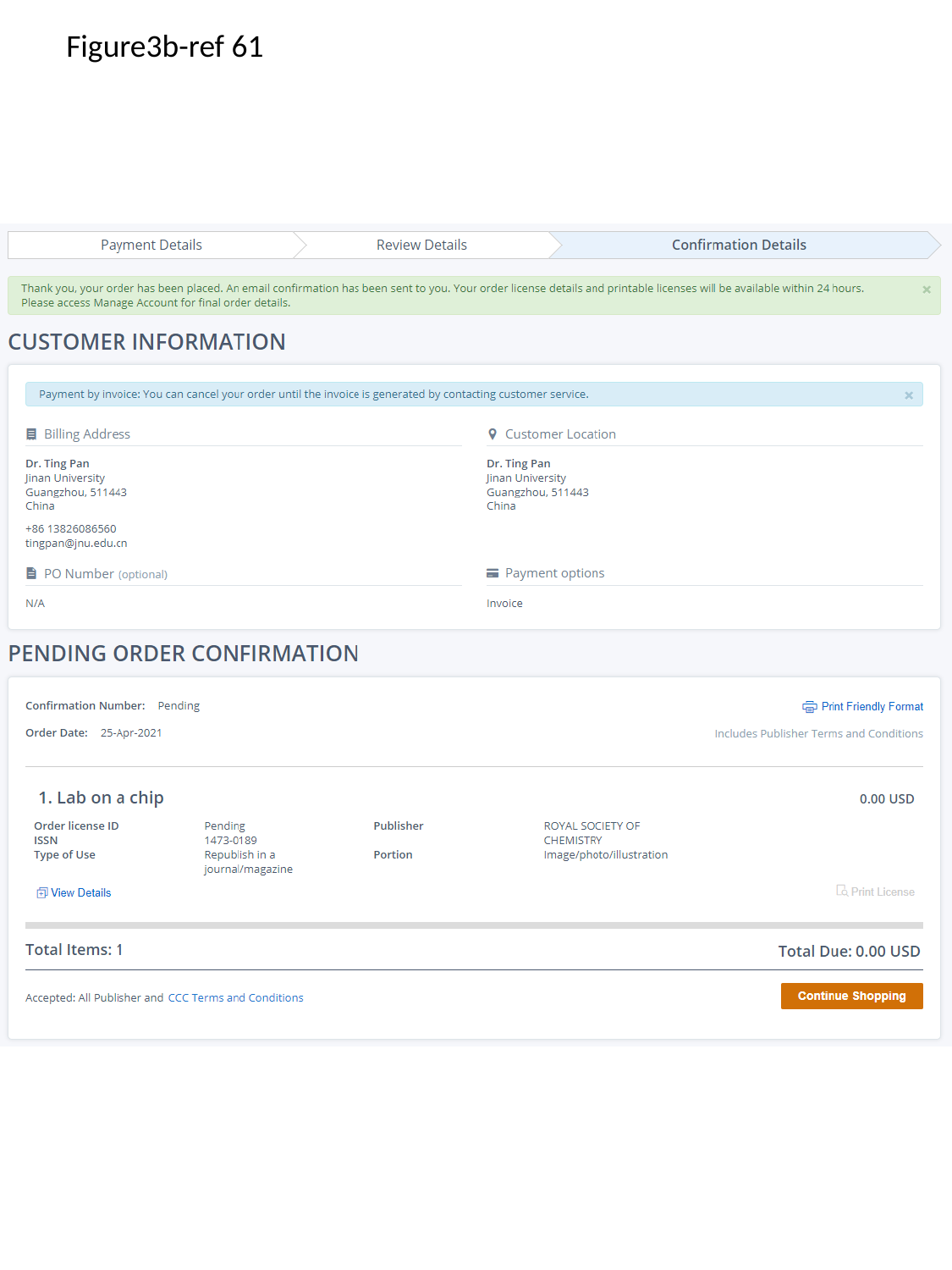

Figure3b-ref 61

## Slide 14
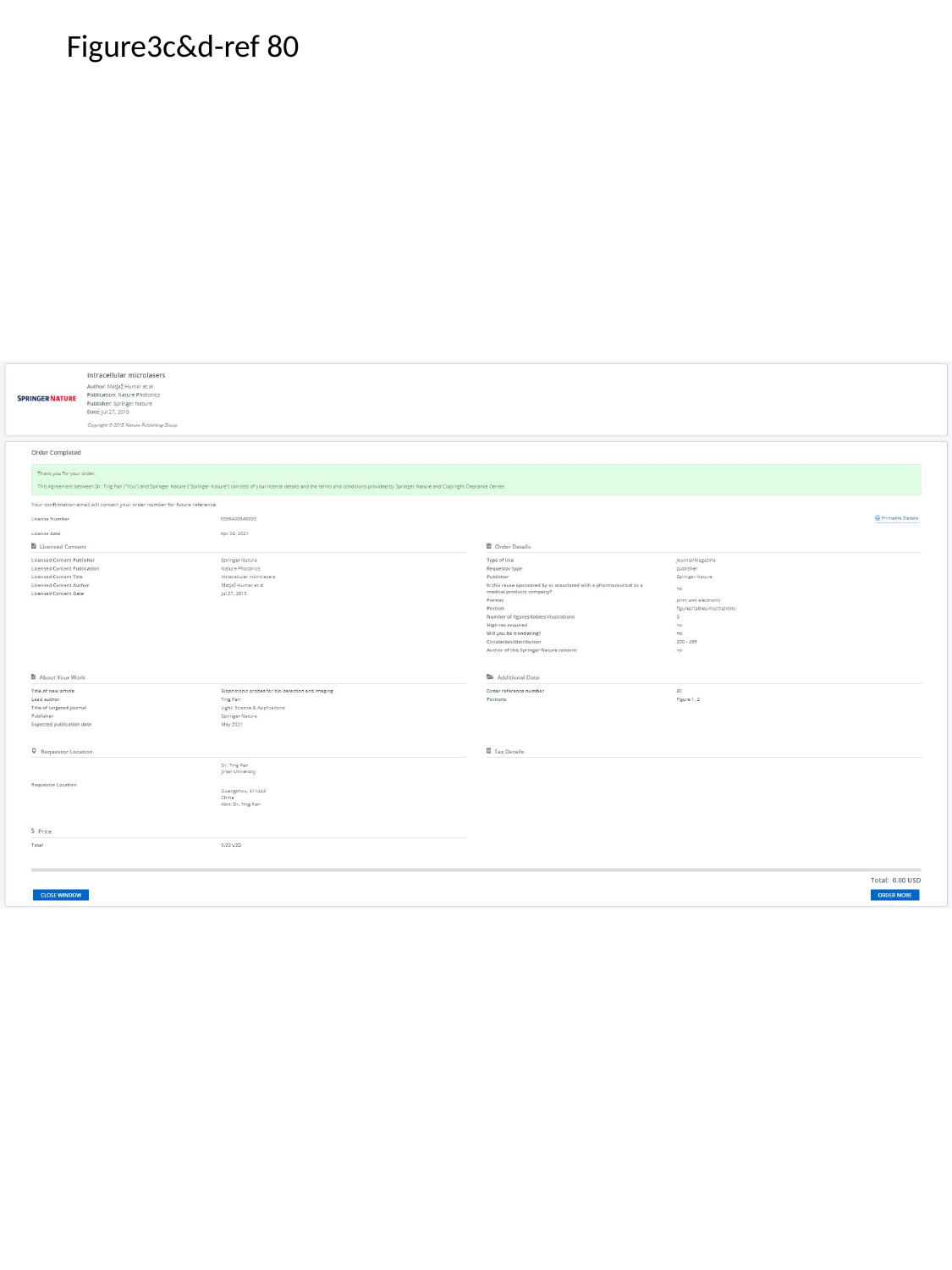

Figure3c&d-ref 80

## Slide 15
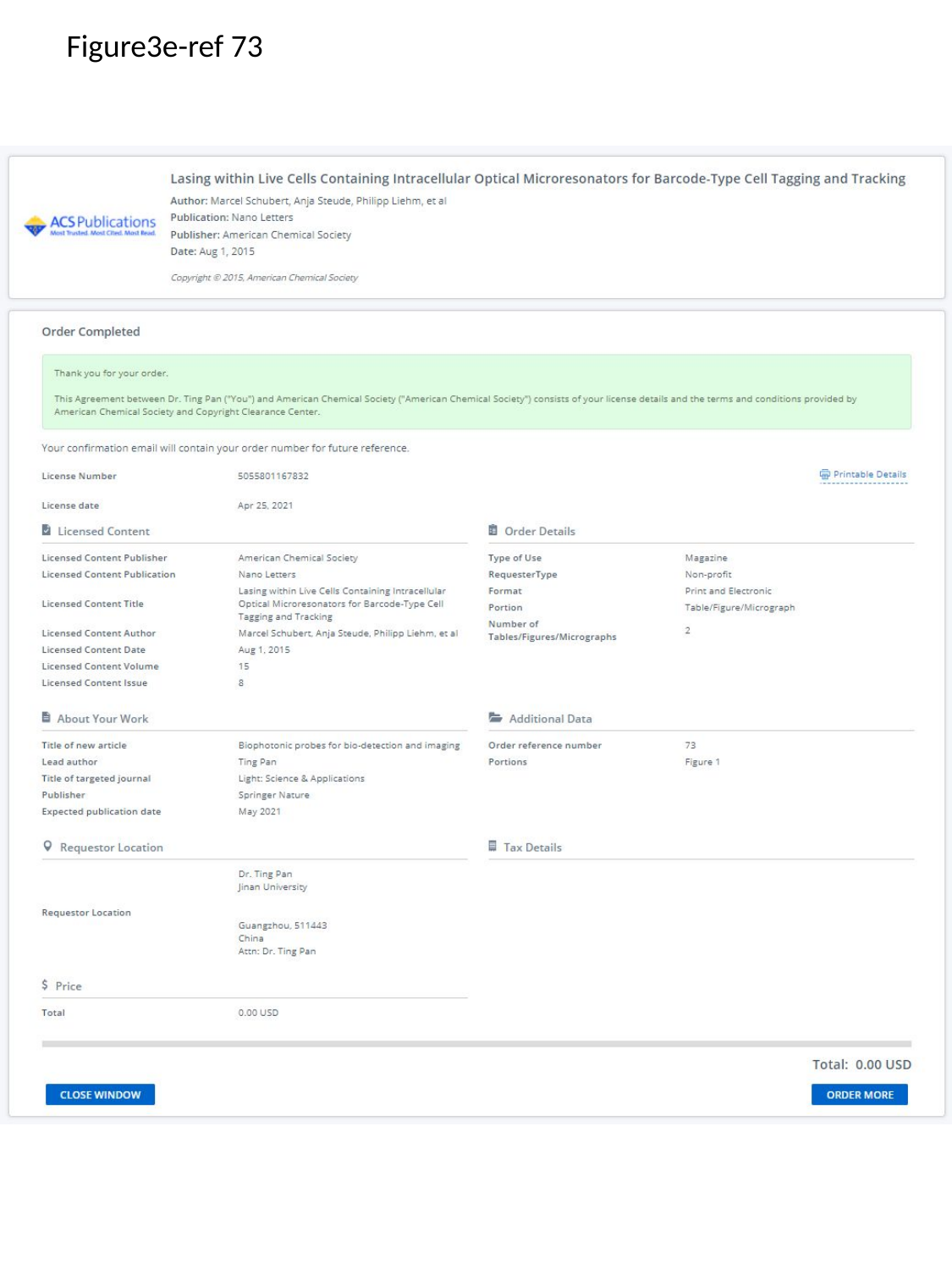

Figure3e-ref 73

## Slide 16
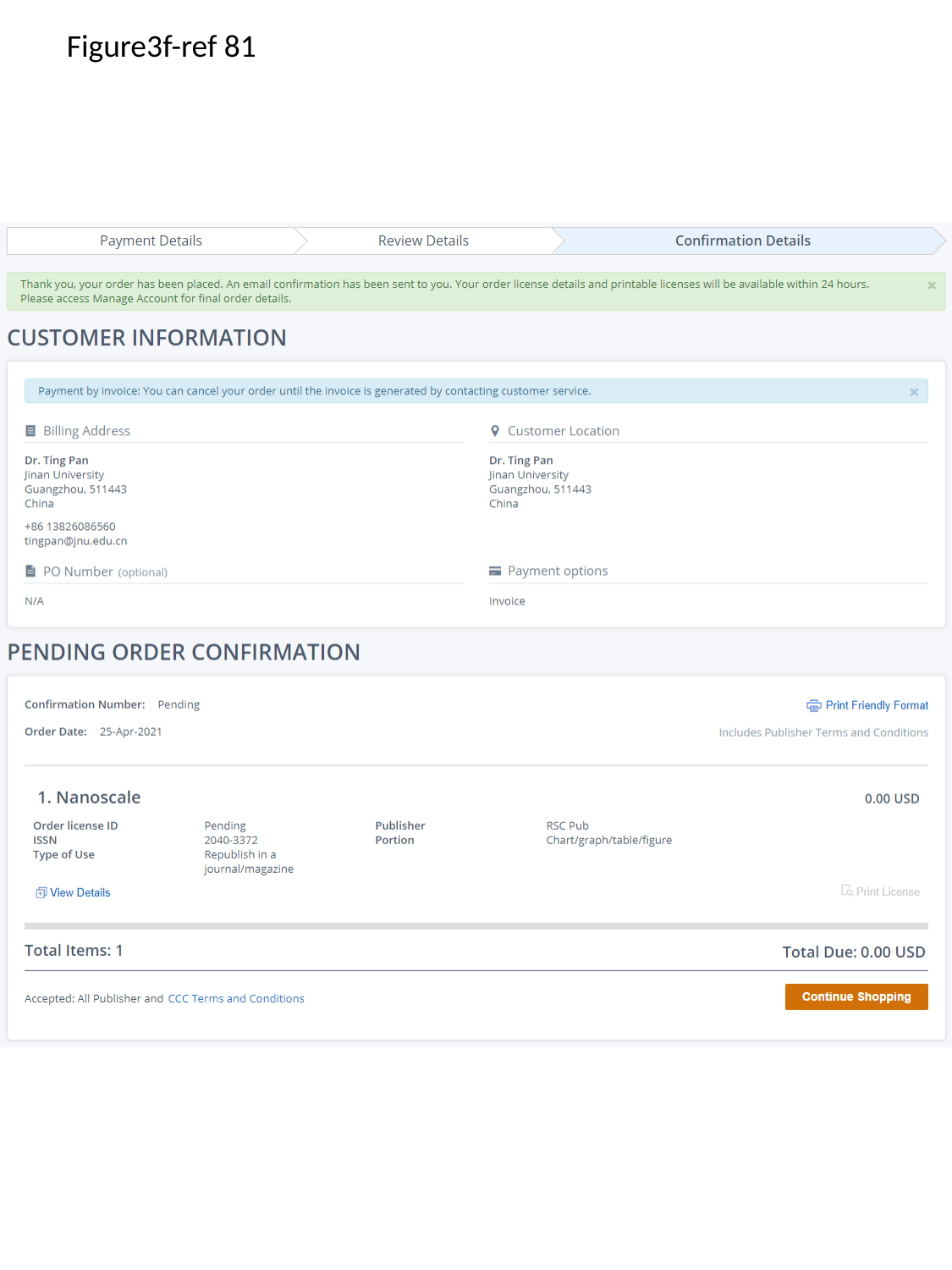

Figure3f-ref 81

## Slide 17
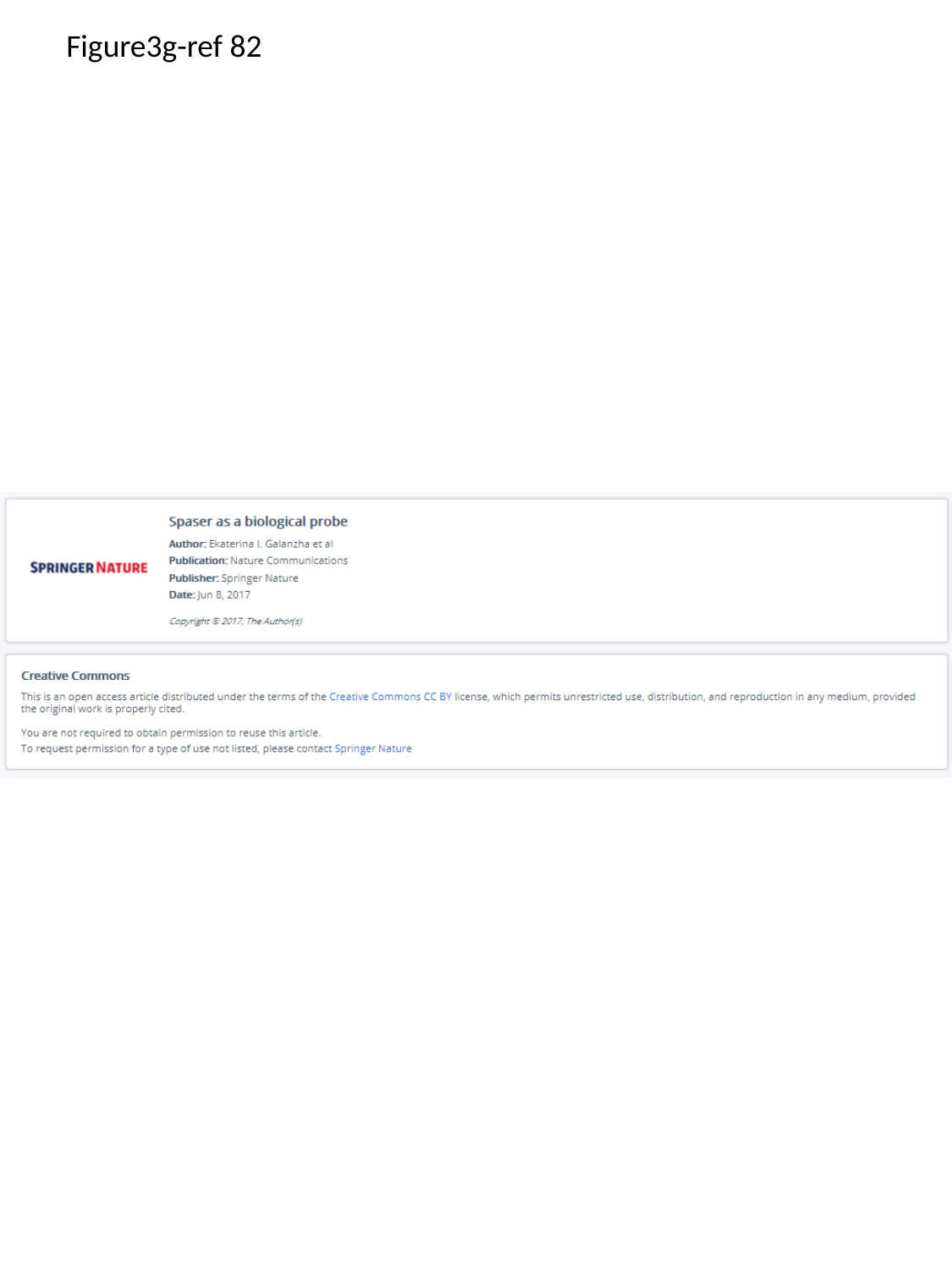

Figure3g-ref 82

## Slide 18
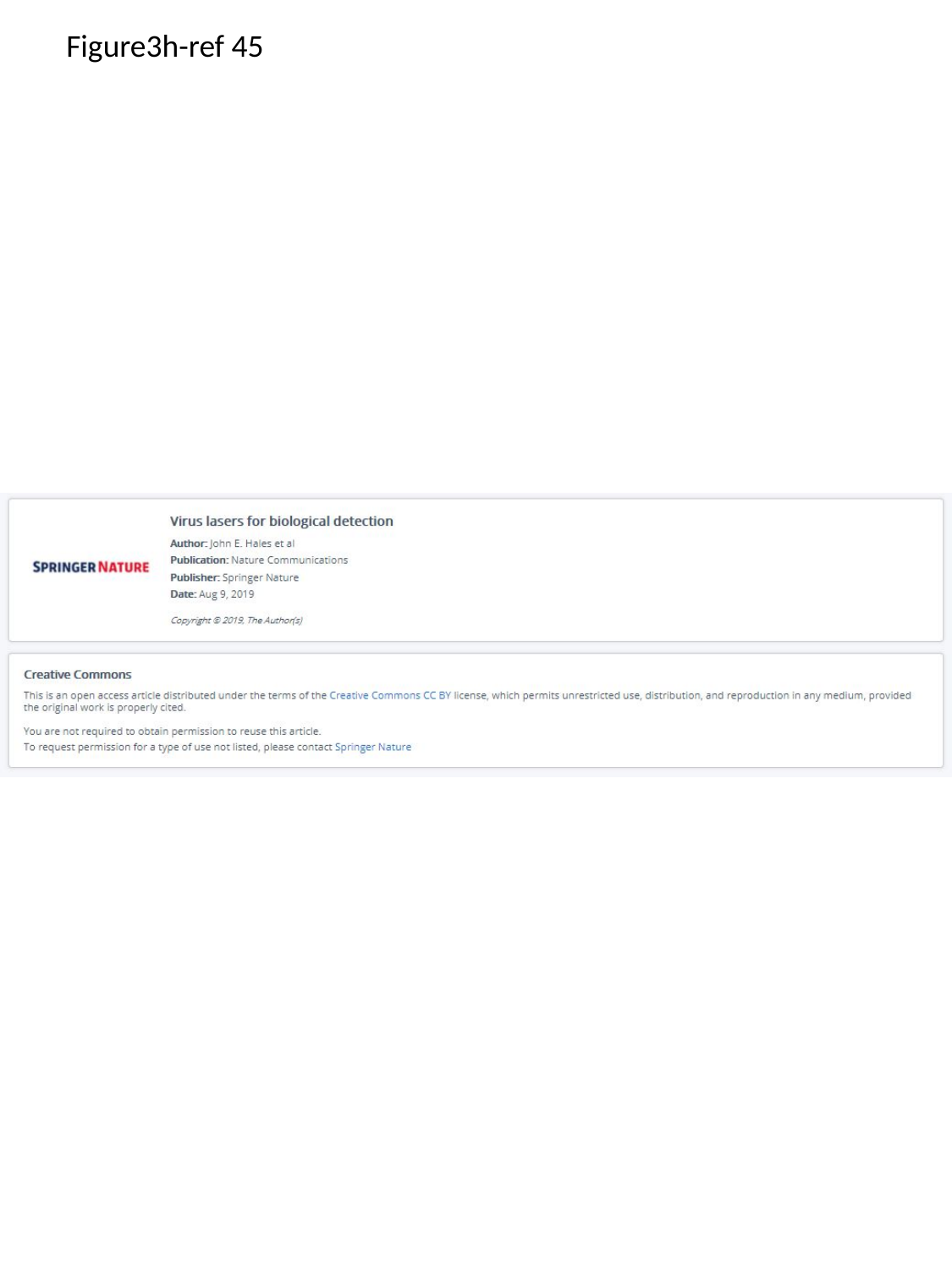

Figure3h-ref 45

## Slide 19
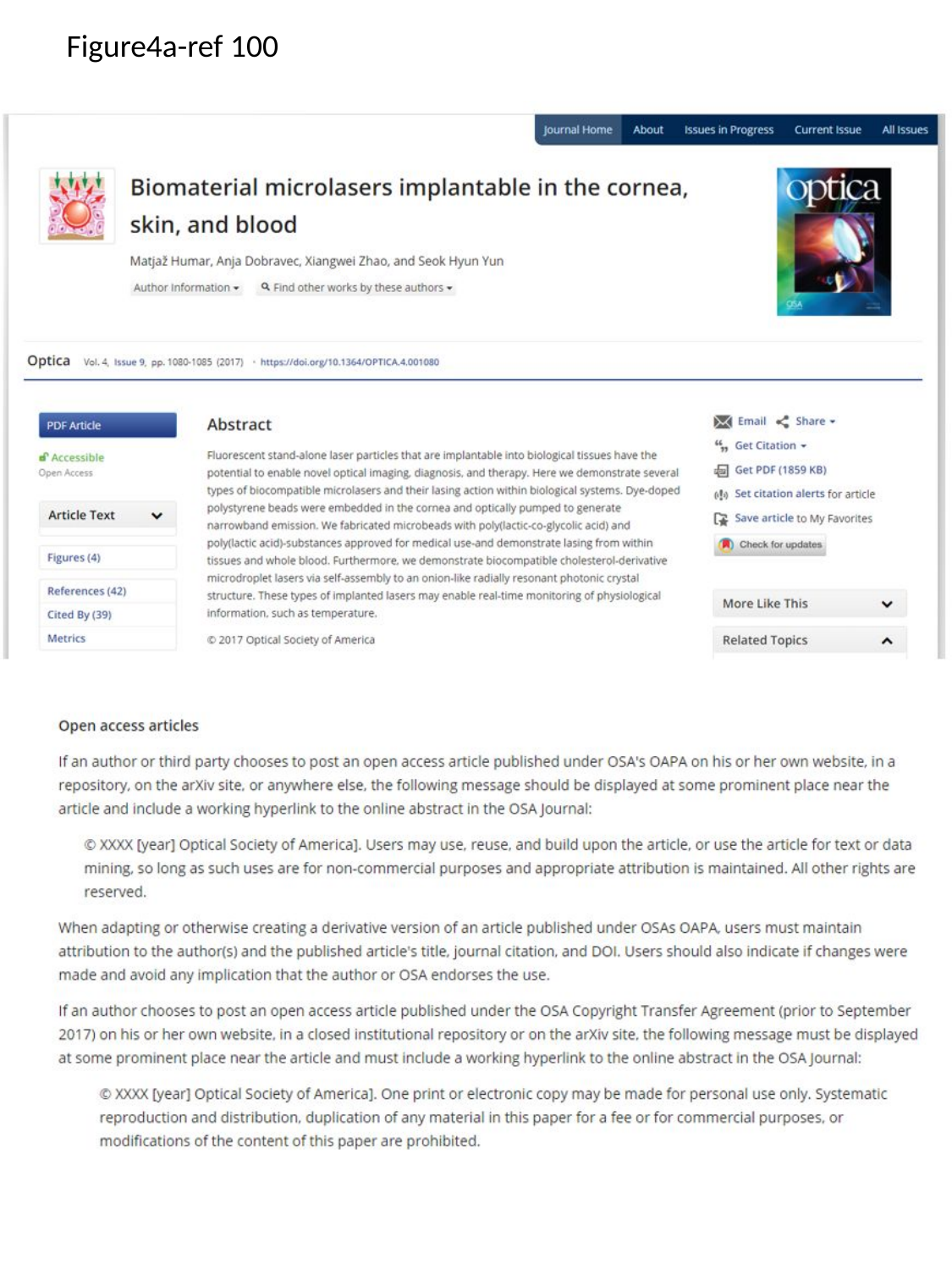

Figure4a-ref 100

## Slide 20
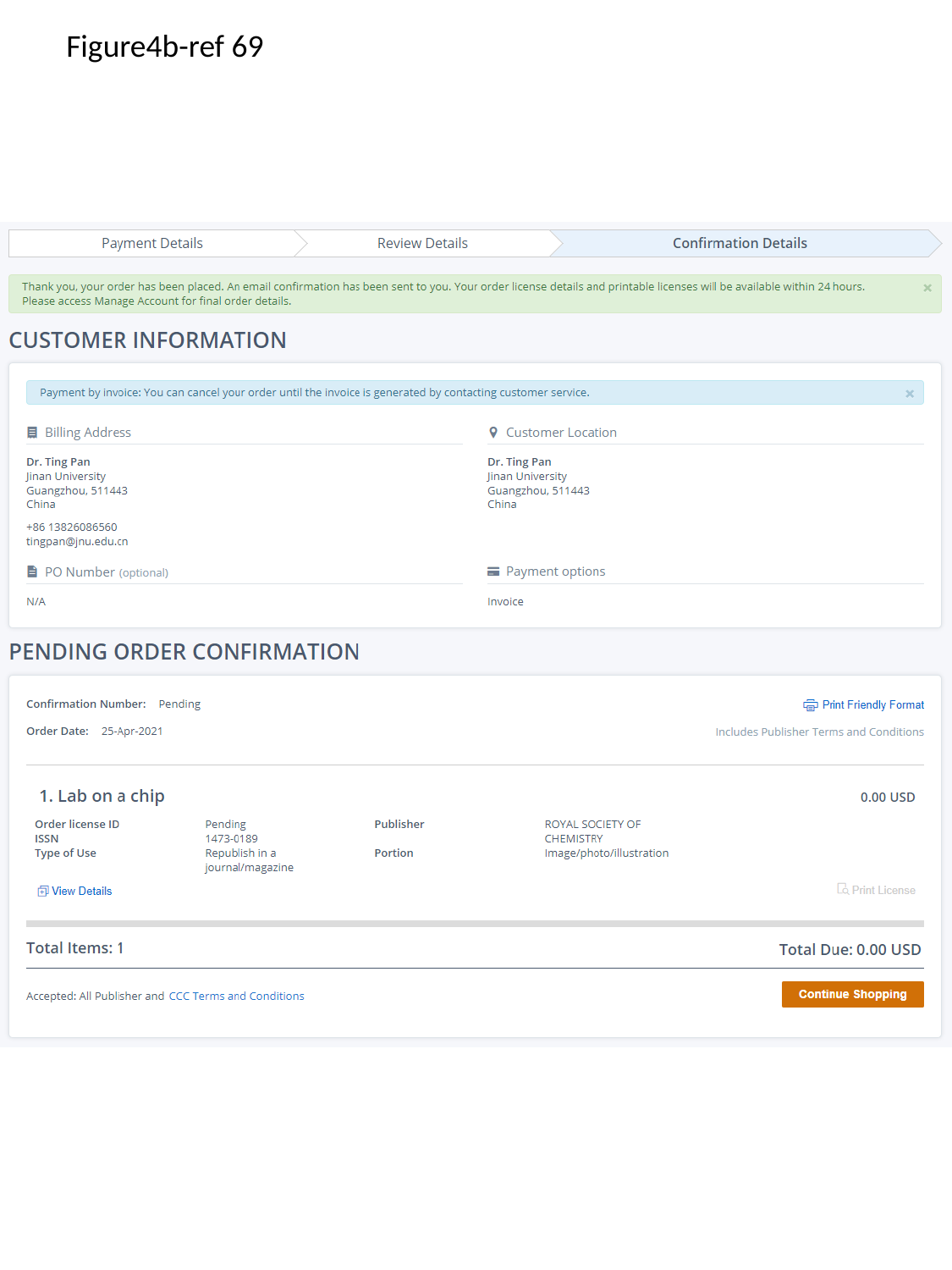

Figure4b-ref 69

## Slide 21
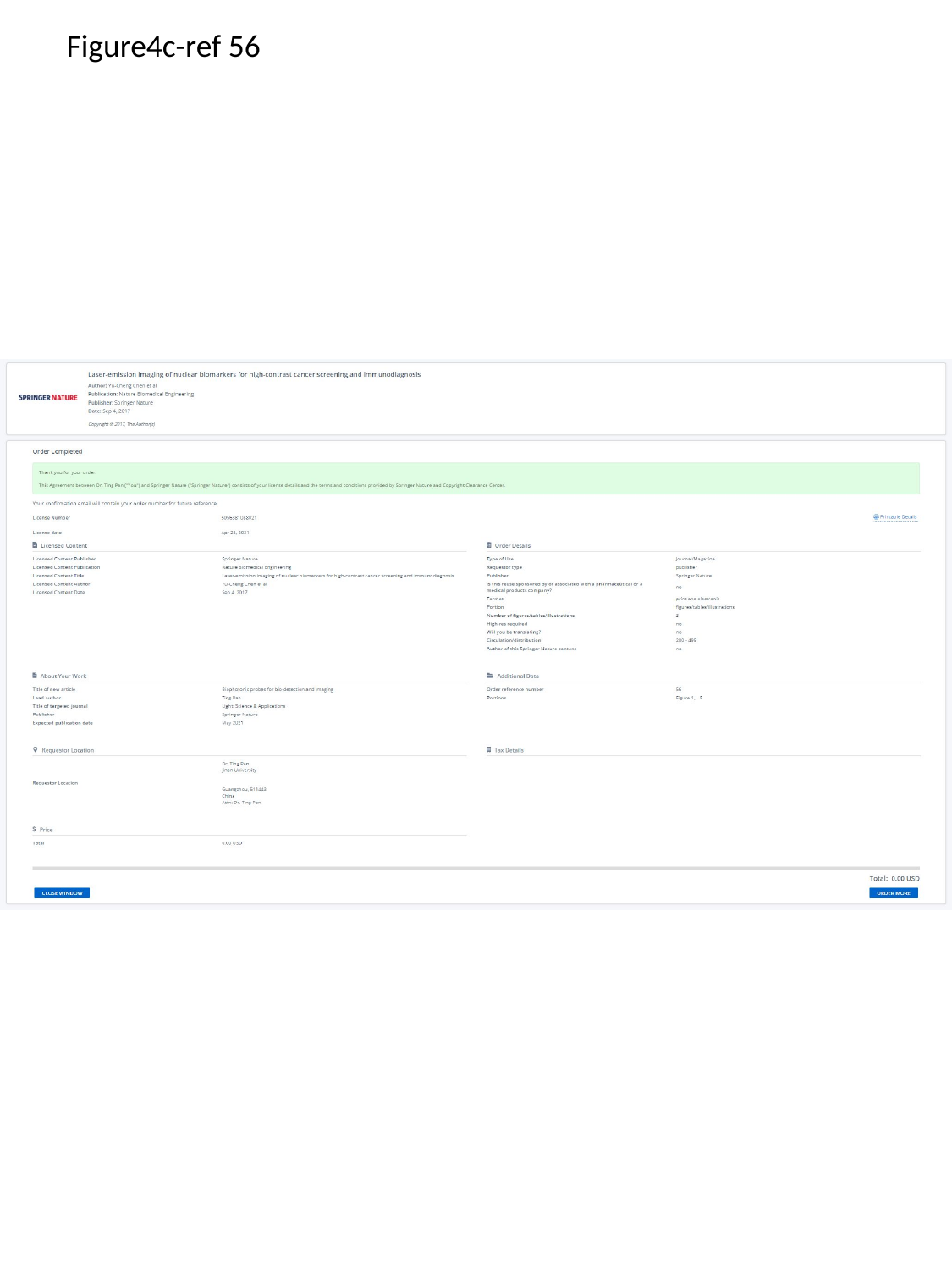

Figure4c-ref 56

## Slide 22
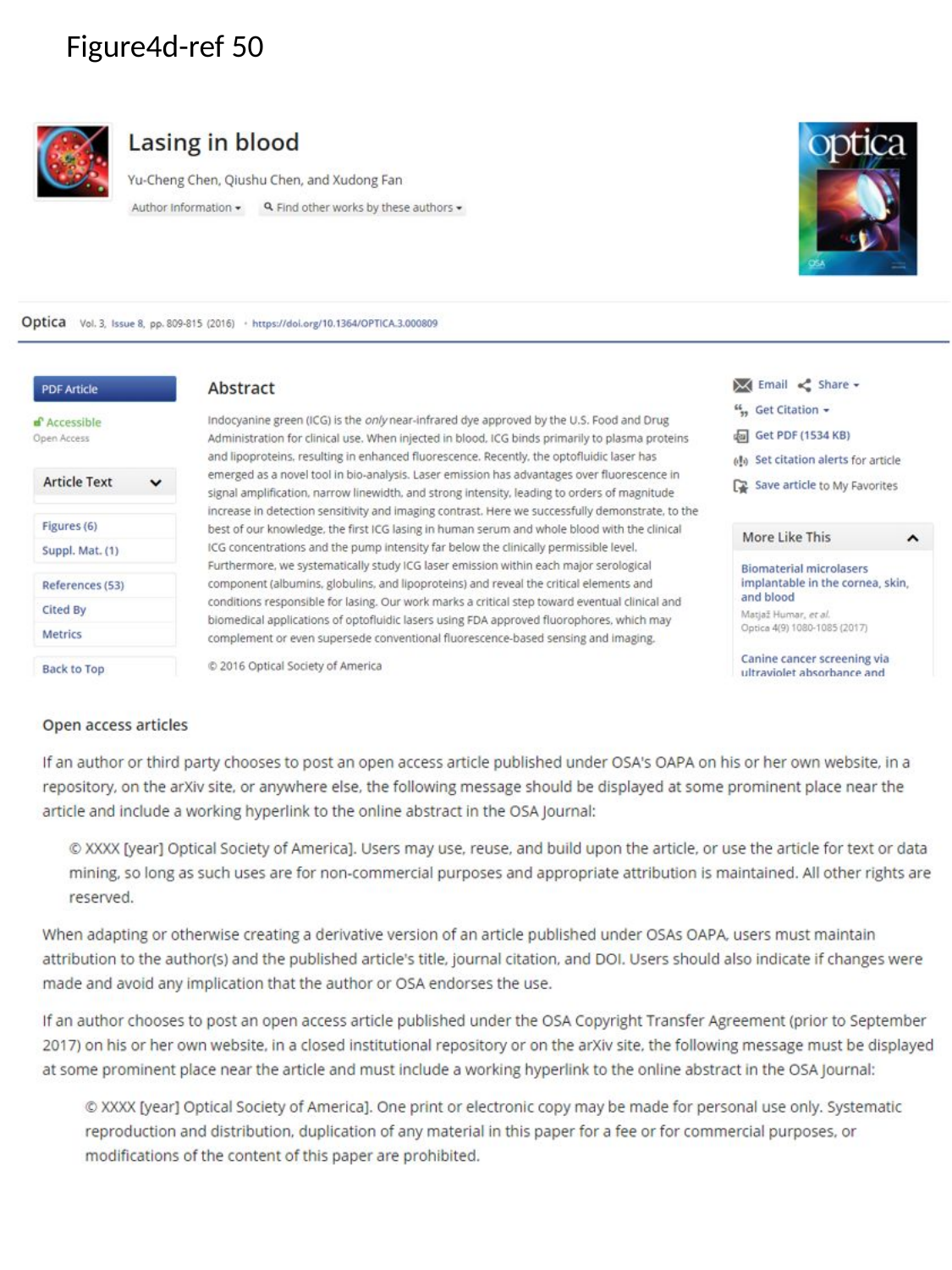

Figure4d-ref 50

## Slide 23
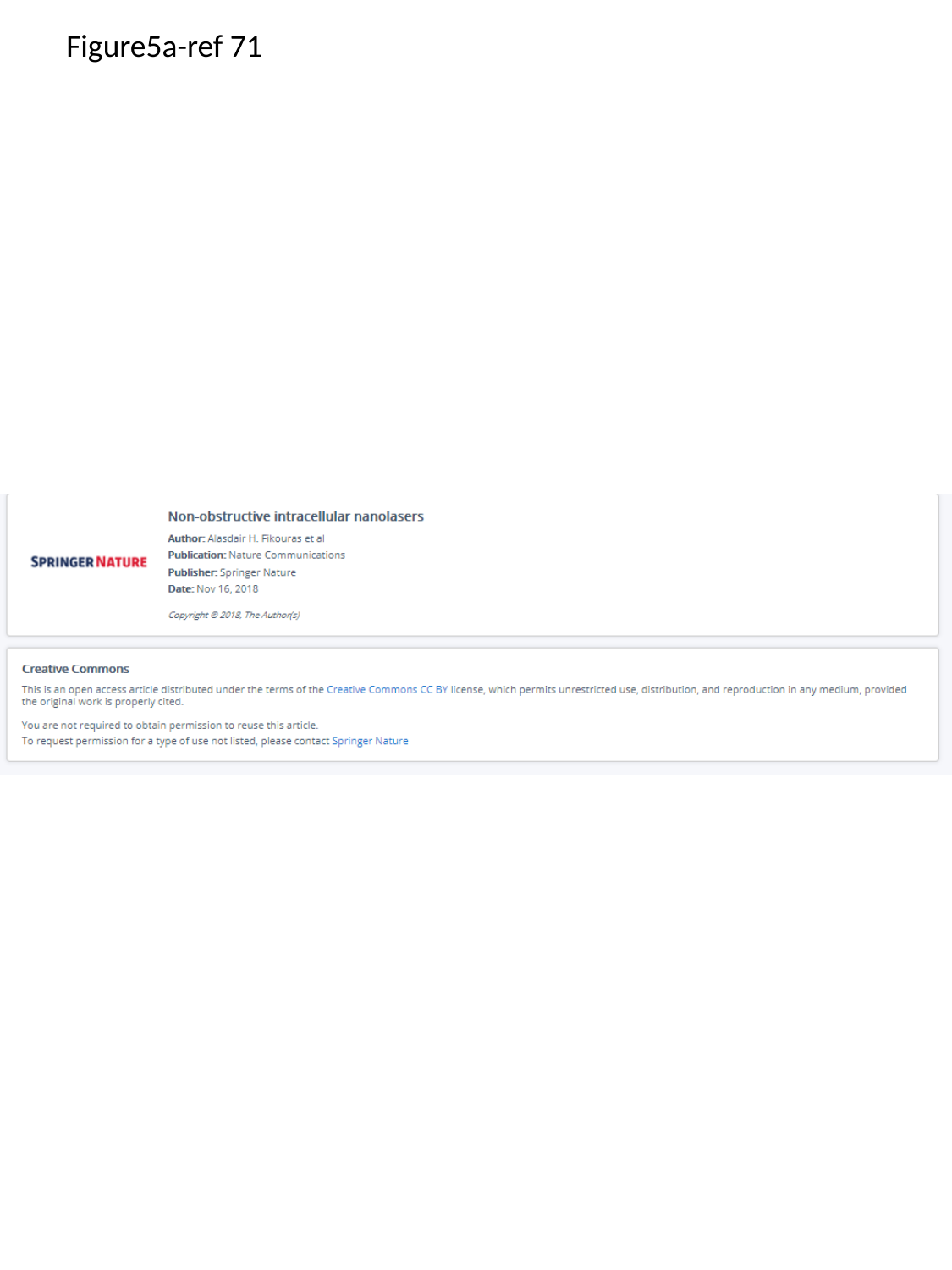

Figure5a-ref 71

## Slide 24
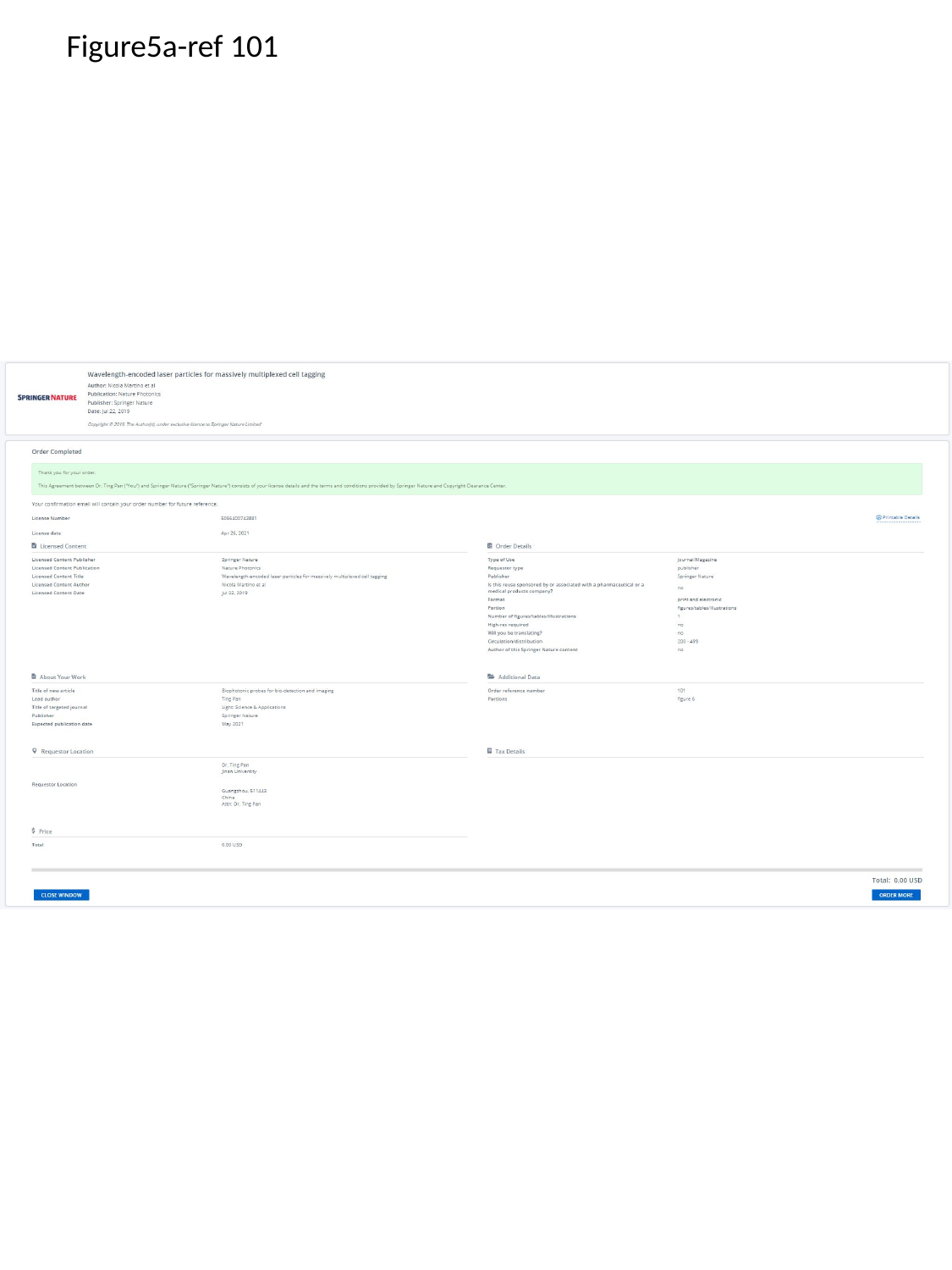

Figure5a-ref 101

## Slide 25
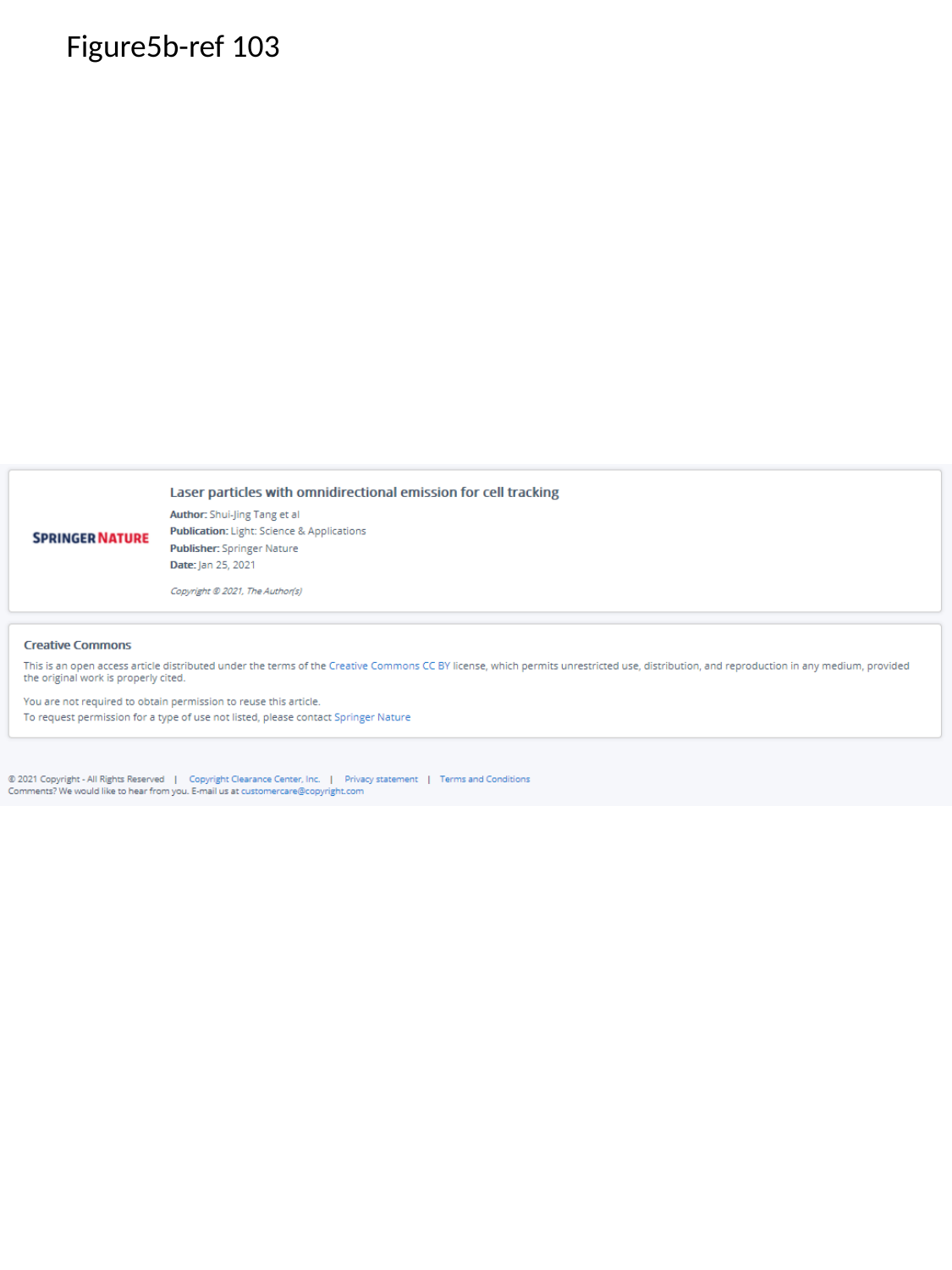

Figure5b-ref 103

## Slide 26
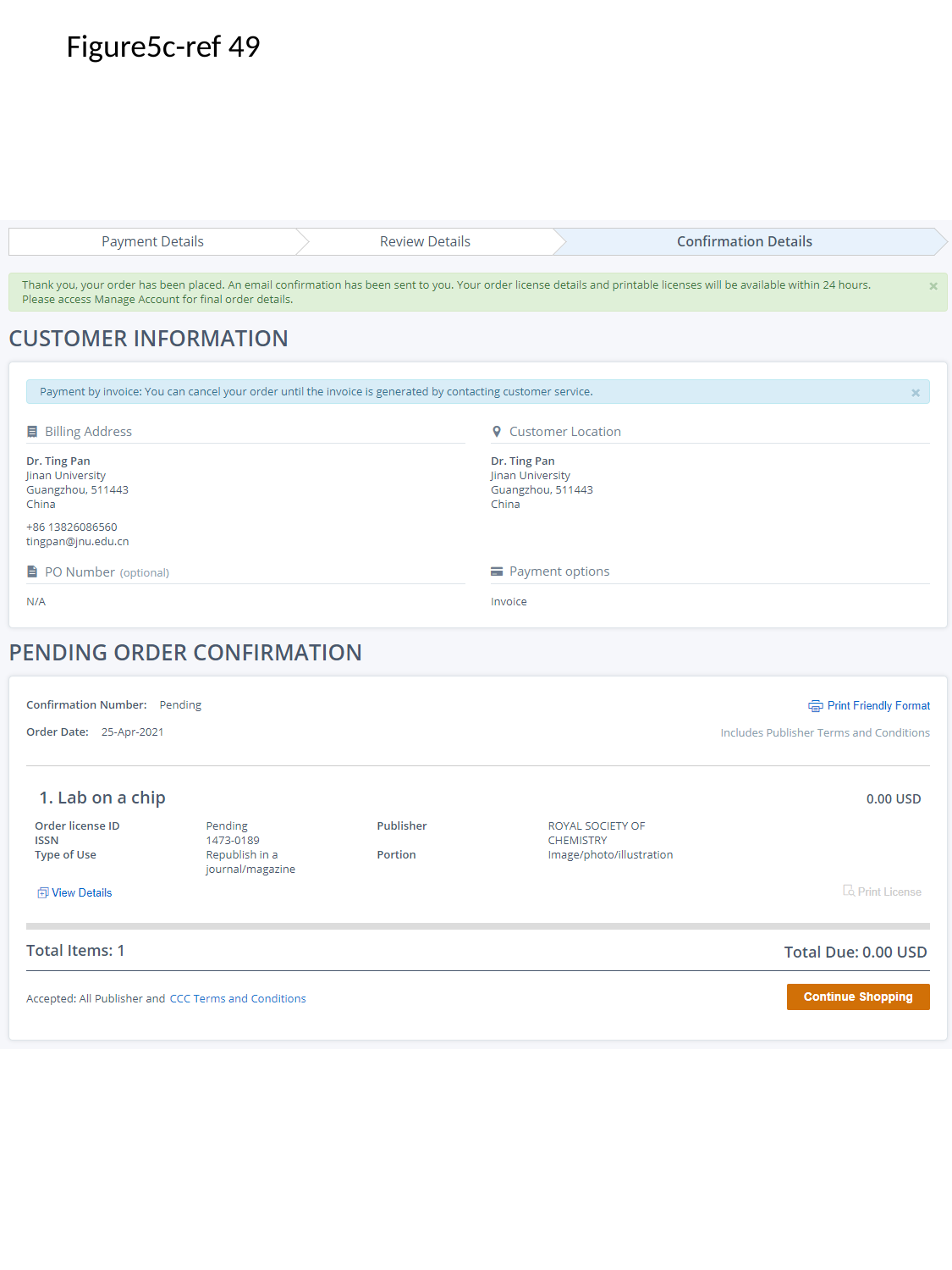

Figure5c-ref 49

## Slide 27
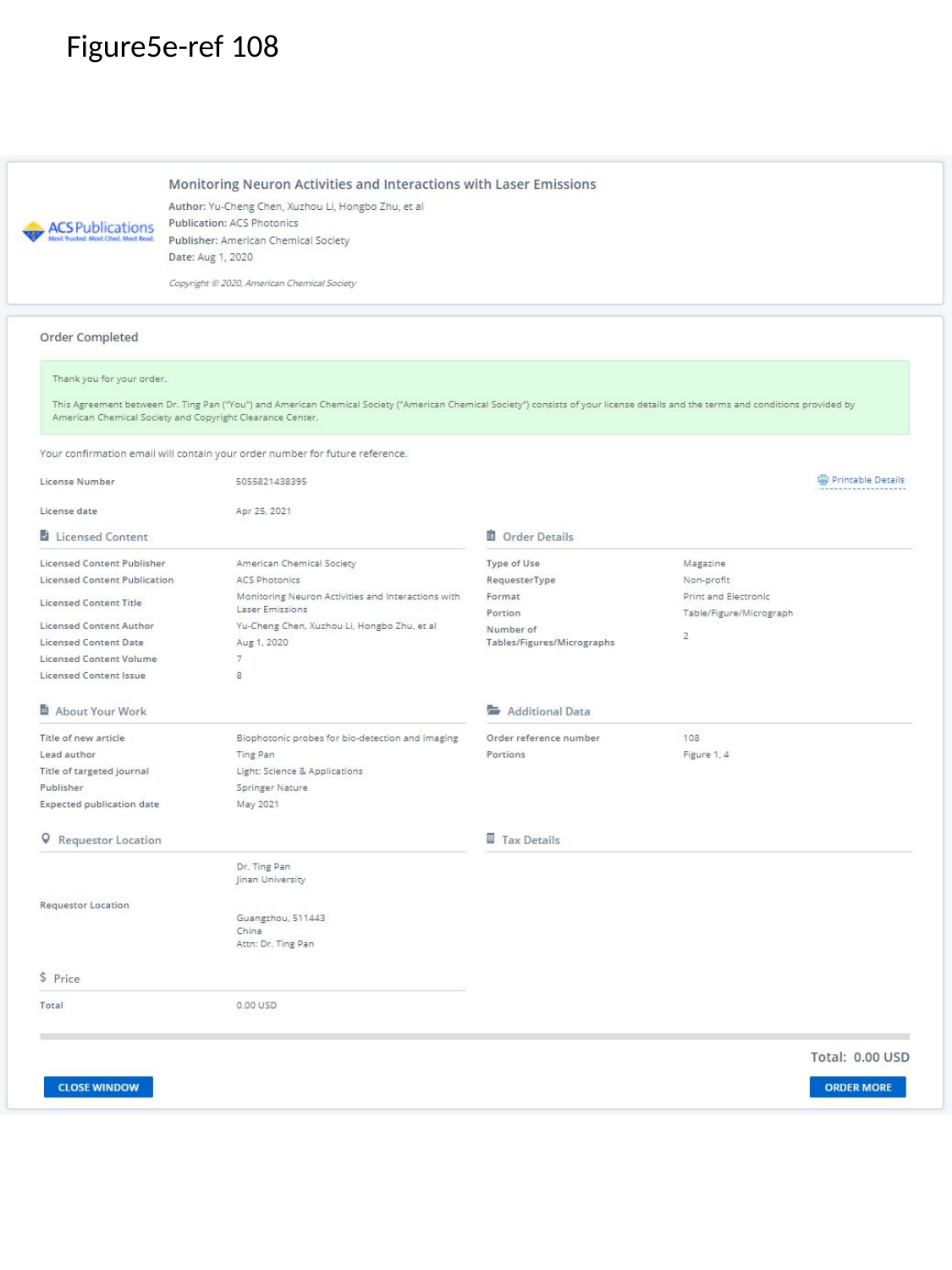

Figure5e-ref 108

## Slide 28
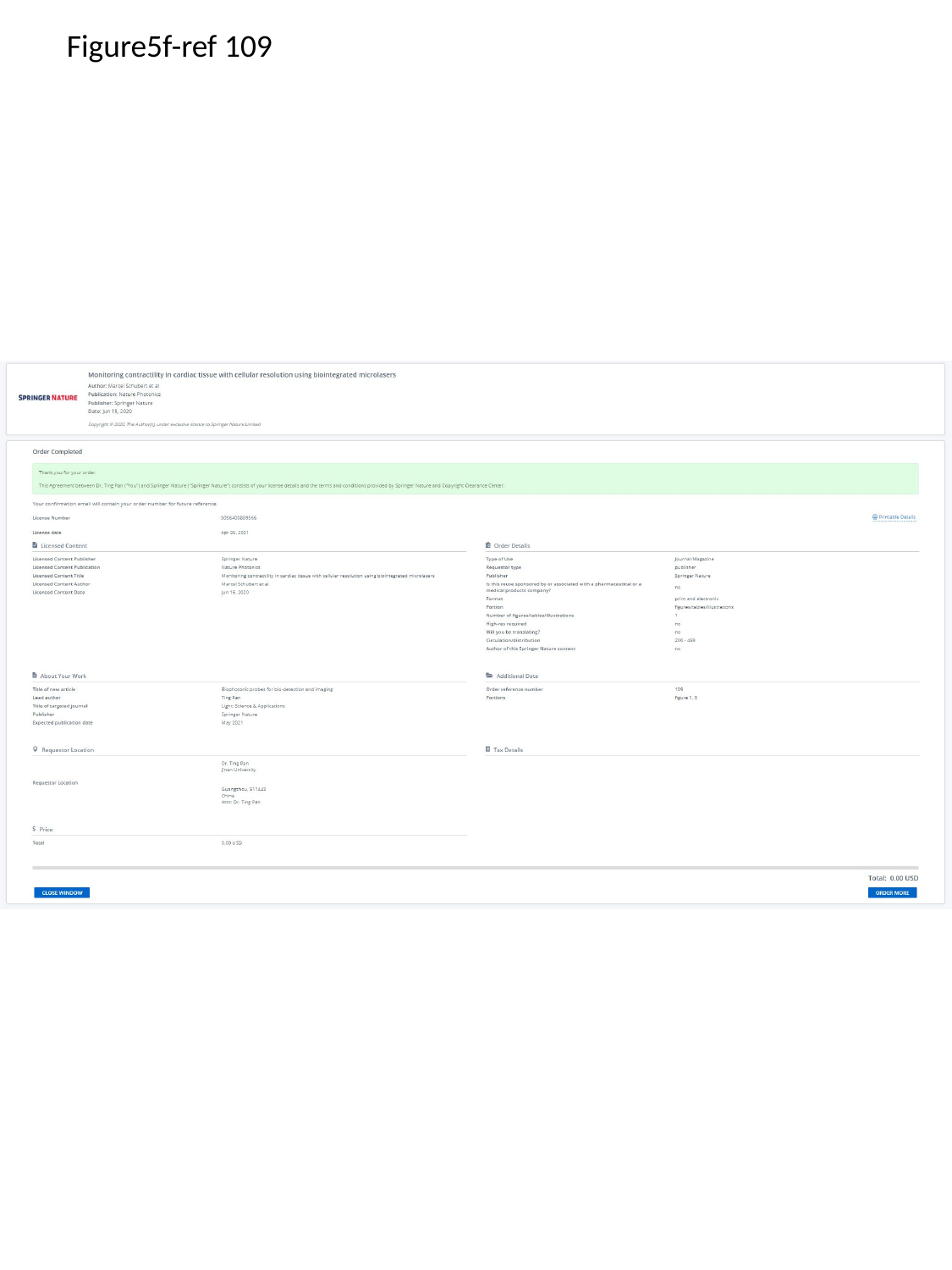

Figure5f-ref 109

## Slide 29
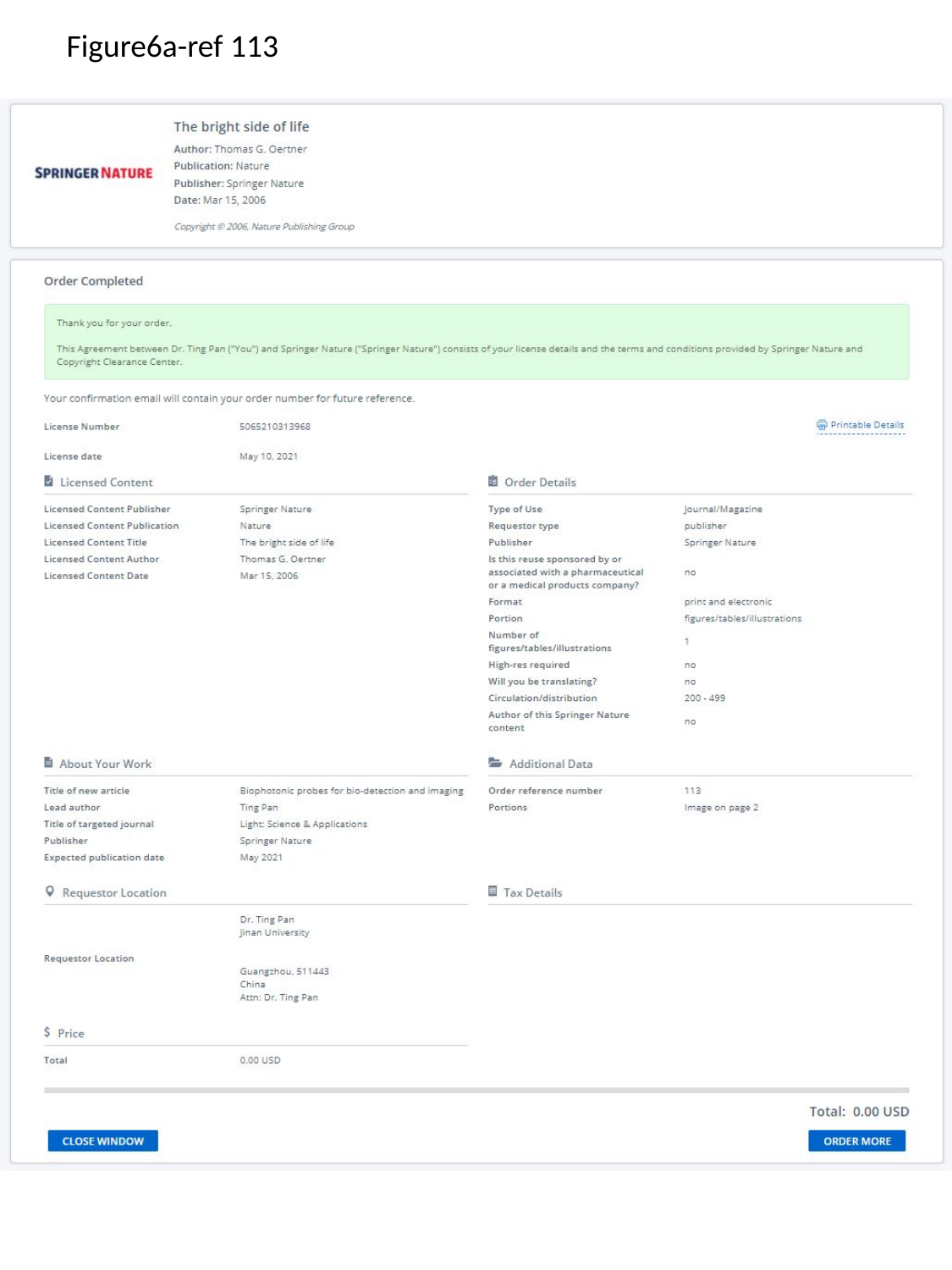

Figure6a-ref 113

## Slide 30
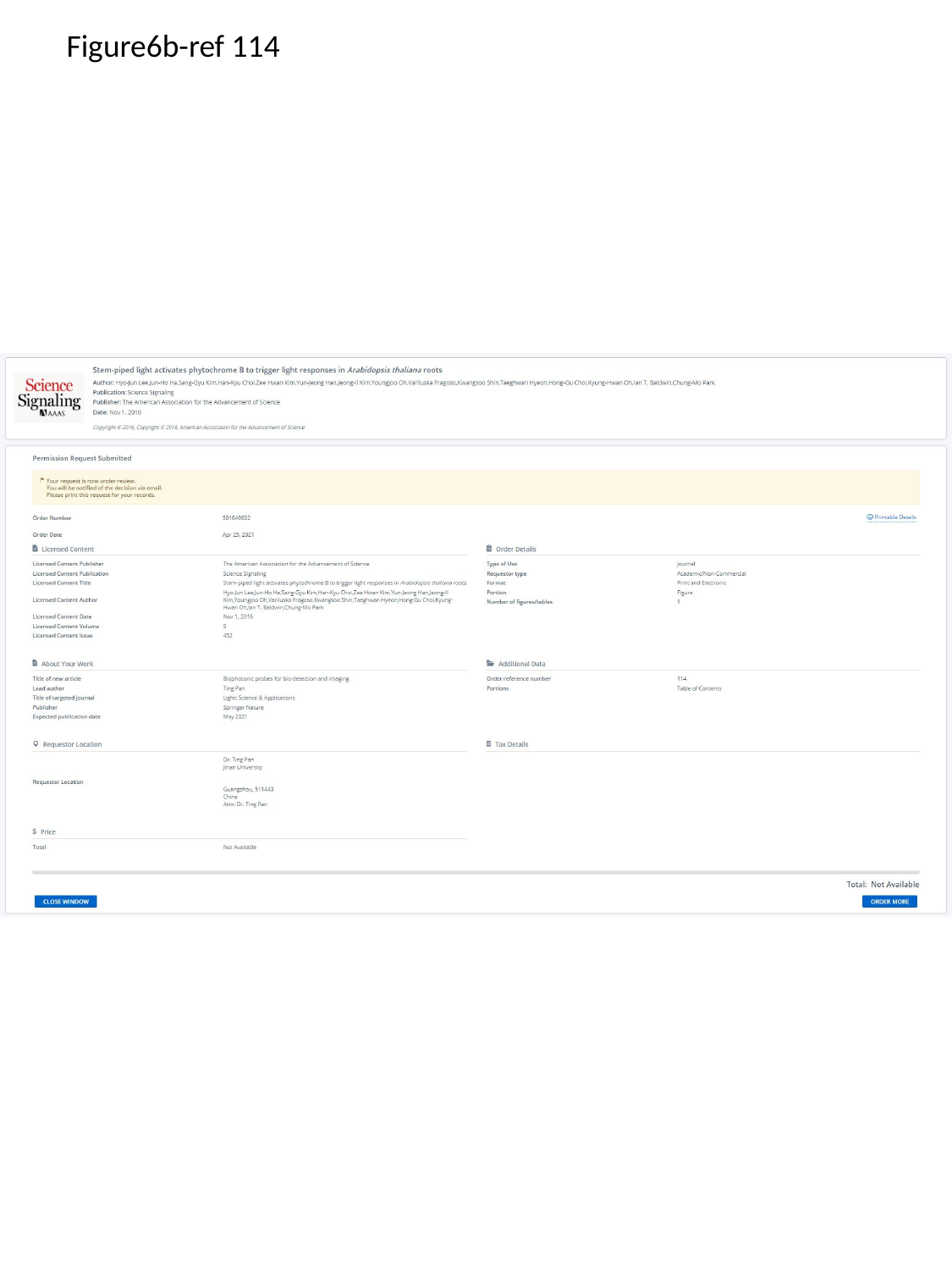

Figure6b-ref 114

## Slide 31
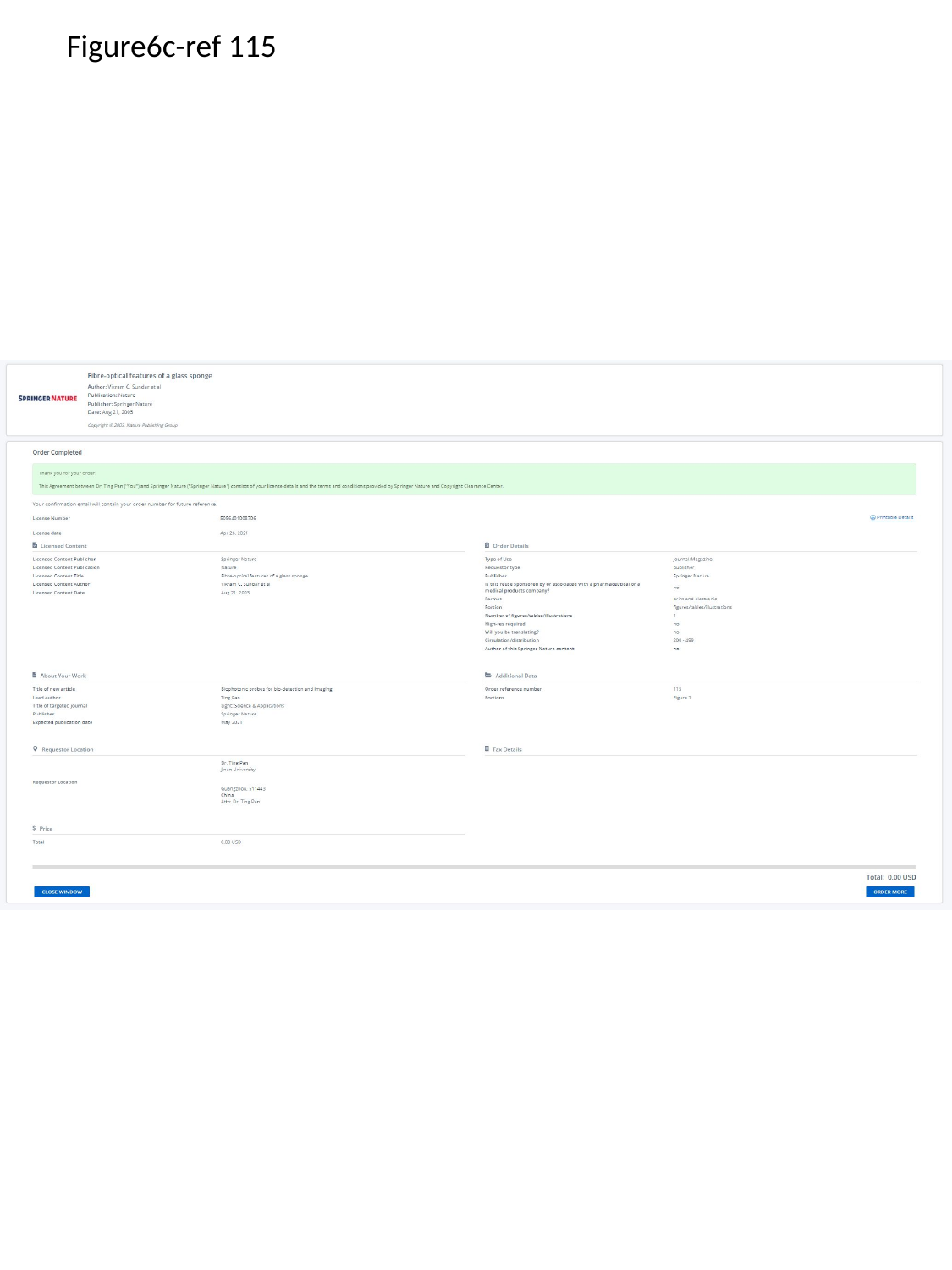

Figure6c-ref 115

## Slide 32
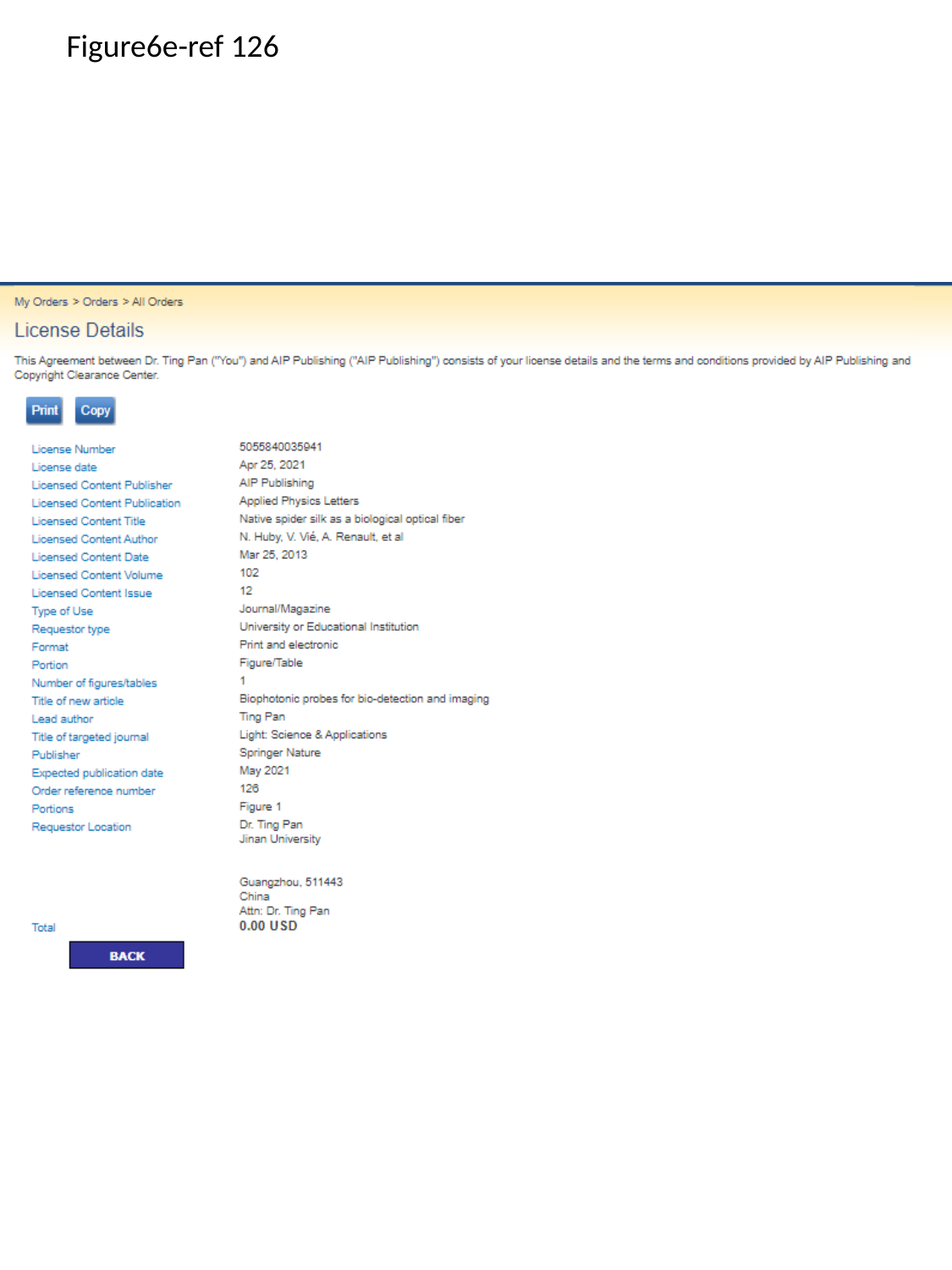

Figure6e-ref 126

## Slide 33
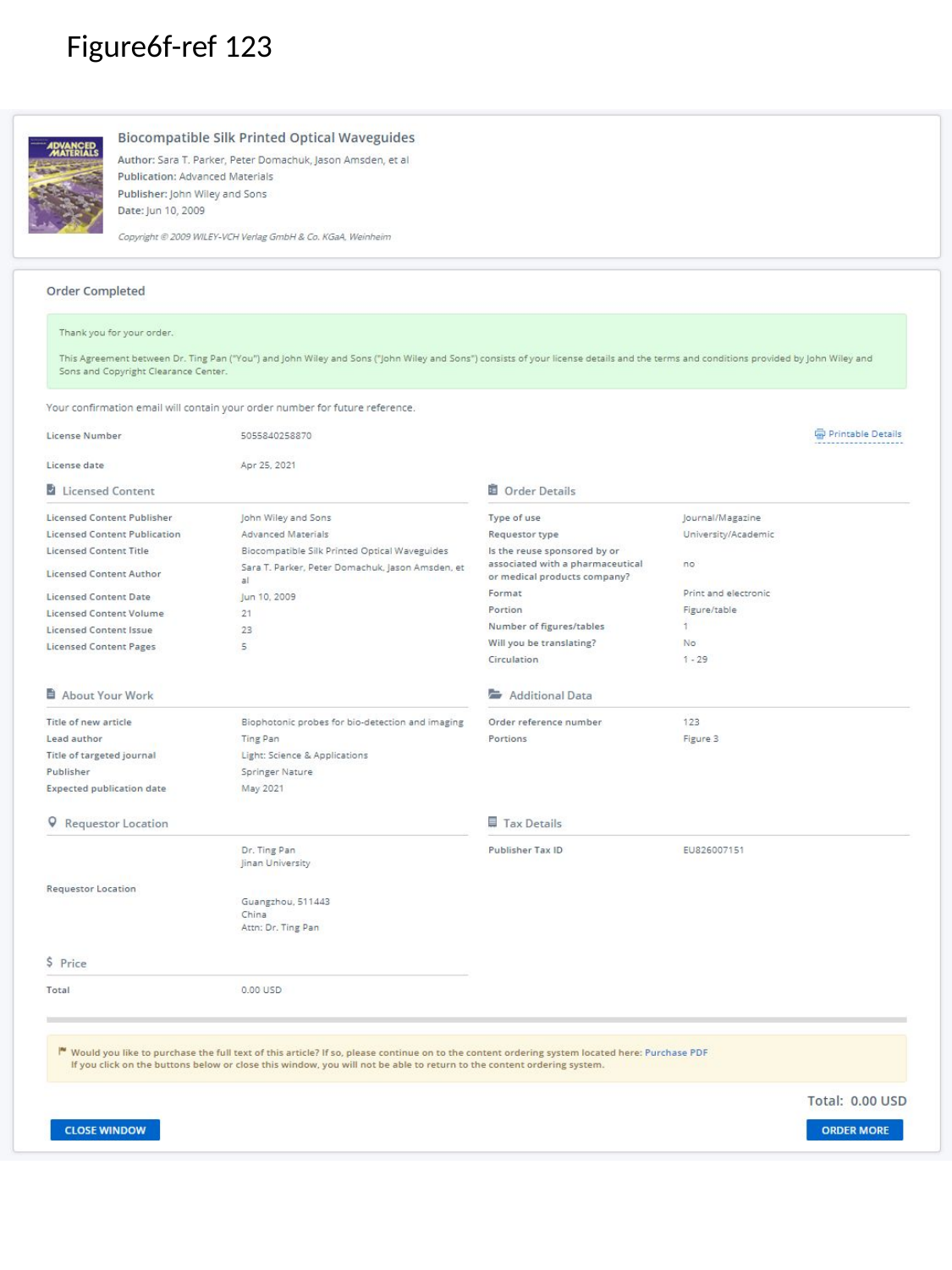

Figure6f-ref 123

## Slide 34
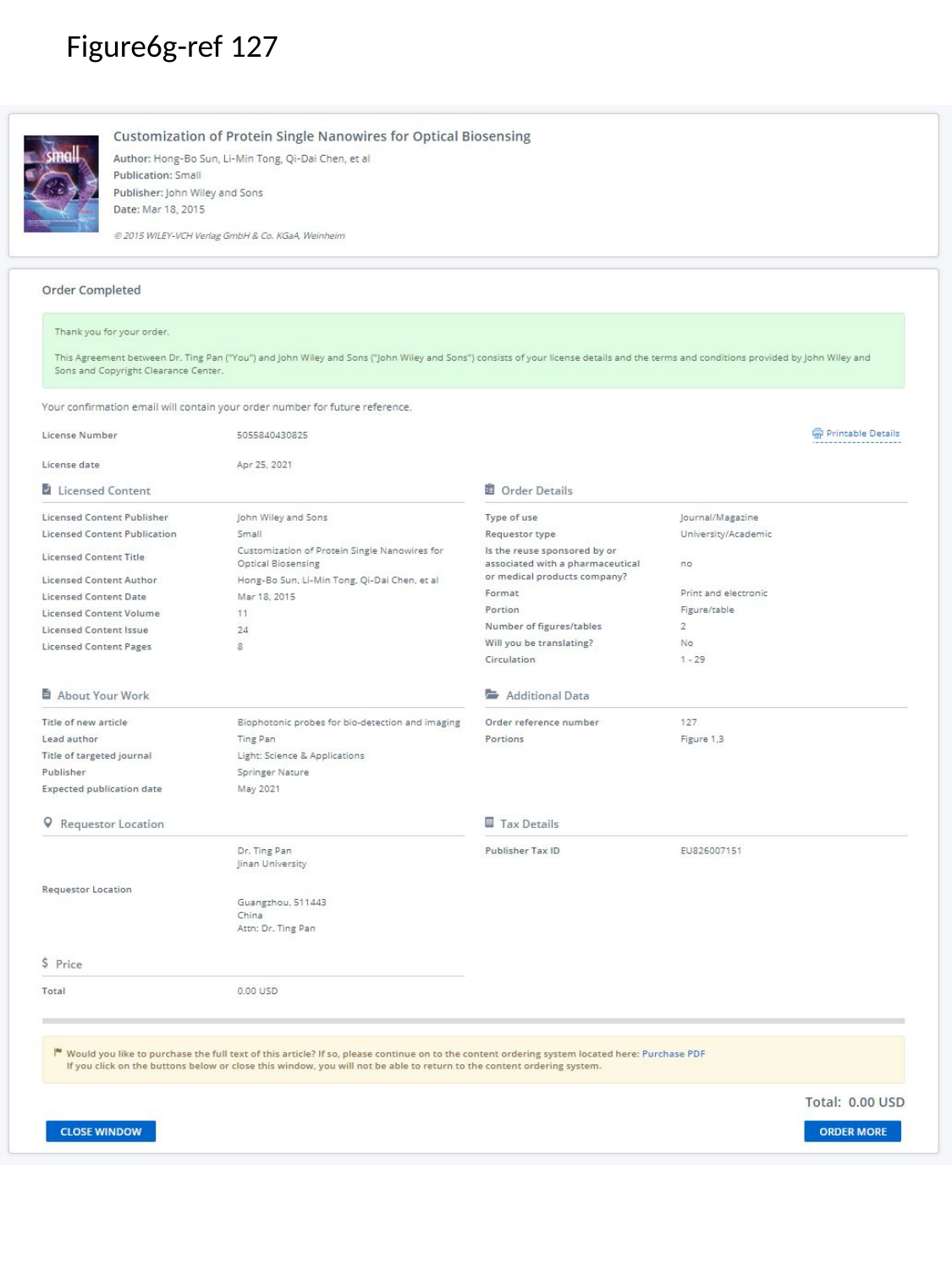

Figure6g-ref 127

## Slide 35
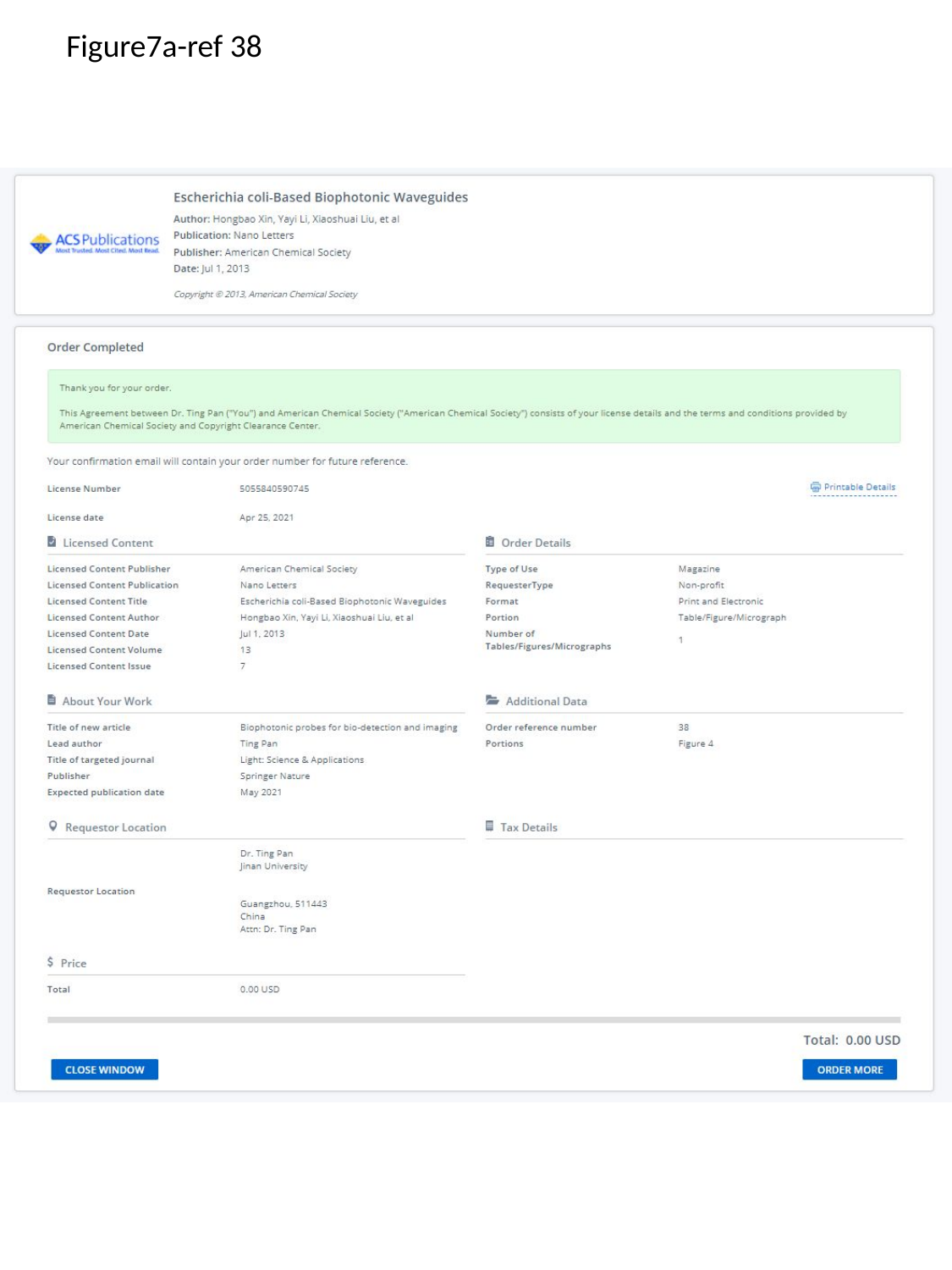

Figure7a-ref 38

## Slide 36
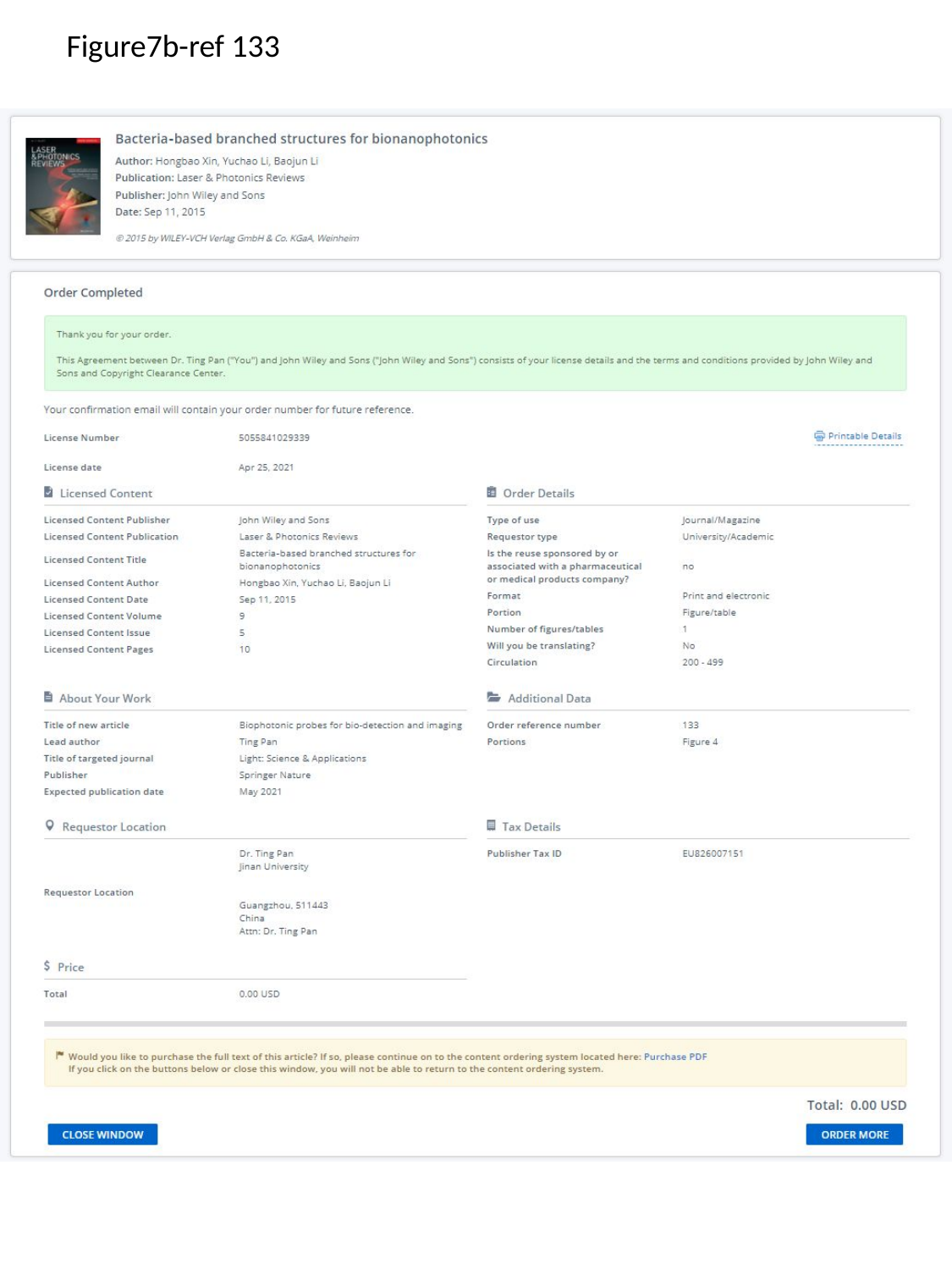

Figure7b-ref 133

## Slide 37
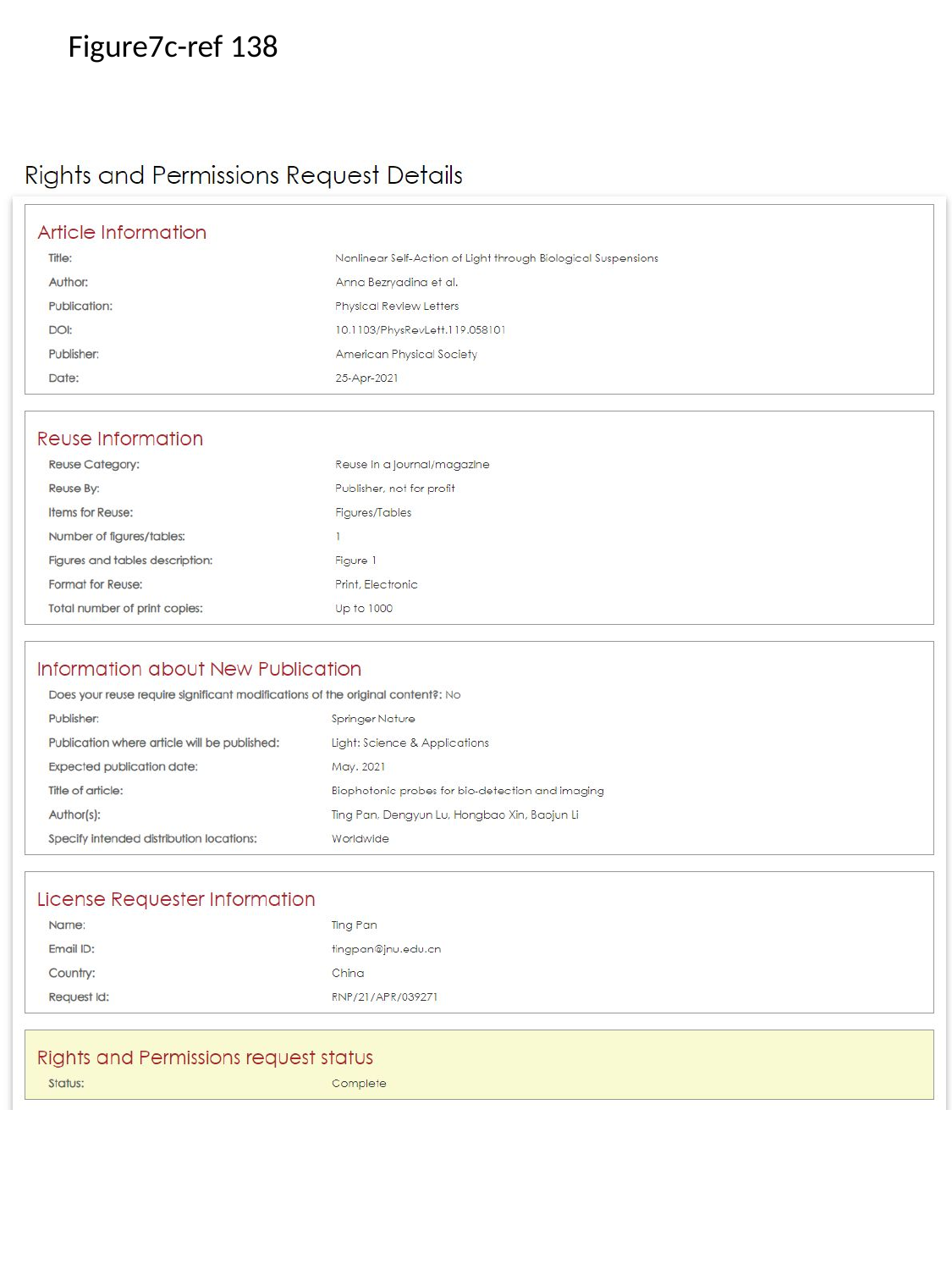

Figure7c-ref 138

## Slide 38
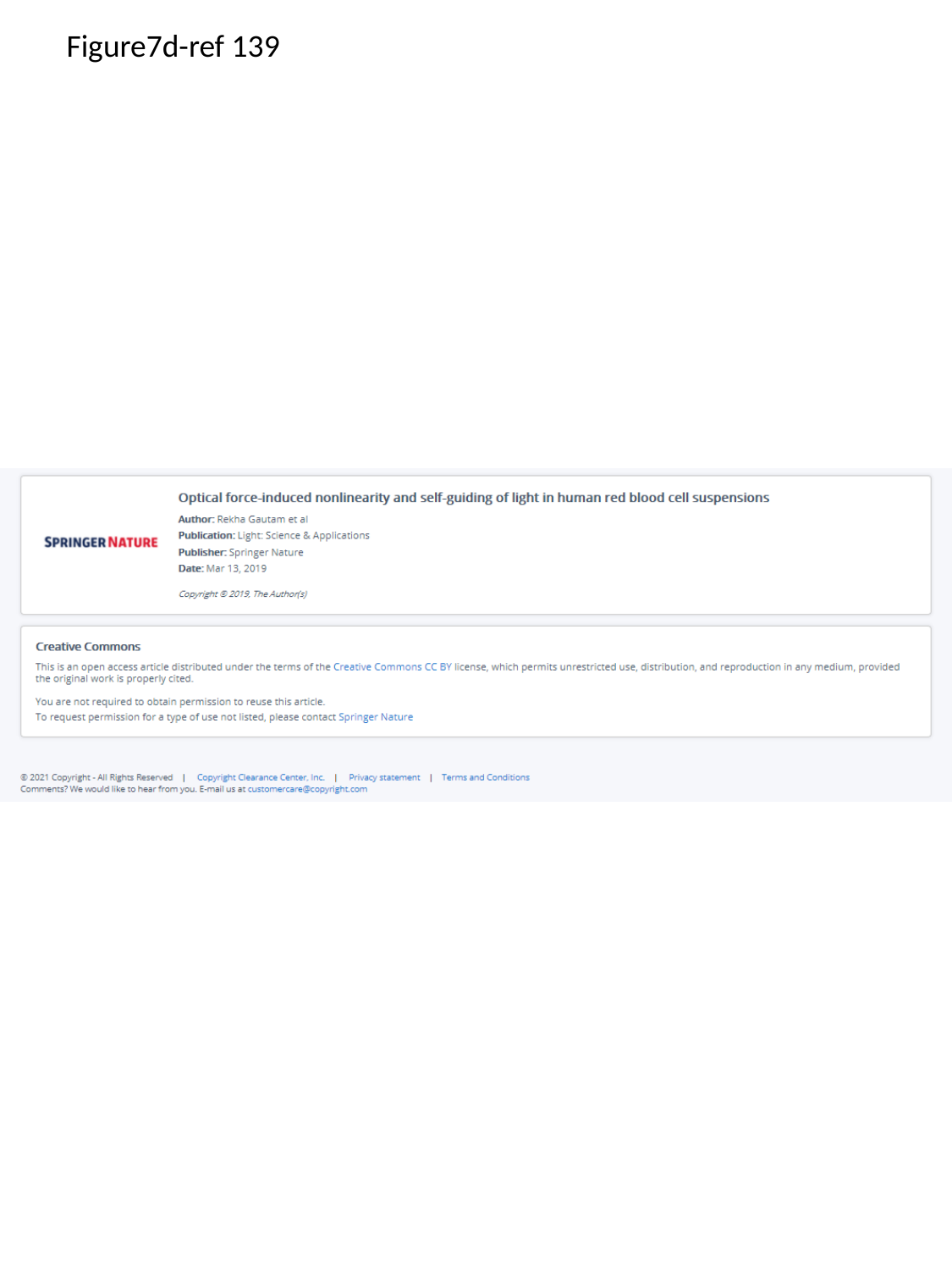

Figure7d-ref 139

## Slide 39
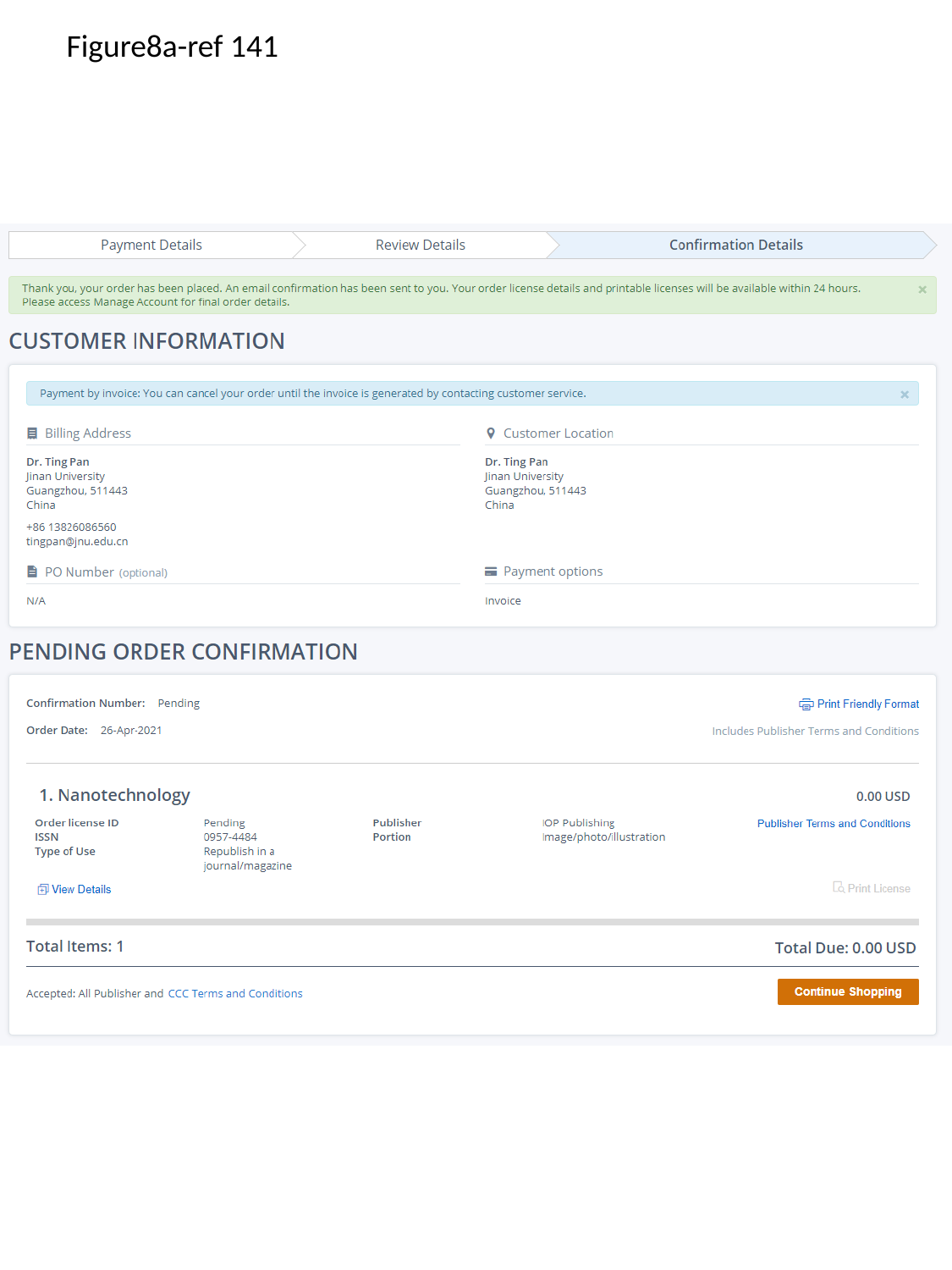

Figure8a-ref 141

## Slide 40
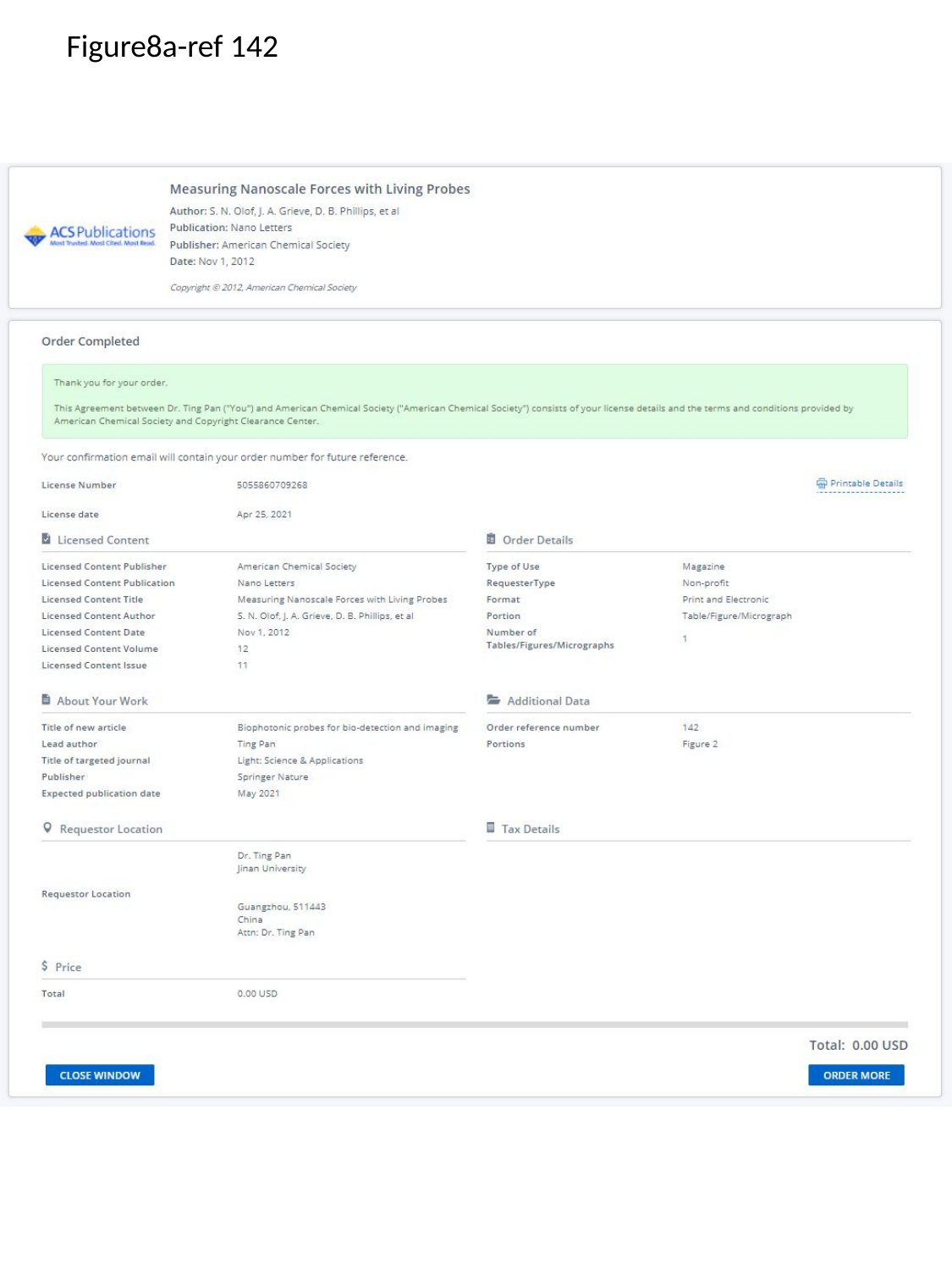

Figure8a-ref 142

## Slide 41
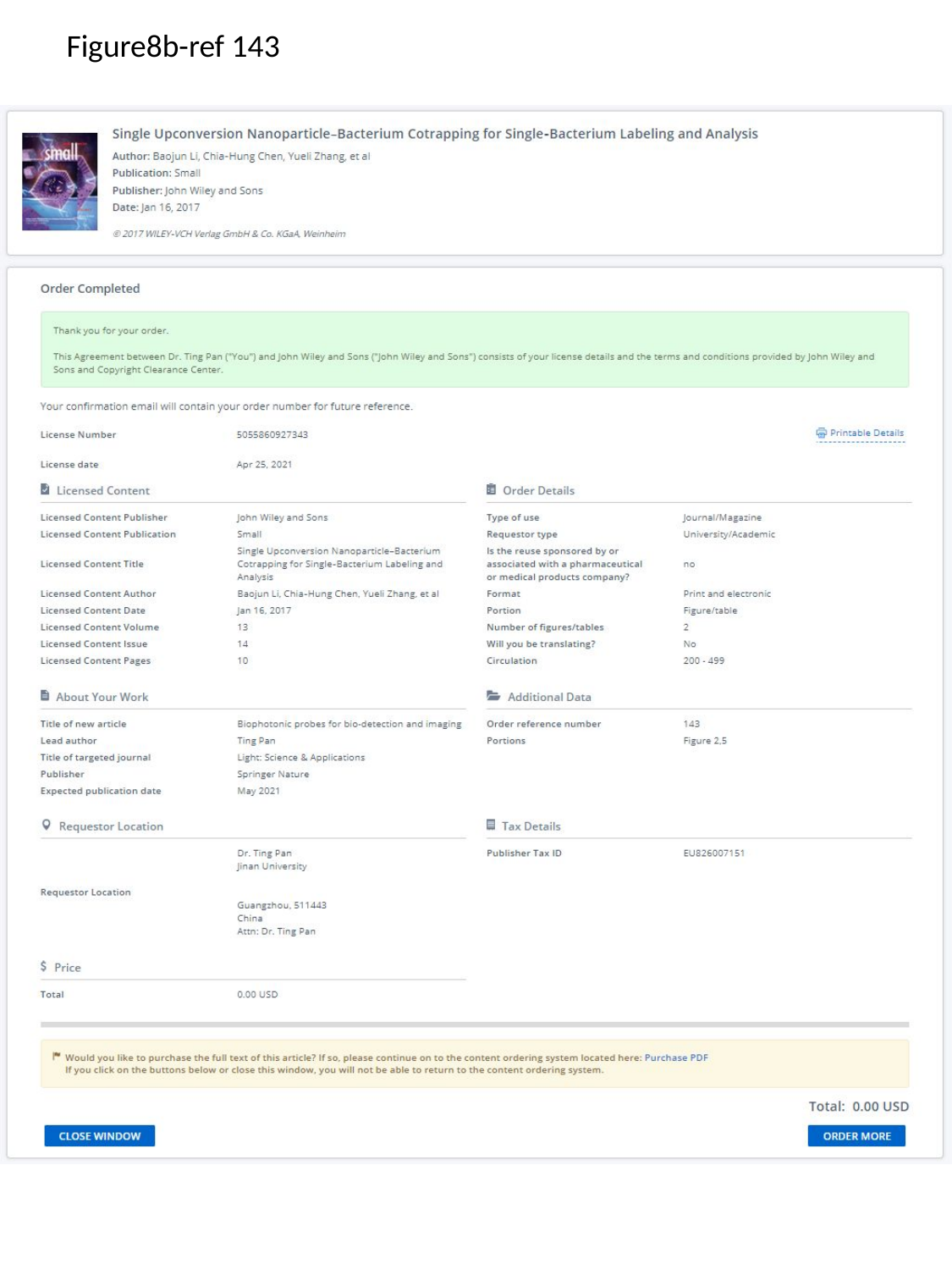

Figure8b-ref 143

## Slide 42
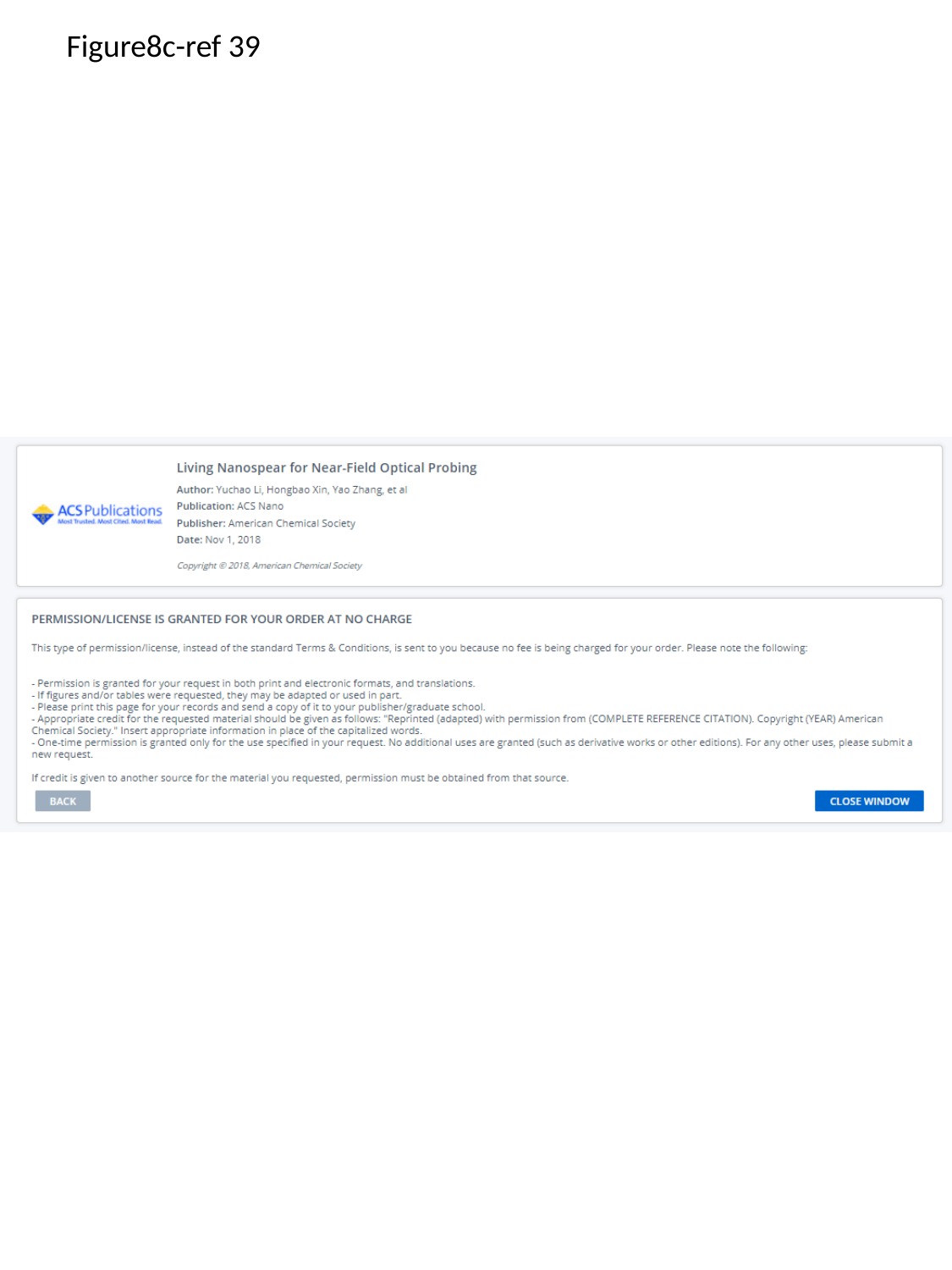

Figure8c-ref 39

## Slide 43
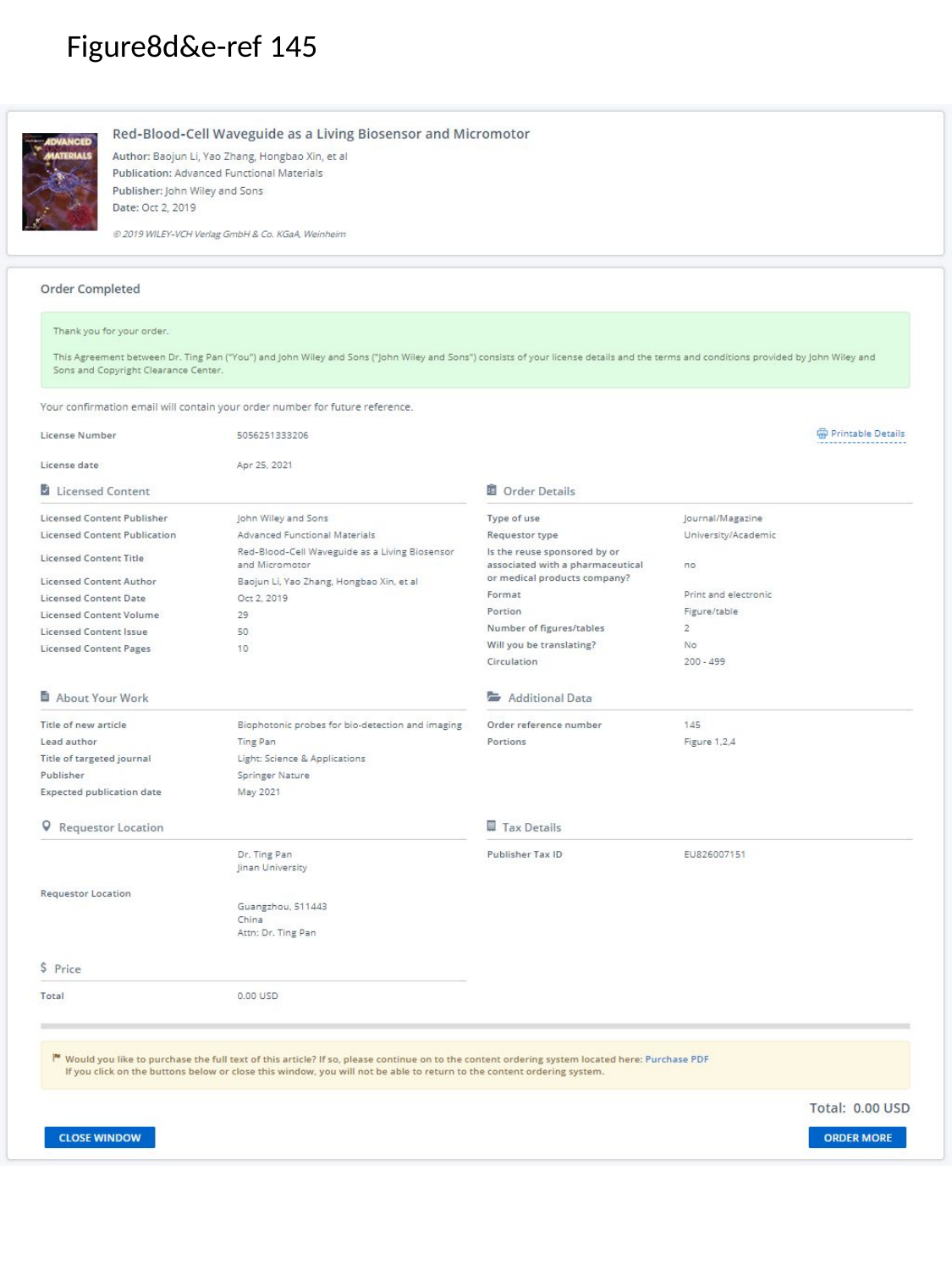

Figure8d&e-ref 145

## Slide 44
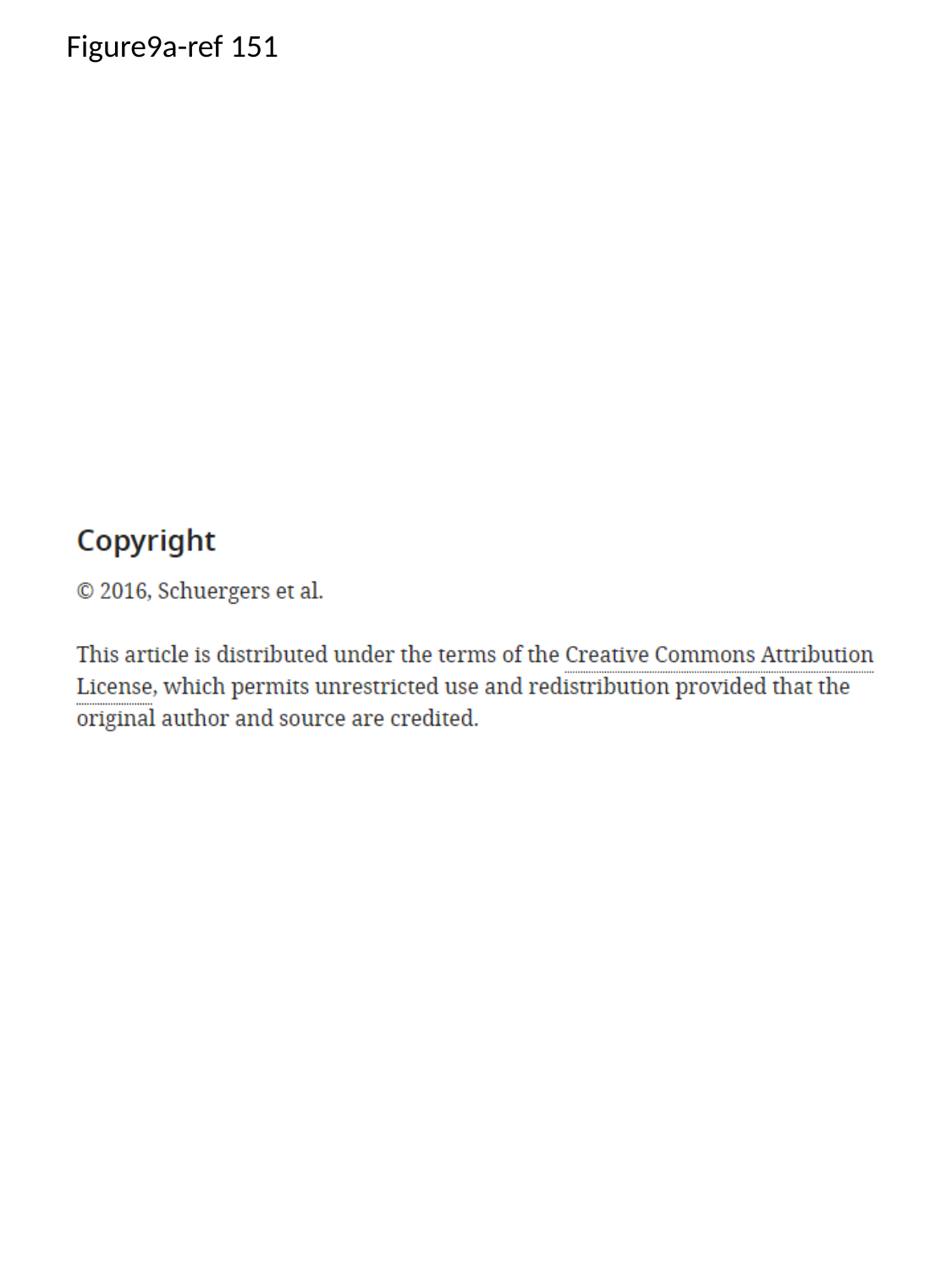

Figure9a-ref 151

## Slide 45
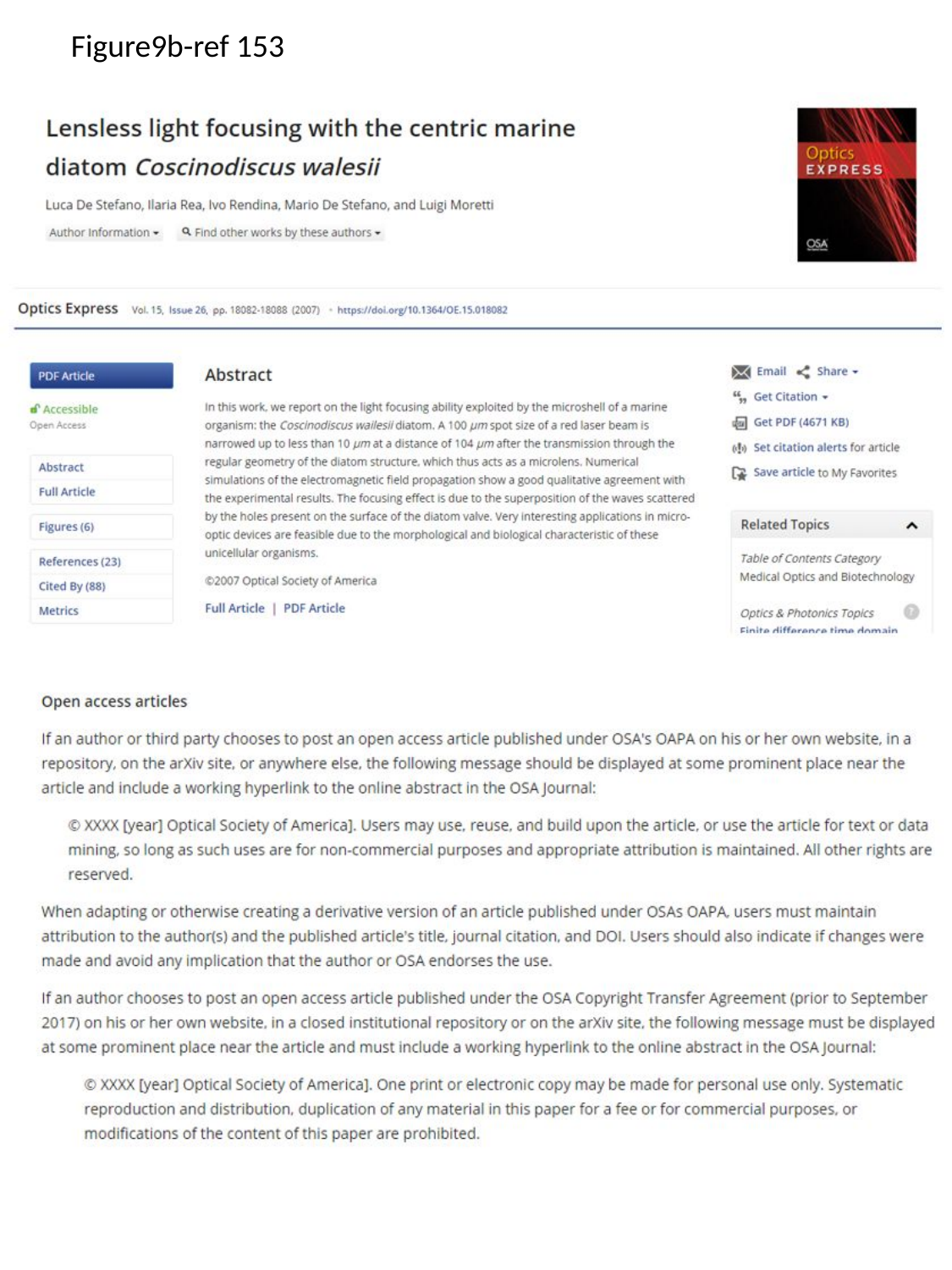

Figure9b-ref 153

## Slide 46
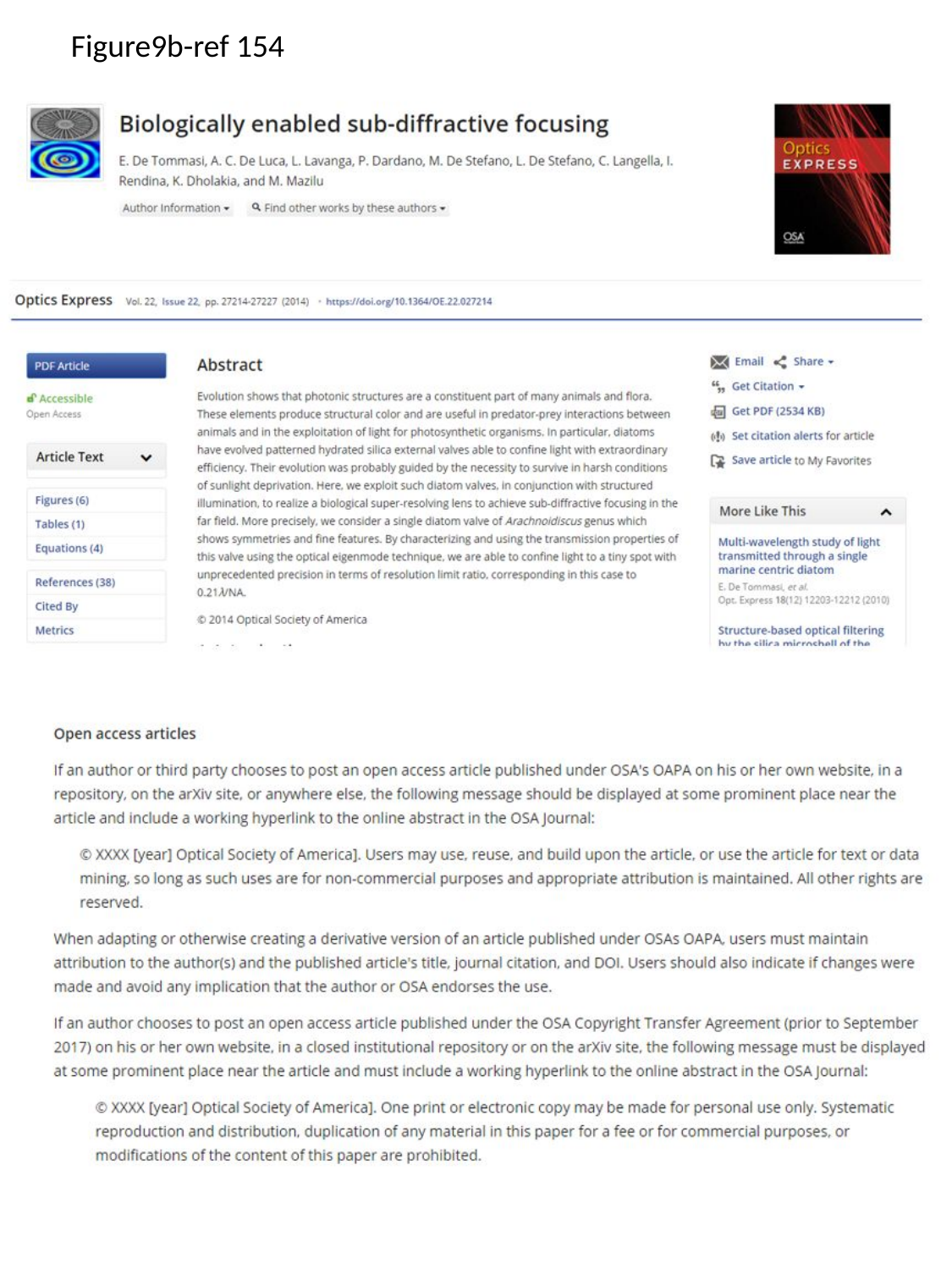

Figure9b-ref 154

## Slide 47
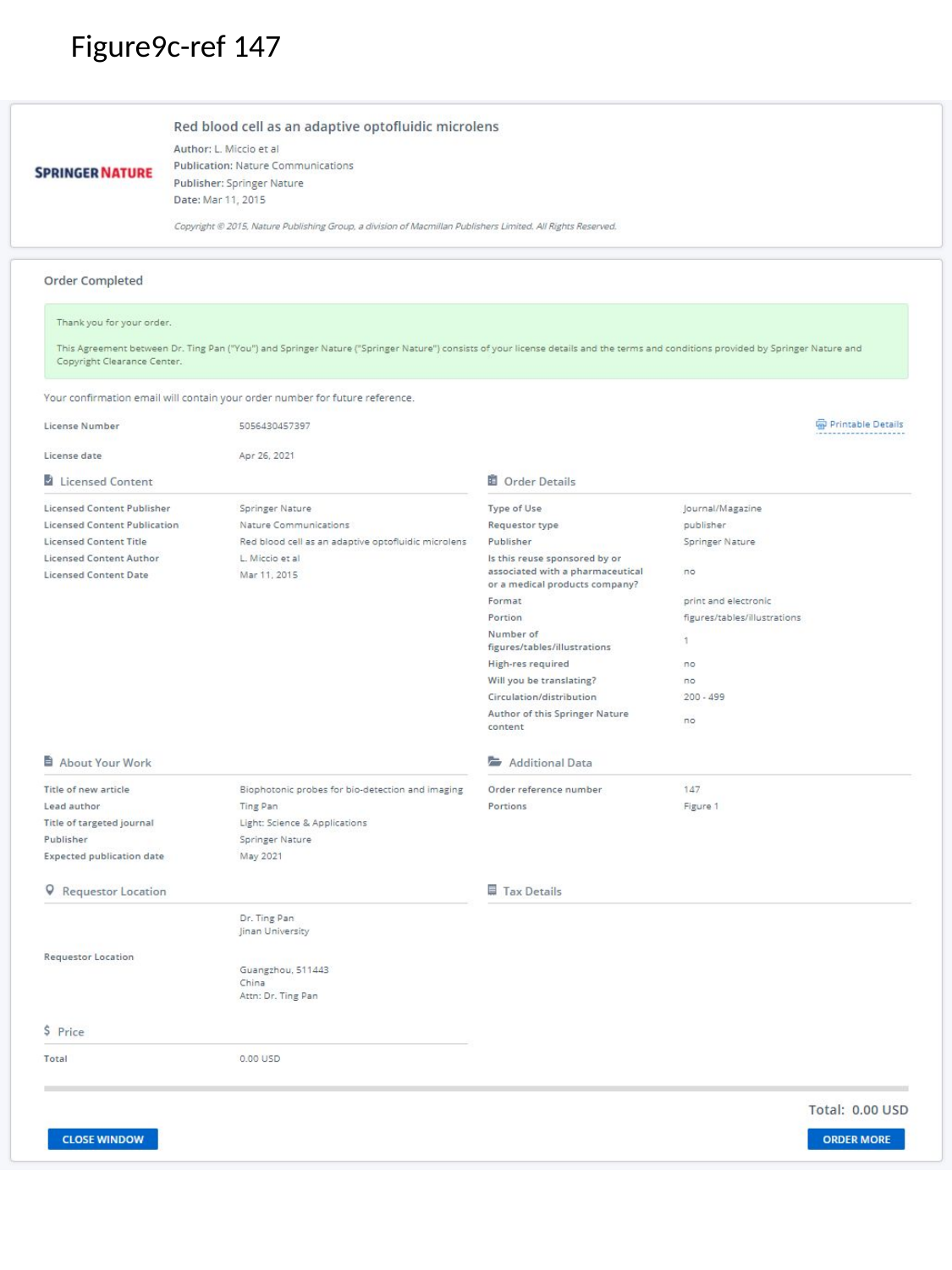

Figure9c-ref 147

## Slide 48
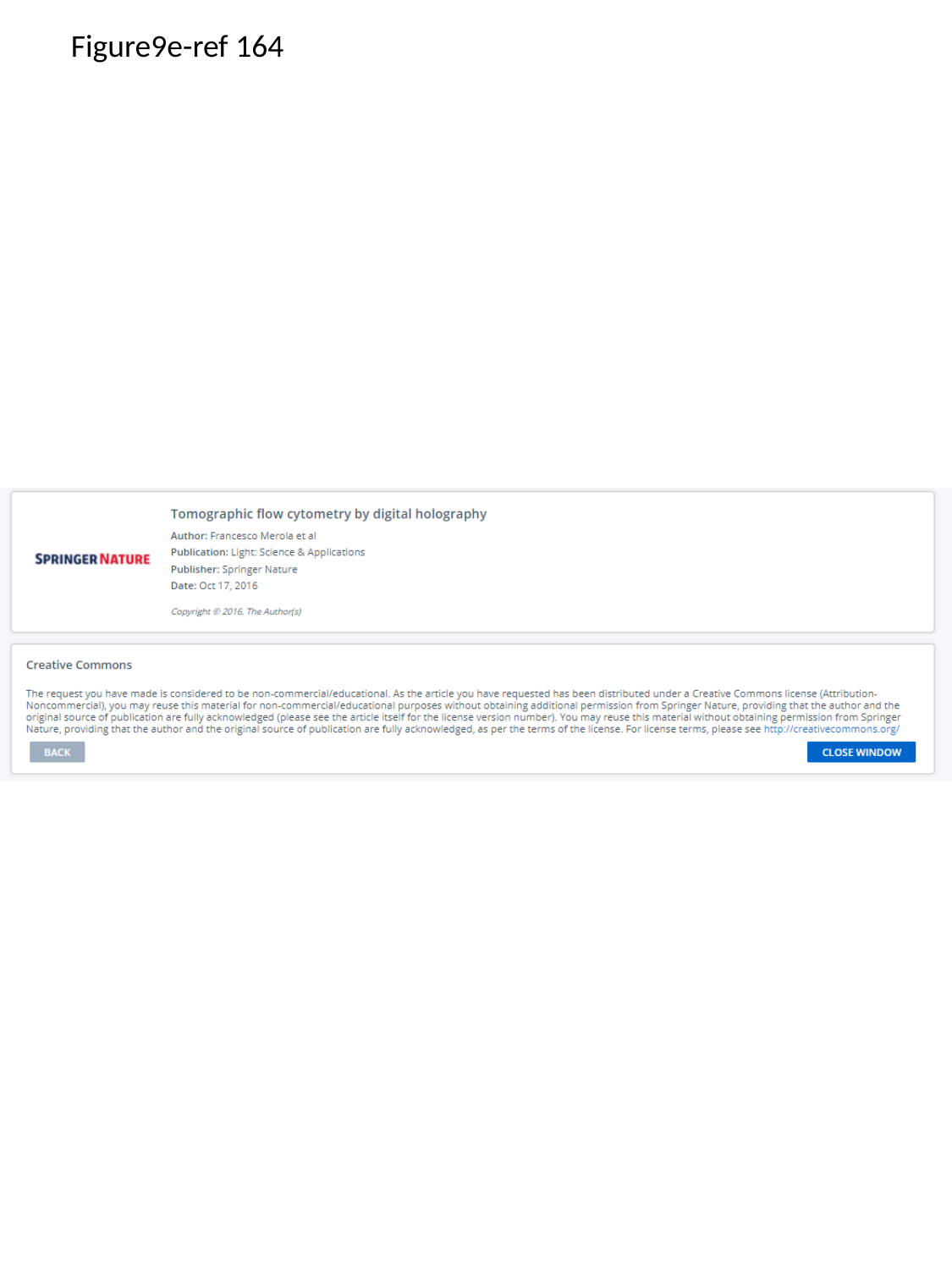

Figure9e-ref 164

## Slide 49
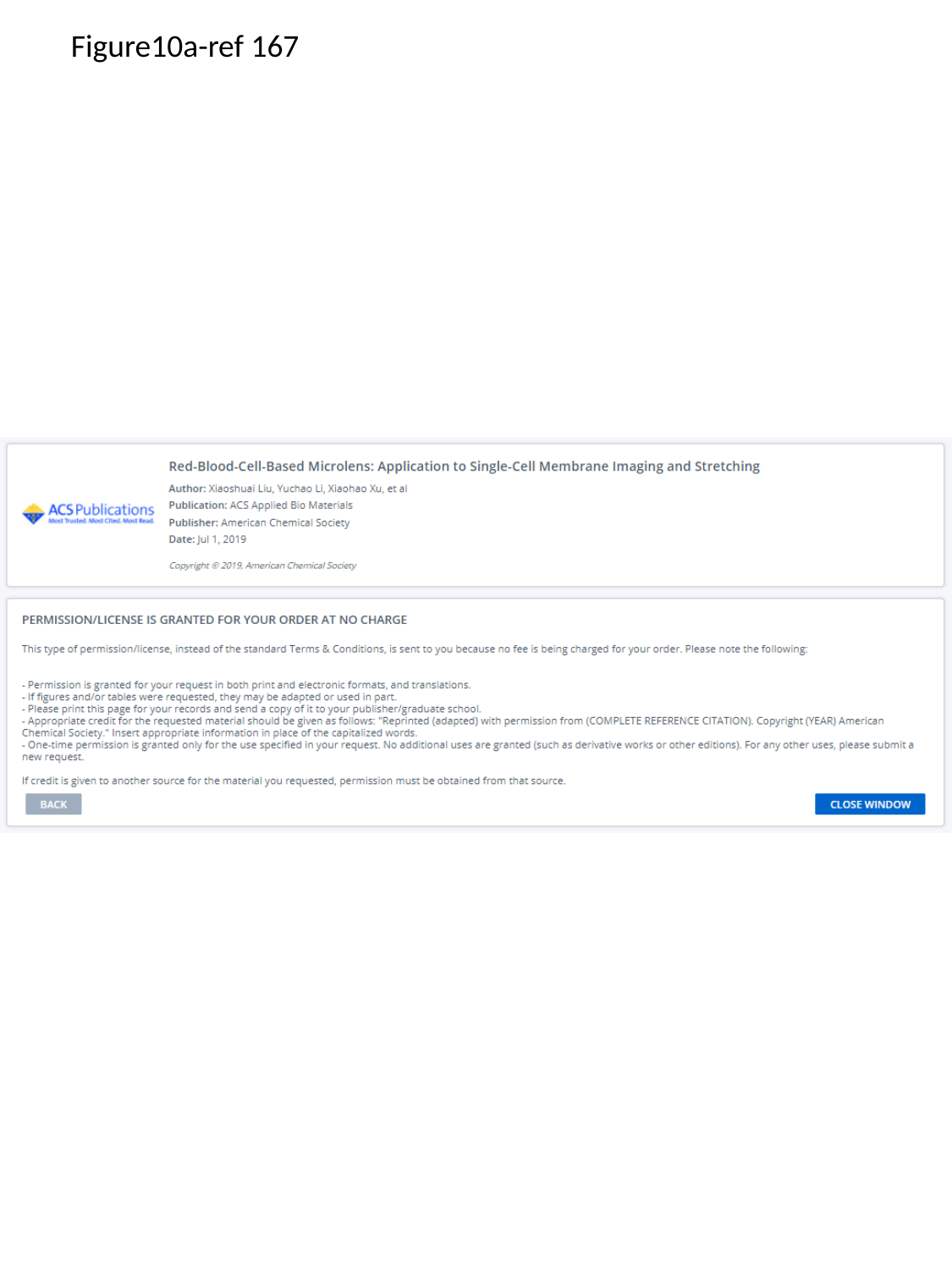

Figure10a-ref 167

## Slide 50
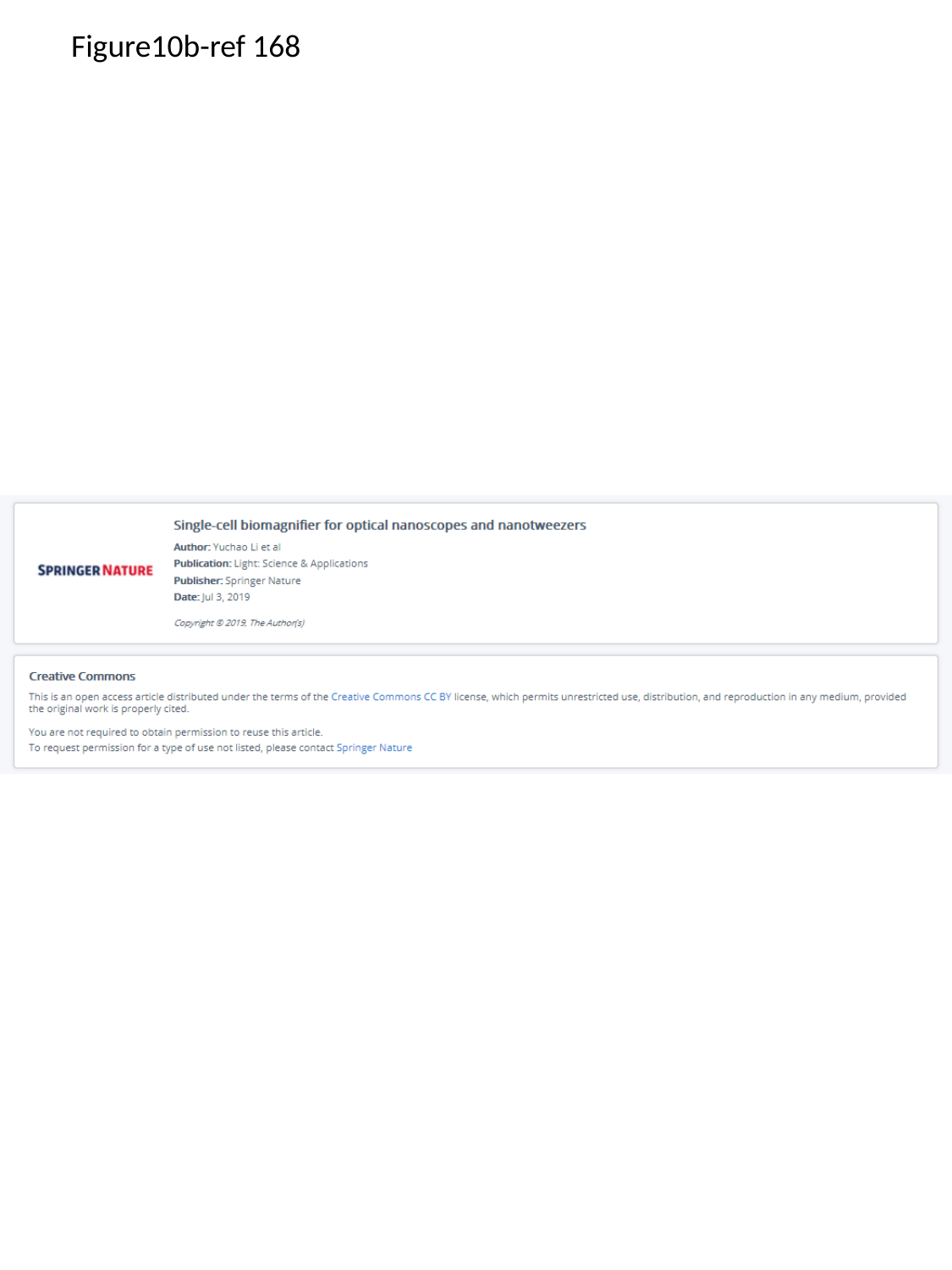

Figure10b-ref 168

## Slide 51
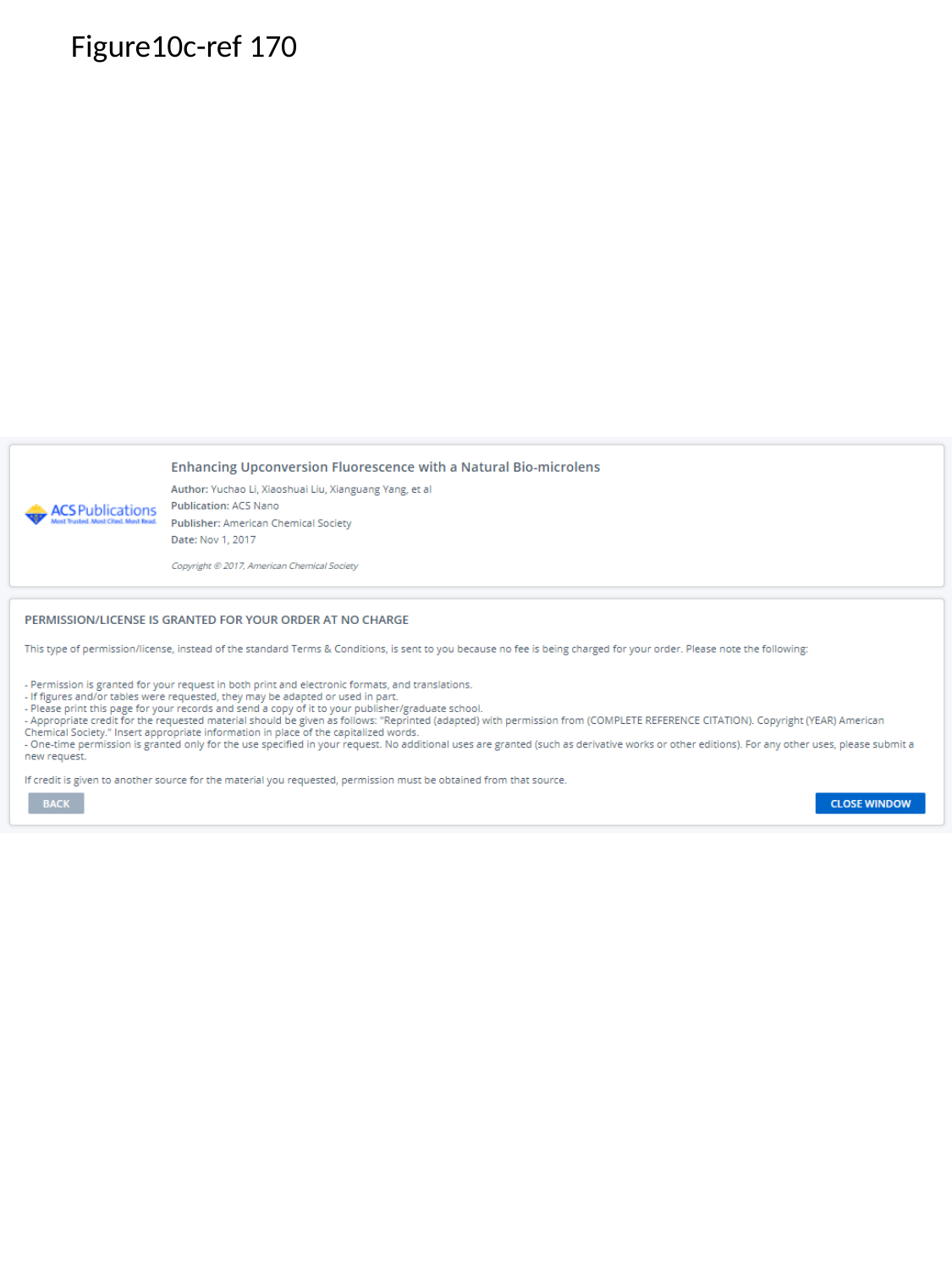

Figure10c-ref 170

## Slide 52
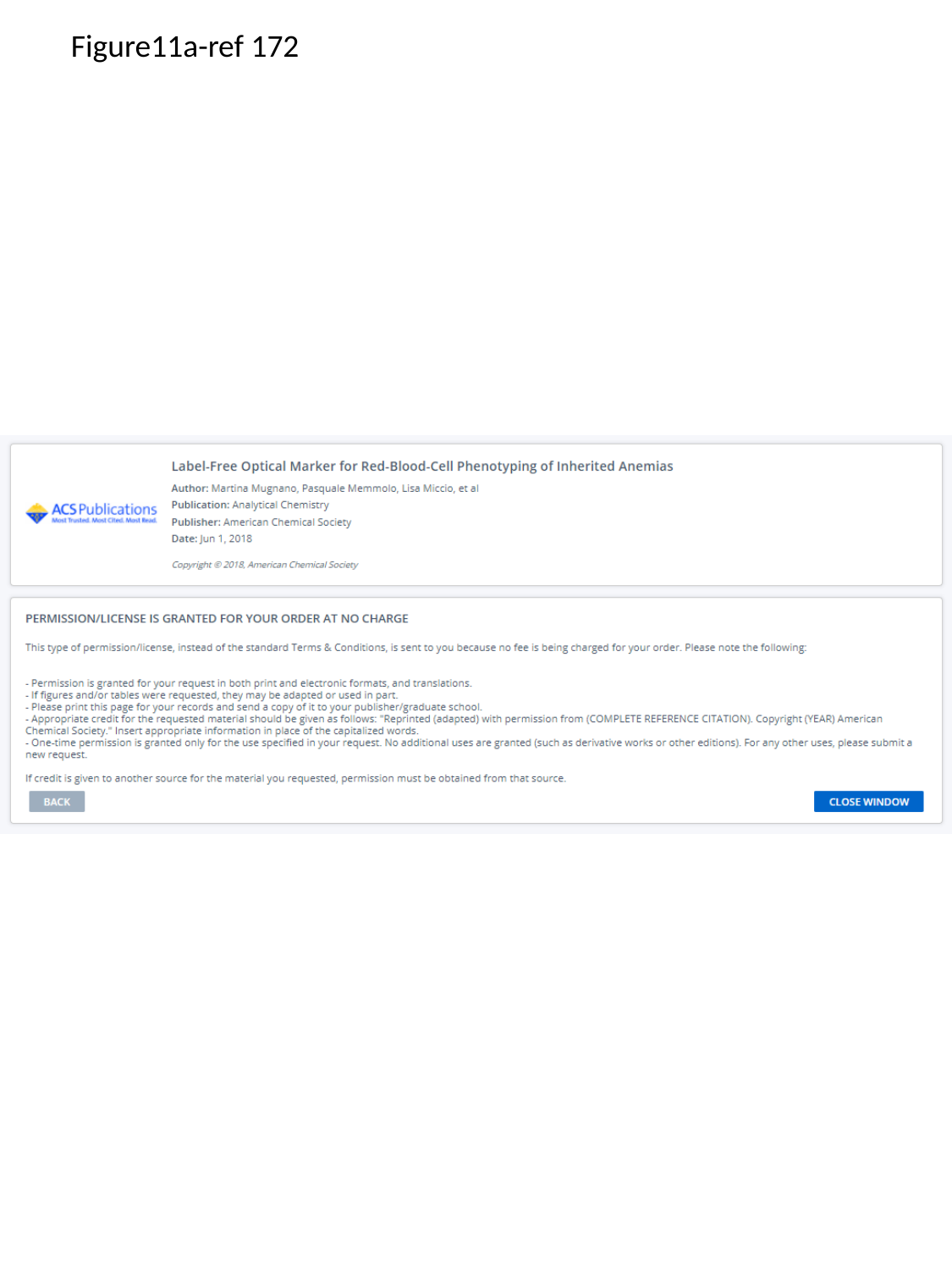

Figure11a-ref 172

## Slide 53
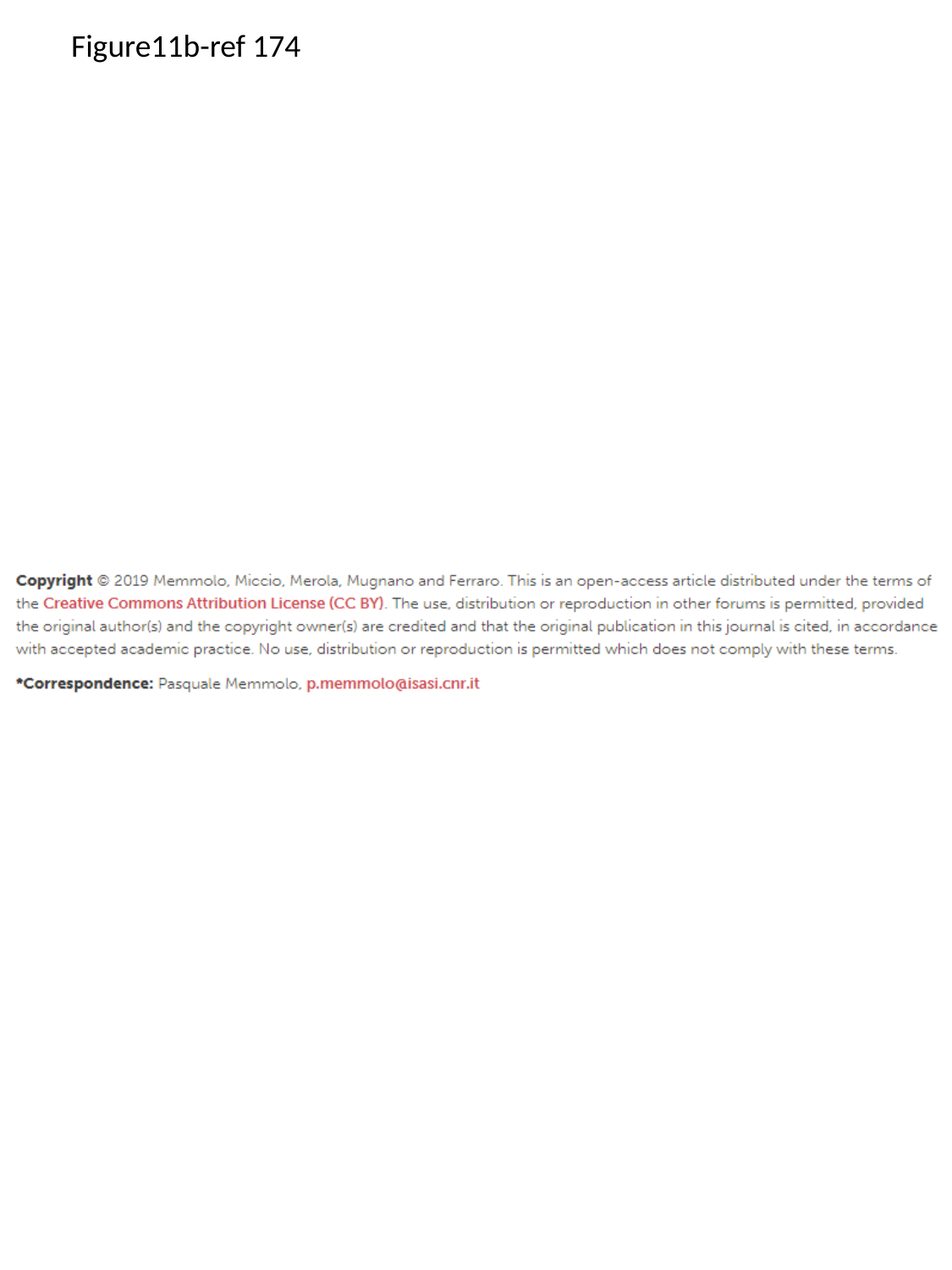

Figure11b-ref 174

## Slide 54
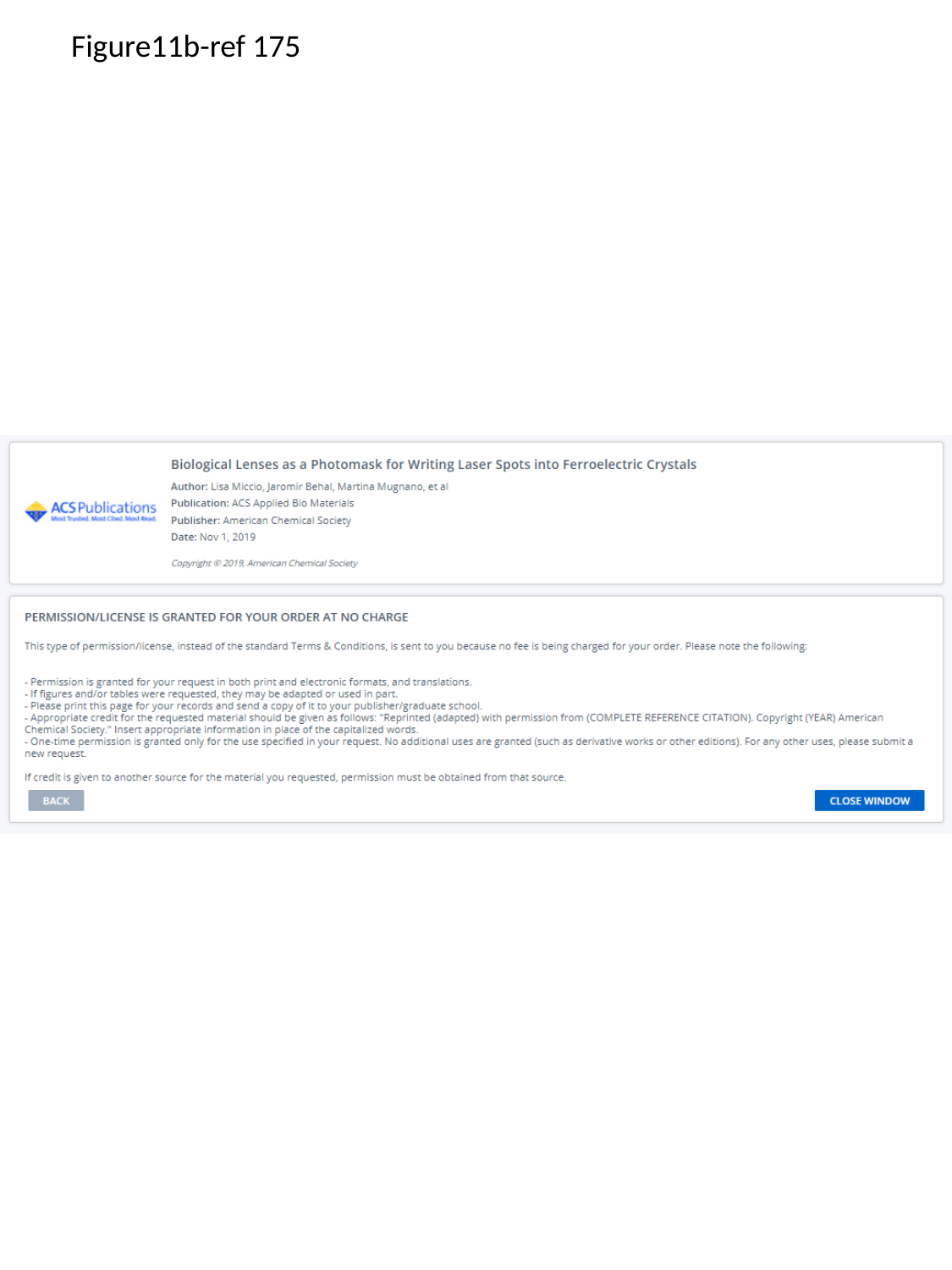

Figure11b-ref 175
